# Position-Specific Metabolic Probing and Metagenomics of Microbial Communities Reveal Conserved Central Carbon Metabolic Network Activities at High Temperatures

**DOI:** 10.3389/fmicb.2019.01427

**Published:** 2019-07-05

**Authors:** Scott C. Thomas, Kevin O. Tamadonfar, Cale O. Seymour, Dengxun Lai, Jeremy A. Dodsworth, Senthil K. Murugapiran, Emiley A. Eloe-Fadrosh, Paul Dijkstra, Brian P. Hedlund

**Affiliations:** ^1^School of Life Sciences, University of Nevada, Las Vegas, NV, United States; ^2^Department of Biology, California State University, San Bernardino, CA, United States; ^3^Department of Energy Joint Genome Institute, Joint Genome Institute, Walnut Creek, CA, United States; ^4^Department of Biological Sciences, Center of Ecosystem Science and Society, Northern Arizona University, Flagstaff, AZ, United States; ^5^Nevada Institute of Personalized Medicine, University of Nevada, Las Vegas, NV, United States

**Keywords:** ^13^C stable isotope, isotopomers, heterotrophy, catabolic, anabolic, thermophile, hyperthermophile, diversity

## Abstract

Temperature is a primary driver of microbial community composition and taxonomic diversity; however, it is unclear to what extent temperature affects characteristics of central carbon metabolic pathways (CCMPs) at the community level. In this study, 16S rRNA gene amplicon and metagenome sequencing were combined with ^13^C-labeled metabolite probing of the CCMPs to assess community carbon metabolism along a temperature gradient (60–95°C) in Great Boiling Spring, NV. 16S rRNA gene amplicon diversity was inversely proportional to temperature, and *Archaea* were dominant at higher temperatures. KO richness and diversity were also inversely proportional to temperature, yet CCMP genes were similarly represented across the temperature gradient and many individual metagenome-assembled genomes had complete pathways. In contrast, genes encoding cellulosomes and many genes involved in plant matter degradation and photosynthesis were absent at higher temperatures. *In situ* ^13^C-CO_2_ production from labeled isotopomer pairs of glucose, pyruvate, and acetate suggested lower relative oxidative pentose phosphate pathway activity and/or fermentation at 60°C, and a stable or decreased maintenance energy demand at higher temperatures. Catabolism of ^13^C-labeled citrate, succinate, L-alanine, L-serine, and L-cysteine was observed at 85°C, demonstrating broad heterotrophic activity and confirming functioning of the TCA cycle. Together, these results suggest that temperature-driven losses in biodiversity and gene content in geothermal systems may not alter CCMP function or maintenance energy demands at a community level.

## Introduction

Temperature is a primary driver of taxonomic diversity of microbial communities (Cole et al., 2013; Sharp et al., 2014; Sunagawa et al., 2015; Power et al., 2018), with an observed maximum at 25°C and decreasing diversity as temperature becomes more extreme (Sharp et al., 2014). This temperature-driven diversity gradient provides an opportunity to assess the consequences of decreasing taxonomic diversity on ecosystem function (Swingley et al., 2012). On the one hand, several studies have suggested that taxonomic and functional diversity are not closely linked. For example, the Human Microbiome Project Consortium (2012) found core functional diversity to be shared by microbial communities in different locations on the human body, despite vast differences in taxonomic composition. Similarly, Sunagawa et al. (2015) showed that core functional diversity was conserved in the global ocean microbiome, despite spatial variability in taxonomic diversity, and that >73% of the ocean and human microbiome core functional gene content was shared. The existence of an ecosystem-independent core functional gene assemblage suggests conservation of ecosystem function across diverse microbial communities and physiochemical conditions (Sunagawa et al., 2015). On the other hand, the upper-temperature limit for photosynthesis is ~73°C (Brock, 1967; Cox et al., 2011; Boyd et al., 2012) and the upper-temperature limit for chemolithotrophic nitrite oxidation may be ~65°C (Lebedeva et al., 2005; Sorokin et al., 2012; Edwards et al., 2013), suggesting temperature can be a strong driver of not just taxonomic diversity but also functional diversity.

Contained in the global ocean and human microbiome core gene assemblages are genes necessary for glycolysis, the pentose phosphate pathway (PPP), and the tricarboxylic acid cycle (TCA) (Sunagawa et al., 2015). These “central carbon metabolic pathways” (CCMPs) are intimately linked to biological energy-generating reactions (catabolism) and to the synthesis of biomass precursors (anabolism) in heterotrophic organisms. If there is an ecosystem-independent core functional assemblage for microbial communities, then these pathways should be present in microbial communities inhabiting extreme environments as well. While metagenomic studies have demonstrated the conservation of ecosystem function at the genomic level in diverse ecosystems, these studies failed to address the activities directly.

**Figure 1:**
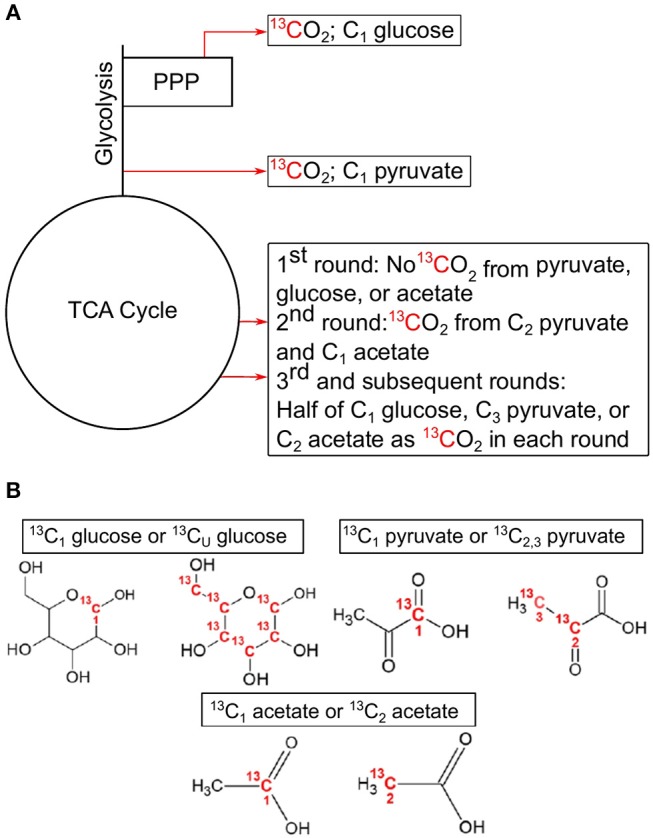
Simplified demonstration of decarboxylation events with associated position-specific isotopomers used in this study. Locations of decarboxylation events in central carbon pathways **(A)**, and the associated C position in the ^13^C-labeled compounds used in these experiments **(B)**. C1 from glucose is lost as CO_2_ in the pentose phosphate pathway with the synthesis of ribulose 5-phosphate by 6-phosphogluconate dehydrogenase or after multiple rounds in the TCA cycle. C1 from pyruvate is lost as CO_2_ in the formation of acetyl-CoA by pyruvate dehydrogenase. C2 and C3 of pyruvate are lost in multiple cycles of the TCA cycle. C1 from acetate is lost as CO_2_ in the 2nd cycle of the TCA cycle, while half of C2 is lost in each subsequent cycle. All CO_2_ producing reactions can produce ^13^CO_2_ from uniformly labeled ^13^C glucose. This example assumes a glycolytic flux through the central carbon pathways. TCA, tricarboxylic acid cycle; PPP, pentose phosphate pathway.

The activity of CCMPs in microbial communities can be assessed using position-specific ^13^C-labeled substrates and modeling metabolic flux using ^13^CO_2_ production rates (Dijkstra et al., 2011a,b,c, 2015; Hagerty et al., 2014; Figure 1). The use of position-specific ^13^C-labeled compounds (i.e., isotopomers) provides greater information than uniformly ^13^C-labeled substrates, allowing inferences to be made about the flux of carbon through CCMPs and the dynamics of catabolic and anabolic reactions (Dijkstra et al., 2011a,b; Leighty and Antoniewicz, 2013). For studying microbial community activity, this approach is superior to the classic ^13^C metabolic flux analysis based on amino acid labeling patterns (Wiechert et al., 2001), largely due to the difficulties of obtaining an isotopic steady state for all members of the community (Zamboni et al., 2009).

The principle governing using ^13^C-labeled isotopomers is that if a substrate is used solely for catabolic purposes, then carbon (C) from all positions in the substrate will be oxidized to CO_2_ in the same ratio they are found in the substrate (i.e., 1-^13^C: 2,3-^13^C (^13^C_1_:^13^C_2, 3_) pyruvate ratio equals 0.5). However, if anabolic reactions are taking place, then this ratio deviates from the hypothetical “catabolic only” ratio. For example, in the complete oxidation of pyruvate through CCMPs, C_1_ is released as CO_2_ as a result of pyruvate dehydrogenase or pyruvate-ferredoxin oxidoreductase activity, while C_2_ and C_3_ are oxidized to CO_2_ in the TCA cycle (Figure 1). Similarly, C_1_ of glucose is oxidized to CO_2_ during the formation of ribulose-5-phosphate via 6-phosphogluconolactonase in the PPP, while the other five carbons are oxidized by pyruvate dehydrogenase and the TCA cycle. If glucose is metabolized through glycolysis, then C_1_ of glucose can also be oxidized in the TCA cycle, but only after at least the third cycle, providing a greater opportunity for C_1_ to be sequestered in biomass. Thus, the relative rate of ^13^CO_2_ production from C_1_ of glucose [relative to uniformly ^13^C-labled (^13^C_U_) glucose] would be high when PPP is active and low during conditions of reduced PPP activity, assuming metabolites are being used for biomass production (Dijkstra et al., 2015). By comparing rates of ^13^CO_2_ production from specific isotopomers (e.g., ^13^C_1_ vs. ^13^C_2, 3_ pyruvate), information is gained on the relative activity of CCMPs and the catabolic and anabolic use of these compounds.

In the present study, we focused on a single terrestrial geothermal system, the Great Boiling Spring (GBS) system, located in northern Nevada, U.S.A. GBS is a circumneutral pH, NaCl-type geothermal system that harbors well-studied microbial communities rich in novel and yet-to-be-cultivated microorganisms (Dodsworth et al., 2012, 2013; Rinke et al., 2013; Becraft et al., 2016, 2017; Eloe-Fadrosh et al., 2016). Within this system, taxonomic diversity has been shown to be inversely proportional to temperature, concomitant with a shift from *Bacteria* to *Archaea* (Cole et al., 2013). Native communities in GBS have been shown to utilize a variety of organic electron donors for aerobic respiration (Murphy et al., 2013), and denitrification in GBS appears to be heterotrophic (Dodsworth et al., 2013). These studies, along with stable isotope natural abundance data from Yellowstone National Park (Schubotz et al., 2015; Jennings et al., 2017), point to the importance of both heterotrophy and autotrophy for high-temperature microbial communities. Yet, to our knowledge, the activity of CCMPs within terrestrial geothermal systems has not been addressed directly. This system provides an opportunity to study the functional consequences of temperature-driven losses in taxonomic diversity while minimizing other variables, such as water chemistry, photosynthetically active radiation, and geographic distance.

To examine the relationships between temperature-driven taxonomic diversity and the functioning of CCMPs, we combined ^13^C-based metabolic probing with analysis of 16S rRNA gene amplicons, shotgun metagenomes, and metagenome-assembled genomes (MAGs) derived from the same samples. This system was then used to test several hypotheses about relationships between temperature-driven losses in taxonomic diversity and ecosystem function: (1) genes associated with photosynthesis and degradation of complex organic matter will decrease at high temperature; (2) PPP gene abundance and ecosystem function will decrease at higher temperature, due to a relative increase in *Archaea* (Bräsen et al., 2014); (3), genes for other CCMPs and catabolic carbon use will be unaffected by temperature (Valentine, 2007; Sabath et al., 2013; Bräsen et al., 2014); (4) catabolic carbon use will increase with temperature, due to an increase in maintenance energy demand (Tijhuis et al., 1993; Price and Sowers, 2004); and (5) diverse TCA cycle intermediates will be used catabolically at high temperature.

## Materials and Methods

### Study Site

Three sediment sample locations within GBS (N40°39′41″ W119°21′58″) and one location from GBS 19 (~30 m from GBS) were selected to encompass a temperature gradient from 60–95°C. Sites were designated GBS 95, GBS 85, GBS 70, and GBS 60 (Figure S1; Table S1), and roughly correspond to previous study sites (Figure S1; Cole et al., 2013). GBS and GBS19 share a single deep subterranean source and have similar water chemistry (Table S1; Anderson, 1978).

### Field Measurements and Water Chemistry Analysis

Prior to water and sediment collection, temperature, conductivity, and pH were measured using a LaMotte pH5 Series pH/temperature meter (LaMotte, Chestertown, MD, USA). For major cation and anion analysis, water was filtered through 0.2 μm polyethersulfone (PES) filters (Pall Corp., USA) into 15 mL polypropylene tubes and frozen on dry ice. For trace element analysis, water was filtered through a 0.2 μm polyethersulfone (PES) filters (Pall Corp., USA) into acid-washed 150 mL Nalgene® bottles spiked with 400 μL 10% HNO_3_ (OmniTrace Ultra). Trace element samples were shaken and degassed three times, then stored at room-temperature until analysis. Field blanks, consisting of sterile Nanopure water processed in tandem with environmental samples, were collected at the time of sampling.

Ion chromatography (IC; Dionex DX-500 chromatography, Dionex Co., USA), direct current plasma optical emission spectrometry (DCP-OES; Beckman, USA), and inductively coupled plasma mass spectrometry (ICP-MS; Varian 820 quadrupole, Agilent Technologies Inc., USA) analysis for major anions, cations, and trace elements were performed as described by Huang et al. (2013).

### DNA Extraction and Illumina 16S rDNA Sequencing

The modified FastDNA Spin Kit for Soil (MP Biomedicals, Solon, OH, USA) (Dodsworth et al., 2011) was used for sediment DNA extraction (collected as described below) for both 16S rRNA gene and metagenomic sequencing. DNA was shipped to Micro-Seq Enterprises (Las Vegas, NV, USA), where the amplification and sequencing of the V4 region of the 16S rRNA gene for *Bacteria* and *Archaea* was performed using the Illumina MiSeq platform (San Diego, CA, USA), as described in Kozich et al. (2013) and a forward primer designed to increase coverage of *Archaea* (Hou et al., 2013). Sequences have been deposited to the NCBI Sequence Read Archive under project SRP059341 (GBS 60, SRX1055762; GBS 70, SRX1055763; GBS 85, SRX1055764; GBS 95, and SRX1055765).

Sequences were trimmed, assembled, ambiguous bases were removed and classified using a Bayesian classifier using Mothur according to the standard operating procedure provided by Kozich et al. (2013). Chimeras were identified in the aligned sequences using UCHIME with the Silva SEED database (v119). Following removal of chimeras, sequences were binned into operational taxonomic units (OTUs) above sequence identity of 97.0%. Taxonomic assignment was according to SILVA (v119) (Pruesse et al., 2012; Quast et al., 2013; Yilmaz et al., 2014). Manual curation was performed on several unclassified sequences using BLASTn, Greengenes, EzBioCloud (Yoon et al., 2017), and sequence cut-offs suggested by Yarza et al. (2014) for identification and naming of sequences belonging to candidate phyla.

Community statistics were calculated using rarefaction tool kit (RTK) (Saary et al., 2017) and phyloseq (McMurdie and Holmes, 2013). The OTU abundance table and taxonomy files from mothur were imported into phyloseq, alpha diversity measurements were carried out using “*estimate_richness”* function. For RTK, samples were rarefied based on GBS 95, which had the lowest number of reads (17,181). OTUs used in the SILVA assignments and the alpha diversity analyses were grouped at 97% sequence identity.

### Metagenomic Illumina Sequencing

Detailed descriptions for the processing, sequencing, and assembly of the metagenomes are publicly available from the Integrated Microbial Genomes and Microbiomes platform (IMG/M; https://img.jgi.doe.gov/m/) (Chen et al., 2019), under Gold Analysis Project IDs Ga0197142, Ga0197143, and Ga0197144 (GBS 60, GBS 70, and GBS 85, respectively). Briefly, isolated DNA for metagenomic sequencing was sequenced by the Joint Genome Institute (JGI) using shotgun Illumina HiSeq 2500-1TB, assembled using SPAdes version: 3.10.1, and annotated using the IMG Annotation Pipeline v.4.15.1 (Huntemann et al., 2016). JGI-identified 16S rRNA gene sequences contained in each metagenome were classified using Silva (v128). Metagenome sequencing from GBS 95 was not possible due to the low yield of DNA.

### Metagenome-Assembled Genomes (MAGs)

Genome binning was performed using MetaBAT (v0.32.4; Kang et al., 2015) with a 3,000 bp minimum contig cutoff, contig coverage information, and parameter “-superspecific” for maximum specificity.

Bin completeness and contamination were estimated using CheckM (Parks et al., 2015). Bins were deemed to be of high-quality if they were >90% complete, <5% contaminated, and contained a full rRNA operon and >18 tRNAs (Bowers et al., 2017). High-quality bins were analyzed using the Genome Taxonomy DataBase Toolkit and tools contained therein (GTDB-Tk, v0.1.1) for gene calling, protein sequence prediction, and taxonomic identification (Hyatt et al., 2010; Matsen et al., 2010; Eddy, 2011; Jain et al., 2018; Parks et al., 2018). 16S rRNA gene amplicons were matched with metagenome bins using BLAST+ (Camacho et al., 2009) in the web-based Galaxy platform (Cock et al., 2015). 16S rRNA gene amplicons were queried against each metagenomic bin using Megablast (Zhang et al., 2000; Morgulis et al., 2008).

### Assessing Metabolic Potential and Pathway Completeness

Protein sequences for high-quality bins and metagenomes were submitted individually to MAPLE (Metabolic and Physiological potential Evaluator, v2.3.1) (Takami et al., 2012b, 2016; Arai et al., 2018) for the evaluation of Kyoto Encyclopedia of Genes and Genomes (KEGG) functional module completion ratios (Takami et al., 2012b). Within MAPLE, the NCBI BLAST search engine was used with the single-direction best hit annotation method for KEGG Orthology (KO) assignment, using all organisms in the KEGG database. Whole-community module completion ratios were considered biologically feasible at *Q* < 0.5 (https://maple.jamstec.go.jp/maple/maple-2.3.1/help.html).

### NOISeq Differential Abundance and KEGG Mapping

KOs identified using the JGI IMG/M pipeline were analyzed using NOISeq-sim (Tarazona et al., 2011). To determine relative abundance of KOs, protein sequences with KEGG Orthology (KO) annotations were tabulated using the JGI-provided estimated gene copy number, normalized to unassembled metagenome size (GBS60, 23,572,233,708 bp; GBS70, 18,720,210,806 bp; and GBS85, 16,850,842,886 bp) using counts per billion bases, and log_2_-transformed using NOISeq v2.22.0 (Tarazona et al., 2011, 2015) in R v3.4.3 (R Core Team, 2017). To determine KO allele richness, genes with KO annotations were tabulated based on the JGI-provided gene count, normalized to assembled metagenome size (GBS 60, 461,589,717 bp; GBS 70, 297,862,735 bp; and GBS 85, 80,294,486 bp) using counts per billion bases, and log_2_-transformed as above. Differential abundance and richness analysis was performed using the NOISeq-sim function with the developer-recommended parameters for analyses of simulated replications with no pseudocount added to zero-count hits (Tarazona et al., 2011, 2015). The log_2_-transformed normalized abundance and normalized richness values were then mapped to relevant KEGG pathway diagrams (Kanehisa and Goto, 2000) and significant differences in KO abundance were noted, using a custom algorithm that combines the outputs of the KEGG reconstruct pathway and KEGG color pathway tools (Kanehisa and Goto, 2000; Kanehisa et al., 2012, 2017). The NOISeq-sim recommended probability value of 0.9 was used to indicate significant differences between any pairwise comparison (Tarazona et al., 2011, 2015). All possible pairwise comparisons were made.

Carbohydrate-active enzyme (CAZyme) (Lombard et al., 2014) annotation was performed on JGI IMG/M protein sequences using hmmer version 3.1b2 (Eddy, 1998) with the dbCAN database (Yin et al., 2012) as a reference. To determine relative abundance of CAZymes, CAZyme HMM hits for each of the metagenomes were tabulated using scaffold average coverage and normalized to unassembled metagenome size using counts per billion bases. To determine CAZyme allele richness, CAZyme HMM hits were tabulated without considering scaffold read-depth and then normalized to assembled metagenome size. Differential abundance and richness of the normalized HMM hits were analyzed as above using the NOISeq-sim function (Tarazona et al., 2011, 2015). Raw reads and assembled metagenomic contigs, as well as functional predictions, are available on the JGI IMG/M platform (Chen et al., 2019) under taxon object IDs 3300020153, 3300020139, and 3300020145 (GBS60, GBS70, and GBS85, respectively).

### ^13^CO_2_ Production Rate Experiments

Hot spring sediment slurry microcosms (top ~1 cm) were incubated *in situ* to monitor the production rate of ^13^CO_2_ from position-specific ^13^C-labeled isotopomer pairs and uniformly ^13^C-labeled compounds over an 8-h period. Subsamples of the sediment slurry were aseptically transferred to 2 mL centrifuge tubes and immediately frozen on dry ice for later DNA extraction for 16S rRNA and metagenome work, or were dispersed for the ^13^CO_2_ production rate experiments, as detailed below. Slurries were kept near *in situ* temperatures by placing the bottles in the hot spring during preparation and incubation of samples.

To prepare microcosms, ~20 mL of sediment slurry was aseptically dispersed into sterile, pre-warmed 165 mL serum bottles using a modified 10 mL Oxford BenchMate™ pipette (St. Louis, MO, USA). After slurry addition, serum bottles were capped and sealed and then 1 mL of ^13^C-labeled substrate was added using a 1 mL syringe and 25 G needle. Each ^13^C substrate incubation was performed in triplicate except for “killed,” mercury- or glutaraldehyde-treated controls (described below), which were performed in duplicate to limit sample numbers. Microcosms were secured in a wire cage, covered with foil, and completely submerged in the hot spring. Temperature was monitored throughout the experiment using a Traceable® Ultra Waterproof Thermometer (VWR, Radnor, PA, USA) fixed to the wire cage. At the end of the experiment, microcosms were kept on wet ice until sediments could be dried and weighed in the lab. ~10 mL headspace samples were taken ~2, 4, and 8 h after ^13^C-labeled substrate addition. Atmosphere samples (*n* = 3) were collected onsite to determine a time-0 ^13^CO_2_ delta value. Samples were stored in collection syringes until analysis, as described below.

Isotopomer pairs consisted of filter-sterilized solutions (21.4 μmol substrate-C mL^−1^) of sodium pyruvate (1-^13^C and 2,3-^13^C), sodium acetate (1-^13^C and 2-^13^C), and glucose (1-^13^C and uniformly (U) ^13^C-labeled) (99 atom fraction %; Cambridge Isotope Laboratories, Andover, Massachusetts). For the 85°C site, uniformly ^13^C-labeled citrate, L-serine, L-cysteine, L-alanine, and succinate (99 atom fraction %; Cambridge Isotope Laboratories, Andover, Massachusetts) were also used at 0.05 mg/mL to evaluate the diversity of organic compounds catabolized by the community. Mercuric chloride and glutaraldehyde were used as poisons [1.0 mM and 2.0% (vol/vol), respectively], individually and in combination, for monitoring abiotic decarboxylation of uniformly ^13^C-labeled substrates. Mercury was tested at all four sites, while glutaraldehyde was tested at 60 and 95°C sites only.

### ^13^CO_2_ Analysis

Headspace samples were analyzed for ^13^CO_2_ on a Picarro 2101-*i* CO_2_ and CH_4_ isotope spectrometer (Picarro Inc, Sunnyvale, CA, USA) by first spiking samples with a high concentration of CO_2_ and then diluting with CO_2_-free air. This step was necessary to ensure that CO_2_ concentrations were within the working range of the Picarro (300–2,000 μmol mol^−1^) and to provide enough gas volume for a 10-min. sample analysis. Spikes were performed by filling a Mason jar (473 mL) with atmosphere and injecting 10 mL of pure CO_2_ through a rubber stopper inserted in the Mason jar lid. For each sample, a volume of gas from the CO_2_-spiked Mason jar was added at a volume matching the microcosm gas sample volume, resulting in a 1:1 ratio of sample and CO_2_-spiked atmosphere. A maximum of four CO_2_-spiked aliquots were taken from the Mason jar before the jar was opened, vented, and refilled with CO_2_-spiked atmosphere. Both CO_2_-spiked atmosphere and samples were injected into a Tedlar® bag (0.5 L) and CO_2_-free gas was used to increase the mixed sample volume. Data from the Picarro were recorded as 30-second averages of δ^13^CO_2_ over a period of near-constant delta readings. Data were expressed as atom percent excess (APE) per hour (relative to time zero measurements).

## Results

### Field Measurements and Water Chemistry

pH (7.17–7.52), conductance (7.47–7.68 mS), major ions, and trace elements were similar at all four sites along the temperature gradient (Table 1, Table S1). *In situ* temperatures, measured by tracing thermometers during the experiments, varied by ~4.0°C around the target sample temperatures used as site identifiers (i.e., GBS 60, GBS 70, GBS 85, and GBS 95; Table 1).

**Table 1:** Physical parameters of each site.

**Site**	**Recorded minimum temperature (^**°**^C)**	**Recorded maximum temperature (^**°**^C)**	**pH**	**Conductance (mS)**
95°C	92.5	96.5	7.17	7.68
85°C	83.2	86.6	7.23	7.58
70°C	68.9	72.2	7.50	7.58
60°C	57.8	62.1	7.52	7.47

*See Table S1 for cation, anion, and trace element analysis for water samples from each site*.

### Taxonomic Diversity

Analysis of filtered 16S rRNA gene amplicons showed that observed richness ranged from 113 to 491 species-level OTUs (3%), while Chao1 estimated richness values ranged from 291.75 to 833.16 (Table 2). Simpson, inverse Simpson, and Fisher's alpha diversity ranged from 0.69 to 0.96, 3.27 to 26.05, and 16.01 to 87.53, respectively (Table 2). Shannon diversity ranged from 1.61 to 4.01 (Table 2). All diversity metrics decreased as temperature increased, although the GBS 85 site consistently demonstrated the lowest values (Table 2; Table S2).

**Table 2:** Non-rarefied 16S rRNA gene amplicon-based diversity statistics.

**Site**	**Observed richness**	**Chao1 (SE)**	**Shannon**	**Simpson**	**Inverse Simpson**	**Fisher**
95°C	211	390.08 (51.97)	2.71	0.878	8.16	33.53
85°C	113	291.75 (67.71)	1.61	0.694	3.27	16.01
70°C	326	536.07 (49.57)	3.73	0.957	23.37	55.17
60°C	491	833.16 (68.83)	4.01	0.962	26.05	87.53

*SE, standard error of the mean*.

**Figure 2:**
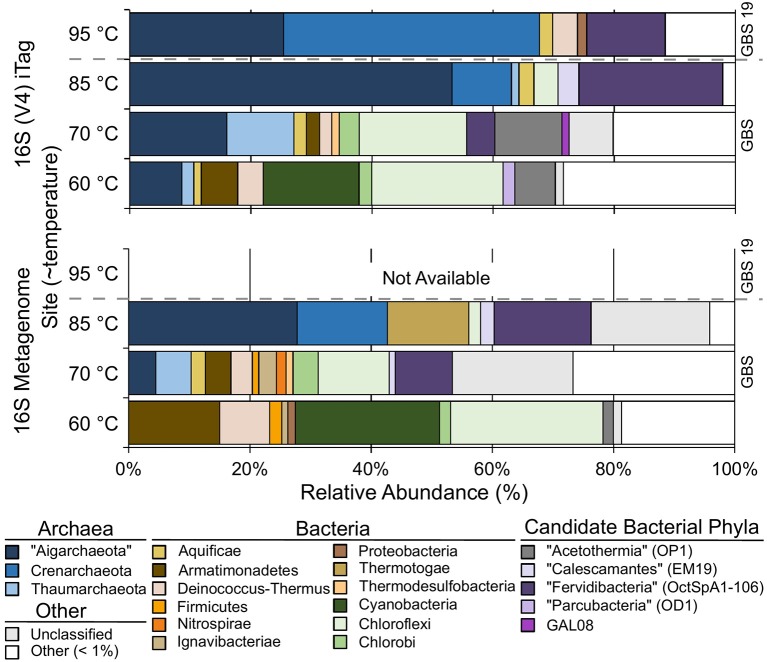
The relative abundance of phylum-level groups for each sample based on 16S rDNA Illumina Tags (iTags) of the V4 region compared with metagenome-derived 16S rDNA. Colored rows show the relative abundance of phyla present at >1% and correspond to the site label on the left. Unclassified sequences are represented as a single group; see Table S3 for information on individual unclassified groups and their abundance. Gray dashed bar indicates GBS 19 is a separate geothermal pool from the main pool. Metagenomic data from GBS 19 is not available due to low DNA yield.

Both 16S rRNA gene amplicons and metagenomes showed that *Archaea* were more abundant at high temperatures, and phototrophs were not detected at high-temperature sites (Figure 2; Table 3). The highest proportion of rare sequences (<1% relative abundance) was found at low-temperature sites (Figure 2; Table 3). Many abundant phyla contained limited numbers of OTUs (Table 3; Table S4), and many OTUs were unique to a single temperature (Table 3; Table S3). At all temperatures, many of the abundant OTUs represented high-level taxa (i.e., phyla, classes, and orders) with no cultivated members.

**Table 3:** Prominent phyla, relative abundance of associated OTUs, and associated MAGs.

				**% Relative abundance**			
**Phylum**	Taxonomic classification^*^	**OTU#**	**Bin ID#**	**GBS 60**	**GBS 70**	**GBS 85**	**GBS 95**
“Acetothermia” (OP1)	“Acetothermia” (p)	0022		0	4.5	0	0
	*Candidatus* Acetothermus (g)	0004		6.7	6.7	0	0
“Aigarchaeota”	*Candidatus* Caldiarchaeum (g)	0005		6.5	5.8	0	0
	*Candidatus* Calditenuis (g)	0001		0	6.1	48.4	0
	Group G2 (g)	0009	60_51, 70_36, 85_8	2.1	4	4.8	0
	Group G4 (g)	0003		0	0	0	25.4
Chloroflexi	Anaerolineaceae (f)	0046		1.1	0	0	0
	Caldilinea (g)	0049		1	0	0	0
	Chloroflexi (p)	0011		0	9.6	0	0
	Chloroflexi (p)	0018		4.8	0	0	0
	Chloroflexi (p)	0027	70_32	0	2.6	0	0
	Chloroflexi (p)	0028		2.2	0	0	0
	Chloroflexi (p)	0031	60_31, 70_19	0	2.3	0	0
	Chloroflexi (p)	0036		1.6	0	0	0
	Chloroflexus (g)	0023		3.8	0	0	0
	Roseiflexus (g)	0014		7.1	0	0	0
	*Thermoflexus hugenholtzii* (sp.)	0016	85_2	0	3.2	4	0
Crenarchaeota	Desulfurococcaceae (f)	0025		0	0	0	3.6
	Desulfurococcaceae (f)	0029		0	0	0	2.8
	Desulfurococcaceae (f)	0037		0	0	0	2.1
	Desulfurococcales (o)	0013		0	0	0	9.7
	Ignisphaera (g)	0006		0	0	0	14.5
	Ignisphaera (g)	0019		0	0	0	6.3
	Sulfophobococcus (g)	0038		0	0	0	2
	Thermoprotei (c)	0012	85_9	0	0	9.8	0
	Thermoprotei (c)	0051		0	0	0	1.3
Cyanobacteria	Cyanobacteria (p)	0007		10.2	0	0	0
	Cyanobacteria (p)	0017	60_40	5.6	0	0	0
“Fervidibacteria”	*Candidatus* Fervidibacter (g)	0002		0	4.7	23.7	13
Thaumarchaeota	*Candidatus* Nitrosocaldus (g)	0008		0	9.6	1.2	0
	*Candidatus* Nitrosocaldales (o)	0041		0	1.5	0	0
	Thaumarchaeota (p)	0032		2	0	0	0
Unclassified	Unclassified	0026		1.3	1.5	0	0
	Unclassified	0039		0	1.7	0	0
	Unclassified	0040	70_11	0	1.6	0	0
	Unclassified	0042		0	1.1	0	0
	Unclassified	0044		0	1.3	0	0
Other (<1%)^**^				28.4	20.2	2.1	11.6

* *The lowest taxonomic level that was clearly definable is given. p, phylum; c, class; o, order; f, family; g, genus; sp., species. Taxonomy assigned by SILVA and manually, as described in methods. See Table S3 for 16S rRNA gene sequences for all OTUs found ≥1% relative abundance*.

** *Total relative abundance for OTUs found below 1% relative abundance. 16S rRNA gene amplicon OTUs were assigned to metagenomic bins using BLAST+ as described in methods. See Table S4 for the complete list of bins and their associated OTU sequences. “Aigarchaeota” genus-level groups are according to Hedlund et al. (2015)*.

*Chloroflexi* (~22%) and *Cyanobacteria* (~16%) were the most abundant groups at the GBS 60 site (Figure 2; Table 3; Table S3), including the genera *Roseiflexus* (OTU0014), *Chloroflexus* (OTU0023), and *Caldilinea* (OTU0049).

The GBS 70 site had four phylum-level groups >10% relative abundance: *Chloroflexi* (~18%), “Aigarchaeota” (~16%), “Acetothermia” (~11%), and *Thaumarchaeota* (~11%) (Figure 2; Table 3; Table S3). Known cultivated or uncultivated genera included the chemoheterotroph *Thermoflexus hugenholtzii* (OTU0016) (Dodsworth et al., 2014), the “Aigarchaeota” genera *Candidatus* Calditenuis aerorheumensis (OTU0001) (Beam et al., 2016) and *Candidatus* Caldiarchaeum subterraneum (OTU0005) (Nunoura et al., 2011), and the chemolithotrophic ammonia-oxidizing archaeon *Candidatus* Nitrosocaldus yellowstonii (Table S3; De La Torre et al., 2008).

The GBS 85 site was dominated by only two major groups, “Aigarchaeota” (~53%) and bacterial candidate phylum “Fervidibacteria” (~24%) (Rinke et al., 2013). *Candidatus* Calditenuis aerorheumensis (OTU0001) accounted for 48% of total abundance. “Fervidibacteria” OTU0002 was nearly identical (99% ID) to *Candidatus* Fervidibacter sacchari (Rinke et al., 2013). OTU0024 represented *Candidatus* Calescibacterium nevadense (Rinke et al., 2013; Becraft et al., 2016).

The GBS 95 site was dominated by *Crenarchaeota* (~42%) and “Aigarchaeota” (~25%). Most *Crenarchaeota* belonged to the order *Desulfurococcales*. “Aigarchaeota” consisted of a single OTU (0003) that was 99.4% identical to sequences found within Group 4 “Aigarchaeota” (Hedlund et al., 2015), and a MAG retrieved from Jinze Spring in Tengchong, China (Hua et al., 2018).

### Metabolic Potential in Metagenomes

**Figure 3:**
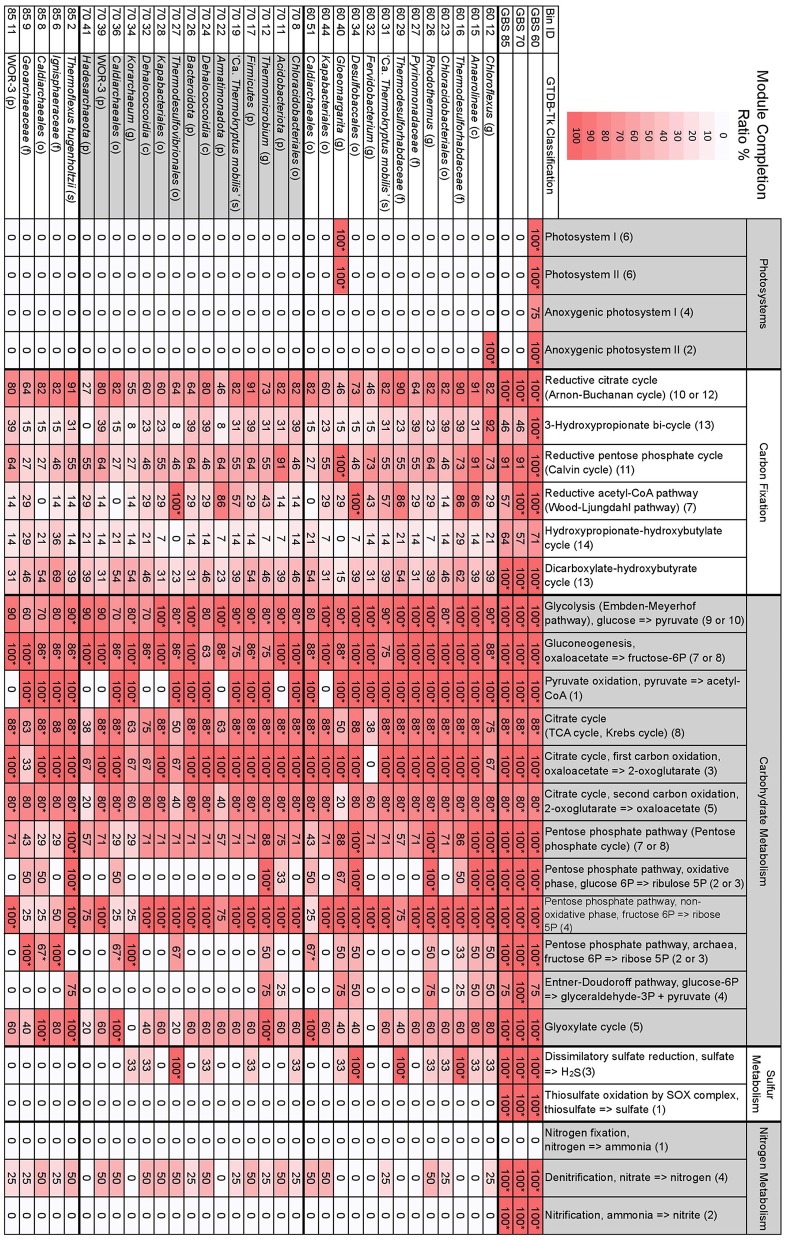
Selected MAPLE-derived module completion ratios for assembled metagenomes and GTDB-Tk taxonomically defined, high-quality MAGs. Heat map indicates the degree of a module's completion, ranging from 0 to 100%, for metagenomes or MAGs. Module completion ratios have been rounded to the nearest whole number. ^*^The associated Q-value is below 0.5 and is deemed biologically feasible. The first two-digit number in the Bin ID column indicates the metagenome the bin is from. Letters in parentheses for taxonomic classification indicate p, phylum; c, class; o, order; f, family; g, genus; s, species. Numbers in parentheses for individual modules indicate the number of components included in the module. See Table S4 for full GTDB-Tk classification of MAGs. See Table S5 for all modules, KEGG pathway IDs, and number of components comprising a single module.

**Figure 4:**
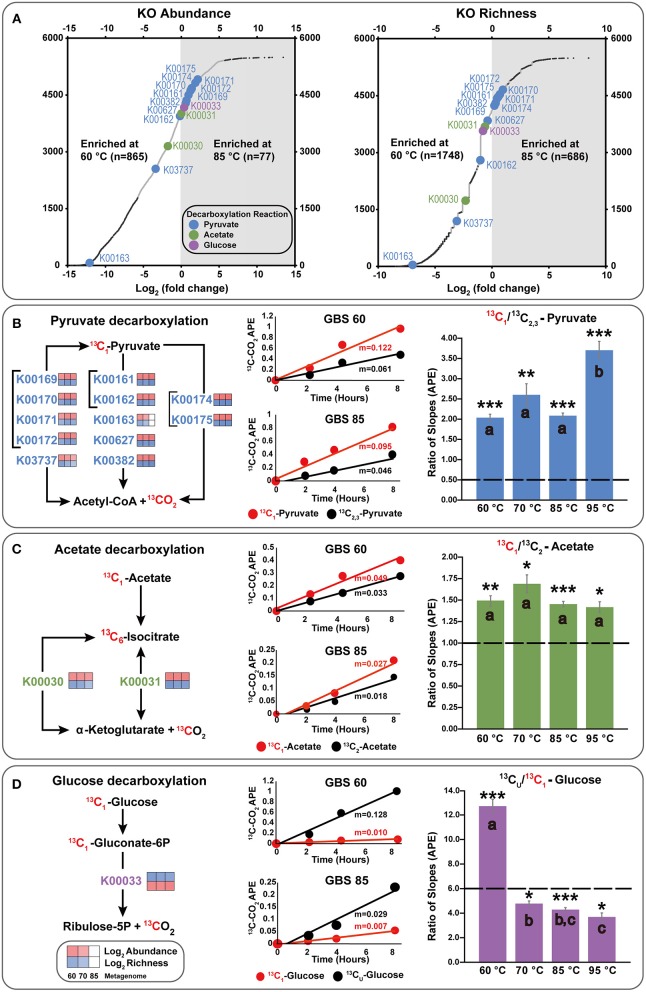
Integration of metagenome data and isotopomer experiments, focusing on key decarboxylation reactions. **(A)** Differential abundance and richness plots for KOs in GBS 60 and GBS 85 metagenomes. Large dots indicate KOs involved in decarboxylation reactions involving the C1 position of pyruvate, acetate, or glucose. Small dark dots indicate KOs that were significantly differentially abundant or rich. All data are shown in Table S6. **(B–D)** KOs involved in the production of CO_2_ from the C1 position of substrates. The left side shows simplified KEGG pathways with log_2_ abundance and richness heatmaps for GBS 60, GBS 70, and GBS 85. The middle shows ^13^CO_2_ production rates from isotopomer pairs for the GBS 60 and GBS 85 sites. APE, atom percent excess. The right demonstrates the catabolic and anabolic use of isotopomer pairs. Graphs indicate the ratio of the slopes (*n* = 9) for APE of ^13^C of CO_2_ from the isotopomer microcosms. Dashed line, ratio for complete oxidation; error bar, standard error of the mean; ^*^0.001; ^**^0.0001; ^***^0.00001 significantly different from complete catabolic use (*p* < 0.05; one-sample *t*-test); shared letters within an isotopomer group indicate that they are not distinguishable (ANOVA, Tukey HSD, and Welch *t*-tests).

KEGG module completion ratios (MCR) showed that the metagenomes had complete or near-complete CCMPs, with most modules being biologically feasible (*Q* < 0.5) (Figure 3). The only modules in central carbohydrate metabolism that were not biologically feasible were the Entner-Doudoroff pathway (KEGG module M00008) and the semi-phosphorylative Entner-Doudoroff pathway (M00633) in the GBS 60 and GBS 85 metagenomes (75% MCR, *Q* > 0.5), despite having 100% MCR (*Q*-value, 0) in the GBS 70 metagenome (Figure 4; Table S5). This was due to the absence of phosphogluconate dehydratase (K01690, EC 4.2.1.12). An alternative module for the semi-phosphorylative Entner-Doudoroff pathway (M00308) was biologically feasible in all metagenomes (Table S5).

The number of complete KEGG modules for carbon fixation pathways decreased as site temperature increased, with GBS 60 having 10 pathways with 100% MCR, GBS 70 having seven, and GBS 85 having six, out of a total of 15 modules (Figure 3; Table S5). The reductive citrate cycle (Arnon-Buchanan cycle) and dicarboxylate-hydroxybutyrate cycle had 100% MCR in all metagenomes. The reductive acetyl-CoA pathway (Wood-Ljungdahl pathway) and the 3-hydroxypropionate bi-cycle both had 100% MCR in GBS 60 and GBS 70 but were not biologically feasible in GBS 85. The reductive pentose phosphate cycle (Calvin cycle) and photosynthetic complexes and pathways had 100% MCR in GBS 60, but were not biologically feasible in GBS 70 (90.9%) and GBS 85 (90.9%). The hydroxypropionate-hydroxybutyrate cycle was not biologically feasible in any of the metagenomes.

Dissimilatory sulfate reduction, thiosulfate oxidation by the SOX complex, denitrification, and nitrification had 100% MCR in all three metagenomes, while no components for nitrogen fixation were found (0% MCR) in any of the metagenomes. MCRs for structural complexes associated with sulfate (M00185) and sulfonate (M00436) transport, and a urea transport system (M00323), all decreased as temperature increased (Table S5). The potential ability to use methanol as a substrate for methanogenesis (M00356) decreased with increasing site temperature (Table S5). Pectin degradation (M00081) had 100% MCR in GBS 60 but 0% MCR in GBS 70 and GBS 85 (Table S5). Modules involved with the biosynthesis of secondary metabolites were largely absent from all metagenomes (Table S5).

### Metabolic Potential in MAGs

A total of 167 MAGs were obtained from the three metagenomes of which 33 were considered high quality (Bowers et al., 2017). High-quality bins were taxonomically identified with the GTDB-Tk: 13 from GBS 60, 15 from GBS 70, and 5 from GBS 85 (Figure 3; Table S4). Most contained a 16S rRNA gene sequence identical to a 16S rRNA gene from the amplicon analysis (Table 3; Table S4). High-quality MAGs spanned eight bacterial phyla, one candidate bacterial phylum, and two archaeal phyla, according to the Genome Taxonomy Database (Parks et al., 2018): Acidobacteriota (4 bins), Armatimonadota (1), Bacteroidota (6), Chloroflexota (6), Cyanobacteriota (1), Desulfobacterota (3), Firmicutes (1), Nitrospirota (1), WOR-3 (2) (Baker et al., 2015), Crenarchaeota (6) (including “Aigarchaeota” and Thaumarchaeota), and Hadesarchaeota (1).

Bin 40 from GBS 60 (60_40), belonging to the genus *Gloeomargarita*, was the only bin to show 100% MCR for photosystems I and II as well as NAD(P)H:quinone oxidoreductase (M00145) (Figure 3; Table S5). Bin 60_40 also had 100% MCR for the reductive pentose phosphate cycle (Calvin cycle). Bin 12 from GBS 60 (*Chloroflexus* sp.) was the only bin to show 100% MCR for anoxygenic photosystem II. No components were found for anoxygenic photosystem I in any of the bins (0% MCR). All other bins had 0% MCR for all photosystems.

Glycolysis, pyruvate oxidation to acetyl-CoA, the TCA cycle, and the 1st and 2nd carbon TCA cycle oxidations were biologically feasible in most bins (Figure 3). In bins where pyruvate oxidation to acetyl-CoA was not found, gluconeogenesis was biologically feasible (Figure 3). The full PPP (M00004), including both oxidative (M00006) and non-oxidative phases (M00007), were biologically feasible in only five bins (all modules, 100% MCR) (60_12, 60_15, 60_26, 60_34, 85_2), while the non-oxidative phase (M00007) had 100% MCR in most bins (24 bins) (Figure 3). The archaeal PPP (M00580) was biologically feasible in six bins (60_51, 70_34, 70_36, 85_6, 85_8, 85_9), with three belonging to the order *Caldiarchaeales* [Genus 2 Hedlund et al., 2015]. The Entner-Doudoroff pathway (M00008) was not biologically feasible in any of the bins (Figure 3). The non-phosphorylative Entner-Doudoroff pathway (M00309) was biologically feasible in only one bin (85_6) (Table S5), belonging to the *Crenarchaeota* family *Ignisphaeraceae*.

### KO and CAZyme Abundance and Richness in Metagenomes

A total of 5,874 KOs and 358 CAZymes were identified in the three metagenomes from the 60, 70, and 85°C sites (Tables S6, S7). GBS 60 contained 5,442 KOs and 329 CAZymes, with total estimated gene copies of ~9,001,975 and ~776,603, respectively. GBS 70 contained 4,852 KOs and 299 CAZymes, with total estimated gene copies of ~7,677,420 and ~629,856, respectively. GBS 85 contained 2,995 KOs and 177 CAZymes, with total estimated gene copies of ~8,403,495 and ~540,463, respectively. Thus, the abundance and richness (i.e., the total number of different genes in each KO) of both KOs and CAZymes both decreased as a function of increasing temperature, in step with the pattern of taxonomic richness (Figure 4A; Tables S6, S7).

After normalization, NOISeq-sim analyses yielded 1,294 total KOs and 62 CAZymes that were differentially abundant for any pairwise comparison (Tables S6, S7), and 2,835 KOs and 134 CAZymes that were differentially rich (Tables S8, S9), with a probability of 0.9 or greater of being in any pairwise comparison. Of these 2,835 KOs, 1,047 were significant in both richness and abundance, 247 were unique to abundance, and 1,788 were unique to richness (Figure S2; Table S10). Of the 134 CAZymes, 48 were significant in both richness and abundance, 15 CAZymes were unique to abundance, and 86 CAZymes were unique to richness (Figure S2; Table S10). Figure 4A shows an example of the difference in abundance and richness of KOs for the GBS 60 to GBS 85 comparison. The plot shows that KOs were more abundant and richer in the GBS 60 metagenome (865 and 1,748), compared with the GBS 85 metagenome (77 and 686).

Many KOs significantly more abundant in GBS 70 and GBS 85 than in GBS 60 were archaeal KOs involved in information processing (e.g., transcription, translation, genome replication, recombination, and DNA repair) (Table S6). For example, archaeal KOs associated with tRNA synthesis, DNA repair, DNA and RNA polymerases, ribonucleases, and ribosomal proteins were more abundant at higher temperature sites. KOs associated with archaeal CRISPR proteins were also more abundant at higher temperature sites (e.g., K19074 and K07725).

Richness for KOs involved in information processing generally followed the same pattern as abundance, with archaeal-associated KOs showing greater richness in metagenomes from high- temperature sites (Tables S6, S8). For example, K03170, reverse gyrase, was more rich at GBS 70 and GBS 85 than GBS 60 (Table S8). In contrast, K02470, DNA gyrase, was less rich in GBS 85 than GBS 60 (Table S8).

KOs associated with starch and glycogen biosynthesis and utilization [e.g., K01176, K05343, K01208, K16147, and K16149 (89.8% probability)] were significantly less abundant at GBS 85 than GBS 60. KOs associated with archaeal flagella increased in abundance as site temperature increased and were more abundant in GBS 85 than GBS 70 (e.g., K07332, K07333), while KOs associated with pili were significantly less abundant at GBS 85 than GBS 60 [e.g., K02658, K02660, and K02662 (89.8% probability)]. Genes associated with the biosynthesis of heat-stable lipids were more rich at GBS 85 than GBS 60: K13787, a type-I geranylgeranyl diphosphate synthase involved in archaeal and bacterial ether-linked lipid biosynthesis (Vandermoten et al., 2009) (abundance significance 60-85 88.8%); K17105, a geranylgeranylglycerol-phosphate geranylgeranyltransferase also involved in archaeal ether-linked lipid biosynthesis (abundance significance 60–85, 89.7%); and K17104, an archaeal phosphoglycerol geranylgeranyltransferase (abundance significance 60–85, 89.8%). KO K13789, a type-I geranylgeranyl diphosphate synthase involved in bacterial and plant ether-linked lipid biosynthesis (Vandermoten et al., 2009), was less rich in GBS 85 than in GBS 60 or GBS 70. KOs involved in lipopolysaccharide (LPS) transport and biosynthesis were less abundant in GBS 85 than in GBS 60 [e.g., K09695, K09694, K19428, and K04744 (89.7% probability)]. Multiple ABC transporters were significantly more abundant at GBS 60 than GBS 85 (Table S6). The archaeal cytoskeletal element crenactin, KO K18641 (Ettema et al., 2011), was more rich in GBS 70 and GBS 85 than in GBS 60 (abundance 60–85, 89.8%).

KOs associated with photosynthesis were significantly more abundant and richer in GBS 60 than in GBS 70 or GBS 85 (>40 KOs). In many cases (>40 KOs), GBS 70 and GBS 85 did not contain any copies of the genes. Many of these KOs were associated with photosystems I and II, chlorophyll biosynthesis, and photosynthetic reaction centers. KOs K01601 and K01602, the large and small chains of ribulose-bisphosphate carboxylase, were more abundant in GBS 60 and GBS 70 than in GBS 85. KO K14138, acetyl-CoA synthase, involved in the Wood-Ljungdahl pathway, was more abundant in GBS 60 and GBS 70 than in GBS 85. In contrast, many KOs associated with the dicarboxylate-hydroxybutyrate cycle and hydroxypropionate-hydroxybutyrate cycle were significantly less abundant at GBS 60 than GBS 85 (e.g., K14465, K14467, K15016, K15020, and K15038).

Few KOs involved in CCMPs were differentially abundant between metagenomes. For example, Figure 4A shows that enzymes carrying out key decarboxylation reactions in the CCMPs were similarly abundant and rich in GBS 60 and GBS 85 (Figures 4A–D). In cases where differences were found, functionally redundant KOs were identified. For example, K00163 is not necessary for pyruvate decarboxylation when K00161 and K00162 are present (Figures 4A,B). Entire CCMP KEGG maps for CCMP functions are shown in Figure S2 and showed similar trends. For example, K01810 is a glucose-6-phosphate isomerase assigned to EC 5.3.1.9 and was significantly less abundant in GBS 85 than in GBS 60 and GBS 70 (Table S6). Yet, KOs K06859, K13810, and K15916, which are also assigned to EC. 5.3.1.9, were found across all three metagenomes with non-significant differences (Table S6). K01622, a fructose 1,6-bisphosphate aldolase/phosphatase thought to be an ancestral gluconeogenic enzyme responsible for removing heat-labile triosephosphates in *Archaea* and some *Bacteria* (Say and Fuchs, 2010; Takami et al., 2012a; Bräsen et al., 2014) was more abundant in GBS 85 than in GBS 60 (Table S6), with richness being significantly higher in GBS 85 than in GBS 60 or GBS 70 (Table S8).

The 62 CAZymes determined to be significantly differentially abundant (Table S7) in any pairwise comparison were classified into CAZy families: carbohydrate binding modules (CBM) (9), glycosyltransferases (GT) (6), glycoside hydrolases (GH) (38), polysaccharide lyases (PL) (8), and dockerins (1). The majority decreased in abundance with increasing site temperature. CAZymes associated with peptidoglycan degradation were more abundant in GBS 60 (e.g., GH 73, GH102, and GH104) than GBS 85, and PL9_4 was significantly more abundant at GBS 85 than GBS 70. Family GT8, involved in LPS biosynthesis, was less abundant in GBS 85 than in GBS 60. Some families involved in cellulose degradation decreased in abundance with increasing site temperature and were less abundant in GBS 85 than GBS 60 (e.g., CBM8, CBM63, GH5_46, GH94, and dockerin). Families and sub-families involved in starch degradation and synthesis tended to be significantly more abundant in GBS 60 than GBS 85 (e.g., CBM20, CBM21, GH13_2, GH13_3, GH13_10, GH13_21, GH13_26, GH13_36, GH65, GH88, and GH128), including sub-family GH13_7 which contains characterized α-amylases from *Archaea* (*Pyrococcus* sp., *Thermococcus* sp.). Only six families and subfamilies were significantly more abundant at GBS 85 than GBS 60 (CBM29, GH52, GH96, GH122, PL14_3, and PL22_2). GH122 has a single characterized protein from *Pyrococcus furiosus*, described as an α-glucosidase with broad substrate specificity for α-glucan carbohydrates (Comfort et al., 2008).

An expanded visualization and discussion of differentially abundant KOs can be found in Figure S2.

### ^13^C-Based Metabolic Activity

Three different poisoned controls were tested in an effort to best differentiate biotic and abiotic decarboxylation activity: glutaraldehyde, mercury, and both glutaraldehyde and mercury. The controls varied in terms of suppressing ^13^CO_2_ production. Delta ^13^CO_2_ values were consistently higher in mercury-poisoned controls using uniformly labeled substrates than in glutaraldehyde-containing controls, with many mercury-poisoned controls producing equal or greater delta ^13^C values than the non-poisoned microcosms (Table S11). At the GBS 60 and GBS 95 sites, the only sites where glutaraldehyde was evaluated, glutaraldehyde-containing controls consistently produced lower delta ^13^C values than their substrate-paired samples and poisoned controls containing only mercury. Although a small amount of ^13^CO_2_ production was observed in glutaraldehyde-containing controls, they provided confidence that the majority of ^13^CO_2_ production from experimental microcosms could be attributed to biological activity.

**Figure 5:**
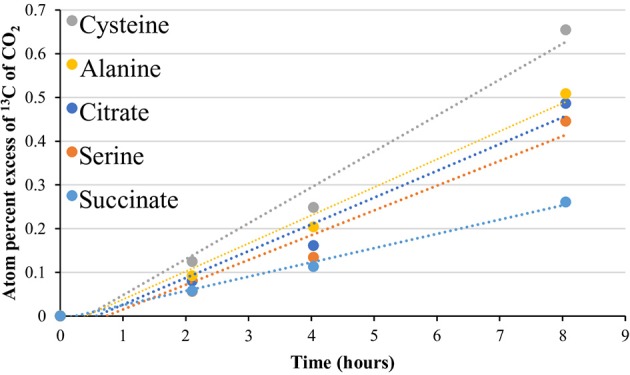
^13^CO_2_ production from uniformly labeled ^13^C citrate, serine, cysteine, alanine, and succinate. Symbols represent the average APE (*n* = 3) for a given compound at the given time. *R*^2^-values ranged from 0.961 to 1.0. The standard error is not shown for simplicity. See Table S11 for slope and linearity data for all uniformly ^13^C-labeled compounds and replicates for each site, and raw δ^13^C of CO_2_ values for the time course. Compounds were tested at the 85°C site only.

Figure 4 summarizes key decarboxylation reactions that are diagnostic of glycolysis (Figure 4B), the TCA cycle (Figure 4C), and the oxidative PPP (Figure 4D), with integrated data from the metagenomes (Figure 4A, left of Figures 4B–D) and the isotopomer experiments (figure middle and right of Figures 4B–D). At all sites, added ^13^C-labeled compounds resulted in a near-linear return of ^13^CO_2_ with low variability between replicates for the duration of the experiment (Figures 4B–D middle; Table S11). Within a site, all microcosms contained a similar quantity of sediment (Table S12). All paired isotopomer ratios showed significant differences from complete catabolic use at all sites (*p* < 0.05; one-sample *t*-test) (Figures 4B–D right, Table S13), but in most cases the similar ratios of the different decarboxylation reactions regardless of temperature demonstrated that the relative activities of the pathways were unaffected by temperature. An exception was the significantly greater decarboxylation ratio of C_U_: C_1_ glucose at GBS 60, with a general trend of a decreasing ratio as site temperature increased, resulting in GBS 95 being significantly lower than GBS 60 and GBS 70 (one-way ANOVA, Figure 4D right, Table S13). This indicates an increasing trend for the C_1_ glucose atom to be sequestered in biomass or fermentation products at the lower end of the temperature gradient. Pyruvate isotopomer ratios were significantly larger at GBS 95 than at the other sites, indicating an increased sequestration of C_2, 3_ pyruvate atoms in biomass or fermentation products. Broad heterotrophic activity was observed at the GBS 85 site, as indicated by strong, linear returns from all isotopomer pairs, and uniformly labeled amino acids and TCA cycle intermediates. ^13^C-labeled amino acids and TCA cycle intermediates were only administered at GBS 85 (Figure 5; Table S11).

## Discussion

### Taxonomic Diversity

Both amplicon-based and metagenome-derived 16S rRNA gene surveys demonstrated a decrease in diversity concomitant with an increase in temperature (Figure 2; Table 2), agreeing with previous diversity studies in GBS and globally (Cole et al., 2013; Sharp et al., 2014; Sunagawa et al., 2015; Power et al., 2018). As the temperature increased, a diverse, *Bacteria*-dominated community harboring phototrophs was replaced by an *Archaea*-dominated community consisting of a few thermophilic specialists (Figure 2), following well-known trends in geothermal systems (Hedlund et al., 2016). All sites had similar water chemistry and light exposure, suggesting temperature was the major driver in taxonomic shifts (Figure S1; Table S1).

All sites hosted a high proportion of candidate microbial taxa and unclassified sequences, conforming with previous studies at GBS (Cole et al., 2013; Rinke et al., 2013; Hedlund et al., 2015). In many cases, single OTUs from uncultivated groups accounted for large proportions of the community, highlighting the low diversity and phylogenetic novelty of this system. Additionally, several OTUs were only abundant in a single sample, suggesting distinct temperature niches and exemplifying the large taxonomic differences between samples. The proportion of “rare” 16S rRNA gene sequences (<1% abundance) was higher at low-temperature sites. High-temperature sites might not support a large rare-biosphere due to the energy demands of growth, maintenance, and survival at the physical extremes of life (Price and Sowers, 2004).

### Metabolic Potential

KEGG module completion ratios demonstrated the conservation of CCMPs along the temperature gradient for communities and individual MAGs. The fact that many MAGs contained complete CCMPs (Table 3; Table S4), suggests that C flux through these pathways can be facilitated by individual populations and are not reliant on metabolite swapping within the community.

All metagenomes contained a diversity of KOs and CAZymes. KOs associated with information processing were more abundant at higher temperatures, likely due to genome streamlining at higher temperatures (Sabath et al., 2013). Most of these were archaeal KOs, reflecting a shift to *Archaea*-dominated communities at high temperature. The large number of KOs and CAZymes that were found to be significantly different in richness only may reflect taxonomic differences, where the number of unique copies of a gene would be expected to decrease as taxonomic diversity decreases. Alternatively, the relative abundance of a gene may not decrease as taxonomic diversity decreases, if it provides for an essential function.

As expected, significant decreases in the abundance of genes associated with photosynthesis at high-temperature sites demonstrated the loss of a major energy source for autochthonous carbon fixation (Figure 3; Figure S2; Table S6). Differences in carbon fixation pathways were also observed with respect to temperature, with a lower number of modules for carbon fixation pathways predicted to be biologically feasible as site temperature increased (Figure 3; Table S5). Taken together, the reduction of taxonomic diversity concomitant with increasing site temperatures correlated with a loss in metabolic diversity with respect to primary production.

Genes associated with starch utilization, including an archaeal enzyme, decreased in abundance as temperature increased, suggesting this is not due to domain-level shifts in taxonomy. Genes associated with cellulosomes decreased in abundance with increasing temperature (e.g., dockerin). Cellulosomes are large extracellular protein complexes that may be too costly to produce and maintain in high-temperature environments. In fact, large membrane-bound or surface-associated enzyme complexes are not known to occur in hyperthermophiles (Hedlund et al., 2016). Although GBS 60 had the highest diversity of genes coding for ABC transporters, ABC transporters for various oligo- and monosaccharides, branched-chain amino acids and oligopeptides, and lipopolysaccharides and lipoproteins were present at relatively high abundance at all sites, suggesting heterotrophic flexibility and a conservation of function (Figure S2). Taken together, it appears that starch utilization is not prominent at high temperature, yet other allochthonous biomass from plants and insects may be important substrates for microbial communities in terrestrial geothermal springs (Schubotz et al., 2013).

Despite the temperature-driven decrease in taxonomic diversity, absence of photosynthesis, and shifts in genes associated with polymer degradation and information processing due to domain-level shifts in community composition, the functional potential of CCMPs remained consistent across sites (Figures 3, 4; Figure S2; Table S5), suggesting a conservation of CCMPs. The PPP was well-represented at the metagenomic level (Figures 3, 4D; Figure S2), despite the high relative abundance of *Archaea* at GBS 85, and representative MAGs (Table 3; Table S4) showing an incomplete PPP (Figure 3). Genes for the glyoxylate cycle were abundant at all sites, suggesting metabolic flexibility to adjust to the utilization of small carbon compounds (e.g., acetate; Figure S2). Abundance- and richness-populated KEGG maps for CCMPs (Figure S2) showed that the relative abundance of many KOs changed with temperature, yet the basic biochemistry was maintained (i.e., pathways were complete, demonstrated by populated EC numbers). This may be due to thermophilic *Archaea* having distinct enzymes for reactions in the CCMPs, with respect to *Bacteria* and *Eukarya*. This disparity may be due to pathway optimization for reduced protein cost and minimization of C and energy loss from thermolabile metabolites being favored over ATP yield, yet the basic biochemistry carried out by CCMPs remains similar (Flamholz et al., 2013; Bräsen et al., 2014). The conservation and diversity of CCMPs combined with shifts in genes associated with thermophily (e.g., DNA gyrases, lipid biosynthesis, cytoskeletal elements, flagella, reduced protein cost, enzymes facilitating reduced thermolabile metabolite pools) suggest that physiological adaptations are necessary to facilitate life at high temperatures, while the basic biochemistry of life is conserved.

### Metabolic Activity

In the present study, oxygen consumption rates likely would not deplete the microcosm headspace of O_2_ in the timeframe of the experiments (inferred from Murphy et al., 2013), yet, there almost certainly was an O_2_ gradient in the settled sediments of the microcosms, resulting in anaerobic habitats. Conceptually, anaerobic and aerobic respiration would be interpreted similarly with respect to substrate C atom partitioning and C flux through CCMPs. But fermentation, in addition to anabolism, is a likely contributor to the observed isotopomer mineralization ratios in the present study. In a community setting, fermentation products that are being produced faster than they are being metabolized by other community members would provide an overestimation of the contribution of anabolic reactions to experimentally determined isotopomer ratios. Despite this, C being transformed into fermentation products would not alter interpretations of organic carbon mineralization ratios, which could be used to infer C source/sink dynamics.

Substantial ^13^CO_2_ production in mercury-poisoned controls suggest that glutaraldehyde is more effective at suppressing heterotrophic activity in the GBS geothermal system (Table S11). GBS sediments consist of fine-grained particles and clay (Costa et al., 2009), potentially providing the opportunity for mercury to quickly sorb to sediment particles (Gabriel and Williamson, 2004). This may render the poison no longer bioavailable in concentrations necessary to inhibit microbial activity. This may have the effect of increasing relative rates of carbon oxidation due to increased maintenance energy demand and futile cycling from exposure to sub-lethal concentrations. Using position-specific ^13^C-labeled compounds, instead of uniformly ^13^C-labeled compounds, would allow a test of this hypothesis. Because poisoned controls containing both mercury and glutaraldehyde showed similar results to controls containing only glutaraldehyde, it is unlikely that mercury was reactive with the ^13^C-labeled compounds, suggesting high ^13^CO_2_ production rates in mercury-poisoned controls were due to biological activity. It should be noted that mercury is a better poison for inhibiting autotrophy in planktonic communities of the GBS system and little sediment is found in the water column (Boyd E. S., personal communication). We suggest that care should be taken when choosing poisons for evaluating biological activity and that combinations of poisons may be the most effective at inhibiting the biological activity of whole communities. Although glutaraldehyde was only used at the GBS 60 and GBS 95 sites, the ^13^CO_2_ analysis clearly indicated a drastic reduction in the decarboxylation of ^13^C-labeled compounds at both sites, suggesting ^13^CO_2_ from non-poisoned microcosms primarily reflected microbial activity.

Strong linear accumulation of ^13^CO_2_ was observed for all substrates at all sites (Figures 4; 5, Table S11), suggesting that communities were in a metabolic steady state (Zamboni et al., 2009). Additionally, this shows that the microbial communities at each site are adapted to the given substrates and are likely actively expressing these pathways, since carbon flux was immediate and constant for the duration of the experiment. These assumptions are supported by previous microrespirometry experiments conducted at GBS showing linear O_2_ consumption, without lag, upon the addition of organic substrates (Murphy et al., 2013).

A small amount of organic C was used in these experiments to minimize the effects of substrate additions on community metabolism (Dijkstra et al., 2011b). Yet, it has been shown that the communities in GBS sediments are electron-donor limited (Murphy et al., 2013). Thus, organic C additions may have stimulated the metabolic activity of community members and/or potentially altered the community composition over the duration of the experiment. We acknowledge that returns of ^13^C in the form of ^13^CO_2_ is likely due to the metabolism of a subset of microbes within these communities; we have shown that these pathways are intact within many, but not all, individuals of these communities (Figure 3). Attempts to use a metabolic model (Dijkstra et al., 2011b) integrating the different metabolites utilized in this study failed (data not shown). This suggests that pyruvate, glucose, and/or acetate are not being utilized by the same populations; therefore, we interpret our results in light of populations being defined by their use of a substrate (i.e., the glucose-utilizing population, pyruvate-utilizing population, or the acetate-utilizing population). We also acknowledge that several different metabolic strategies may be employed within a substrate-using population, and that we are observing the sum of these metabolic decarboxylation events. Nevertheless, the fast and steady metabolic activity and the care taken to maintain temperatures throughout the experiment provide confidence that experimental observations are ecologically relevant.

All paired isotopomer ratios showed significant differences from complete catabolic use at all sites (*p* < 0.05; one-sample *t*-test; Figures 4B–D), indicating biomass production and/or fermentation. Previous studies using position-specific ^13^C-labeled compounds are few and have focused on metabolism in aerobic soils (Dijkstra et al., 2011a,b,c, 2015; Van Groenigen et al., 2013; Hagerty et al., 2014). Except for the glucose isotopomer ratio at GBS 60, isotopomer ratios presented in this study are similar to those reported for soils. This indicates a similar retention of microbial substrate-derived carbon, relative to carbon uptake, in terrestrial geothermal settings and moderate-temperature soils. In the case of greater allocation of carbon toward anabolism and consequently a lowered relative organic C mineralization rate, a higher carbon use efficiency (CUE) (i.e., the ratio of organic C uptake allocated to biomass; mol C to biomass/ mol C uptake) is achieved. A similar CUE, despite the increased physiological stressor of high-temperature, could be a result of molecular adaptation to chronic energy stress associated with extreme environments (Valentine, 2007; Sabath et al., 2013), where high maintenance energy demands (Price and Sowers, 2004) have provided a selection pressure for efficient energy conservation. Regardless of high CUE (e.g., biomass production) or fermentation, the retention of organic carbon in the ecosystem may promote a more diverse community by providing a diversity of organic products to support diverse heterotrophs. Indeed, extreme ecosystems expected to increase maintenance energy demands (increasing organic carbon mineralization) and potentially lower CUE, such as low/high pH or high salinity, exhibit low diversity (Oren, 2001, 2011; Hou et al., 2013; Power et al., 2018).

Pyruvate isotopomer ratios suggest anabolism and/or fermentation are taking place due to ratios being significantly different from complete catabolic use (Figure 4B). Carbons from position 2 and 3 of pyruvate can be shunted from the TCA cycle for anabolic use or released in fermentation products (Figure 1). GBS 95 had a significantly higher pyruvate ratio indicating a greater proportion of pyruvate-derived carbon is sequestered in biomass and/or fermentation products than at lower temperature sites (Figure 4B). In the case of pyruvate fermentation to acetate, C_2, 3_ from pyruvate will be retained in acetate, while larger fermentation products generally result in the sequestration of all three C atoms in pyruvate (e.g., lactic acid). In the case of biomass production, a higher CUE would be expected at GBS 95 than at lower temperature sites, suggesting maintenance energy demand (i.e., catabolic carbon use) did not increase with temperature.

Acetate results are also significantly different from complete catabolic use and provide support for TCA cycle compounds being sequestered in biomass (Figure 4C). This is demonstrated by an acetate isotopomer ratio >1, suggesting the C_2_ position is being retained in biomass. In addition, acetate isotopomer ratios did not significantly differ across the sites. This indicates, again, that no change in the balance of anabolic and catabolic activities occurred, despite a nearly 35°C temperature range, suggesting a conservation of CUE and similar maintenance energy demands across temperatures.

In addition to isotopomer pairs, uniformly labeled citrate, serine, cysteine, alanine, and succinate, administered at the 85°C site only, showed at least partial catabolic utilization (Figure 5, Table S11). The use of these compounds provided additional support for a functioning TCA cycle, as the catabolism of these compounds is intimately linked to the TCA cycle and formation of pyruvate (Figure S2). These results, together with metagenomic data, provide support not just for an ecosystem-independent core functional gene assemblage for microbial communities (Sunagawa et al., 2015) but also for a conservation of function, particularly with respect to carbon flux through metabolic pathways and potential maintenance energy demands in high-temperature environments.

The 60°C site is well-below the upper-temperature limit for photosynthesis (Table 1) and several phototrophs are abundant at this site (Figure 2, green), yet incubations were carried out in the dark. This suggests this study captured the metabolic activity of a phototrophic mat during non-daylight conditions. GBS 60 showed a significant increase in the relative sequestration of C_1_ of glucose due to anabolism or fermentation, with respect to the other sites (Figure 4D). These results may be due to a reduced or inactive oxidative PPP (Dijkstra et al., 2011c, 2015). Without photosynthetic activity, anabolic reactions involving NADPH and PPP-derived biomass precursors (e.g., ribulose-5-phosphate), and the need to allocate NADPH to quenching reactive oxygen species is decreased, which would result in a low return from C_1_ of glucose. A reduced PPP is indicative of a lower demand for NADPH, which is used in many anabolic reactions directly linked to DNA replication and for scavenging of reactive oxygen species (Cox and Nelson, 2008). In addition, in the absence of light-driven primary production, a reduction in cellular replication would be expected. This is consistent with lower PPP activity due to a lower demand for the PPP-derived biomass precursor ribose-5-phosphate and NADPH for biosynthesis reactions. This interpretation fits with previous results for phototrophic mat communities in terrestrial geothermal systems that suggest that maximal rates of RNA, DNA, and protein production occur during morning hours with the lowest O_2_ concentrations found at night, reducing exposure to reactive oxygen species (Steunou et al., 2006; Kim et al., 2015).

In addition to a reduction in anabolism during dark periods, it has been shown that some members of photosynthetic mat communities inhabiting terrestrial geothermal springs switch to fermentative metabolisms during dark cycles (Anderson et al., 1987; Nold and Ward, 1996; Steunou et al., 2006; Kim et al., 2015). Furthermore, decreased PPP activity is a typical response to sub-oxic or anaerobic conditions (Godon et al., 1998; Gombert and Moreira, 2001; Le Goffe et al., 2002; Fredlund et al., 2004; Ralser et al., 2007; Williams et al., 2008; Chen et al., 2011; Dijkstra et al., 2011a). Mats in Mushroom Spring (YNP, U.S.A.) showed acetate and propionate accumulation (Kim et al., 2015), increased transcript levels for genes involved in fermentation, and decreased transcript levels for respiratory proteins at night (Steunou et al., 2006). Our observations of the catabolic use of acetate does not contradict the accumulation of acetate observed in Mushroom Spring (Kim et al., 2015), due to potential acetate production rates for some community members being greater than uptake rates of acetate-utilizing community members.

There is evidence that polyhydroxyalkanoic acid (PHA) and bacteriochlorophyll (BChl) biosynthesis is upregulated in the dark in photosynthetic mat communities, resulting in the majority of C from glucose being retained in biomass (Klatt et al., 2013). Under this scenario, with reduced PPP activity, C flux through biosynthetic pathways would show a strong ^13^CO_2_ signal from C_3_ and C_4_ of glucose (Dijkstra et al., 2015), from the formation of acetyl-CoA from glycolysis-derived pyruvate, providing a strong ^13^CO_2_ return from uniformly labeled glucose and a low return from C_1_ of glucose. The increased anabolism linked to PHA and BChl at night (Klatt et al., 2013) does not necessarily conflict with an overall reduction in anabolism (Kim et al., 2015). PHAs can be used as storage molecules that can be degraded for mixotrophic use in the light. It has been suggested that BChl biosynthesis in these communities is inhibited by O_2_ (Klatt et al., 2013). Taken together, the glucose-utilizing populations in GBS 60 may be replicating during the day (requiring total biomass replication and high PPP metabolite and NADPH demand) and building storage molecules and photosynthetic apparatus at night with minimal PPP activity (PHA formation and BChl-specific biomass precursor demand), resulting in greater retention of C_1_ of glucose, with respect to C_1_ oxidation in the PPP (Dijkstra et al., 2015). Future studies may achieve greater resolution of C flux through metabolic pathways from the use of a greater diversity of position-specific arrangements (Leighty and Antoniewicz, 2013; Dijkstra et al., 2015) and evaluating carbon flux during diel cycles.

In contrast to the glucose C_U_: C_1_ results at 60°C, higher temperature sites exhibited a decreased ratio, with GBS 95 being significantly lower than GBS 60 and GBS 70 (Figure 4D). This is assumed to be the result of ^13^CO_2_ being produced from the C_1_ position in the PPP (Figure 1) and/or reduced fermentation. This may indicate the production of nucleotides and NADPH, both indicative of anabolism directly related to cellular replication, in contrast to the potential sequestration of C_1_ from anabolism of PAHs and BChl suggested at the 60°C site. On the other hand, an increase in glycolytic C flux through the Entner–Doudoroff (ED) pathway would result in an increase in C_1_ oxidation during acetate formation. This is due to glucose conversion to pyruvate resulting in one molecule of pyruvate retaining the C_1_ of glucose in the C_1_ position of pyruvate, and subsequent pyruvate oxidation in the synthesis of acetyl-coA (i.e., ^13^CO_2_ production from C_1_ of pyruvate). In fact, *Archaea* are known to have incomplete PPP pathways, which is shown in representative MAGs, while many utilize modified versions of the ED pathway (Bräsen et al., 2014). Populated KEGG maps and MCRs produced in this study contrast with respect to the completeness of the ED pathway. While both confirm the typical ED pathway is only complete in the GBS 70 metagenome, MCR analysis suggests that the non-phosphorylative ED pathway is biologically feasible in all metagenomes and that the semi-phosphorylative ED pathway is not (Table S5). In contrast, populated KEGG maps (Kanehisa and Goto, 2000; Kanehisa et al., 2017, 2012) suggest that the non-phosphorylative ED pathway is not possible in GBS 85, due to the absence of EC 4.1.2.55 (K11395), which has a low relative abundance in GBS 70, while the semi-phosphorylative ED pathway appears possible (Figure S2, Table S6). The ED pathways requires seven-fold less enzymatic protein than glycolysis, the use of which may be a result of environmental selection pressures for reduced protein load at the cost of ATP gain (1 vs. 2 ATP, with respect to glycolysis) as a consequence of increased maintenance energy costs associated with protein repair and turnover at high temperatures (Price and Sowers, 2004; Flamholz et al., 2013).

Future studies should strive to obtain net CO_2_ production rates and ^13^C label consumption rates to allow extrapolations to absolute rates of C flux, community respiration rates, and calculations of carbon mass balance. Additional studies would benefit from pairing with additional “omics” techniques (i.e., transcriptomics and proteomics) and organism-level stable isotope techniques [e.g., quantitative stable isotope probing Hungate et al., 2015; Morrissey et al., 2016 FISH-NanoSIMS Dekas and Orphan, 2010; Musat et al., 2012, 2016]. As more microbial communities are probed by isotopomer ratio studies, cross-community comparisons can be made to better inform relationships between diversity and microbially-mediated C biogeochemistry and maintenance energy demands (CUE), and progress can be made toward a quantitative understanding of microbial ecosystems.

In summary, this study demonstrated that, despite domain-level shifts in community composition and a decrease in diversity with increasing temperature, CCMP genes and biochemical efficiency were conserved across a broad temperature gradient in GBS. Evidence for a stable or decreased maintenance energy demand with increasing habitat temperature was found: pyruvate and acetate utilization across all sites, and glucose from 70 to 95°C, suggest a high CUE and retention of organic carbon at all temperatures. In contrast, significant deviations in C flux through CCMPs with respect to glucose were observed in the presence of phototrophs.

## Data Availability

The datasets generated for this study can be found in NCBI Sequence Read Archive; Integrated Microbial Genomes and Microbiomes platform, SRP059341, SRX1055762, SRX1055763, SRX1055764, and SRX1055765; Ga0197142, Ga0197143, and Ga0197144.

## Author Contributions

ST, BH, KT, and PD contributed to experimental design. ST and KT completed field work. ST, KT, and JD completed laboratory work. ST, JD, CS, SM, and DL contributed to bioinformatics work. EE-F oversaw metagenomic sequencing, assembly, and MAG generation. ST analyzed the data and wrote the manuscript with input from all authors.

### Conflict of Interest Statement

The authors declare that the research was conducted in the absence of any commercial or financial relationships that could be construed as a potential conflict of interest.
