# Supplementary material for: Position-Specific Metabolic Probing and Metagenomics of Microbial Communities Reveal Conserved Central Carbon Metabolic Network Activities at High Temperatures
Source: Front Microbiol. 2019 Jul 5;10:1427. doi: 10.3389/fmicb.2019.01427 (PMC6624737; doi:10.3389/fmicb.2019.01427)
Supplement: Supplementary file 14 [file Data_Sheet_1.PDF]

Supplementary Figure S1: Detailed site description and experimental design.

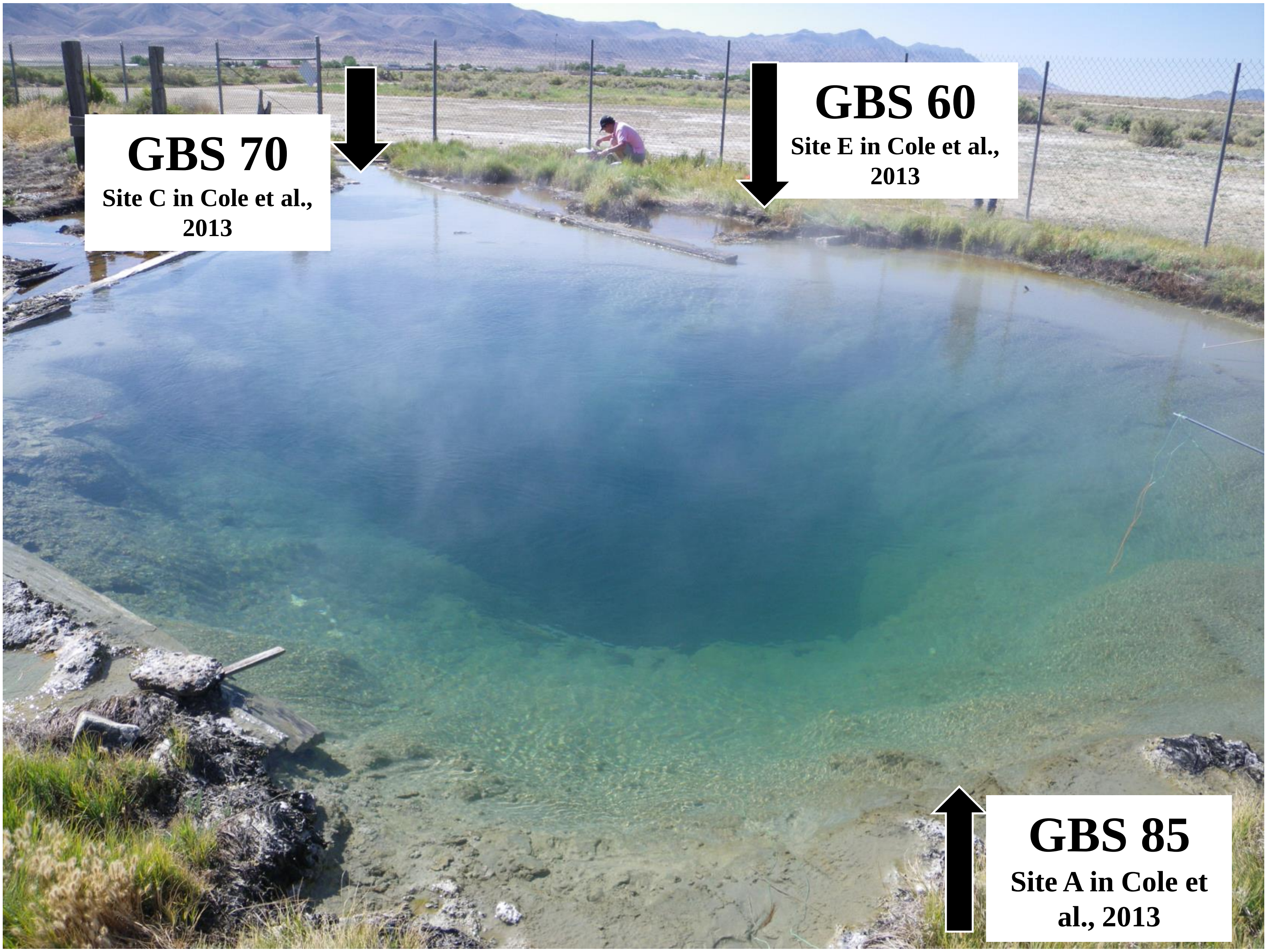

Great Boiling Spring (GBS), showing three sampling locations used in this study. See Table 1 in the text for temperature and pH, see Table S1 for GPS location. For other studies concerning GBS see Cole et al., 2013; Costa et al 2009; Murphy et al., 2013; Dodsworth et al., 2012; Zhang et al., 2013; Peacock et al., 2013; Paraiso et al., 2013. Picture of GBS is modified from Paraiso et al., 2013 and was not taken during the current study.

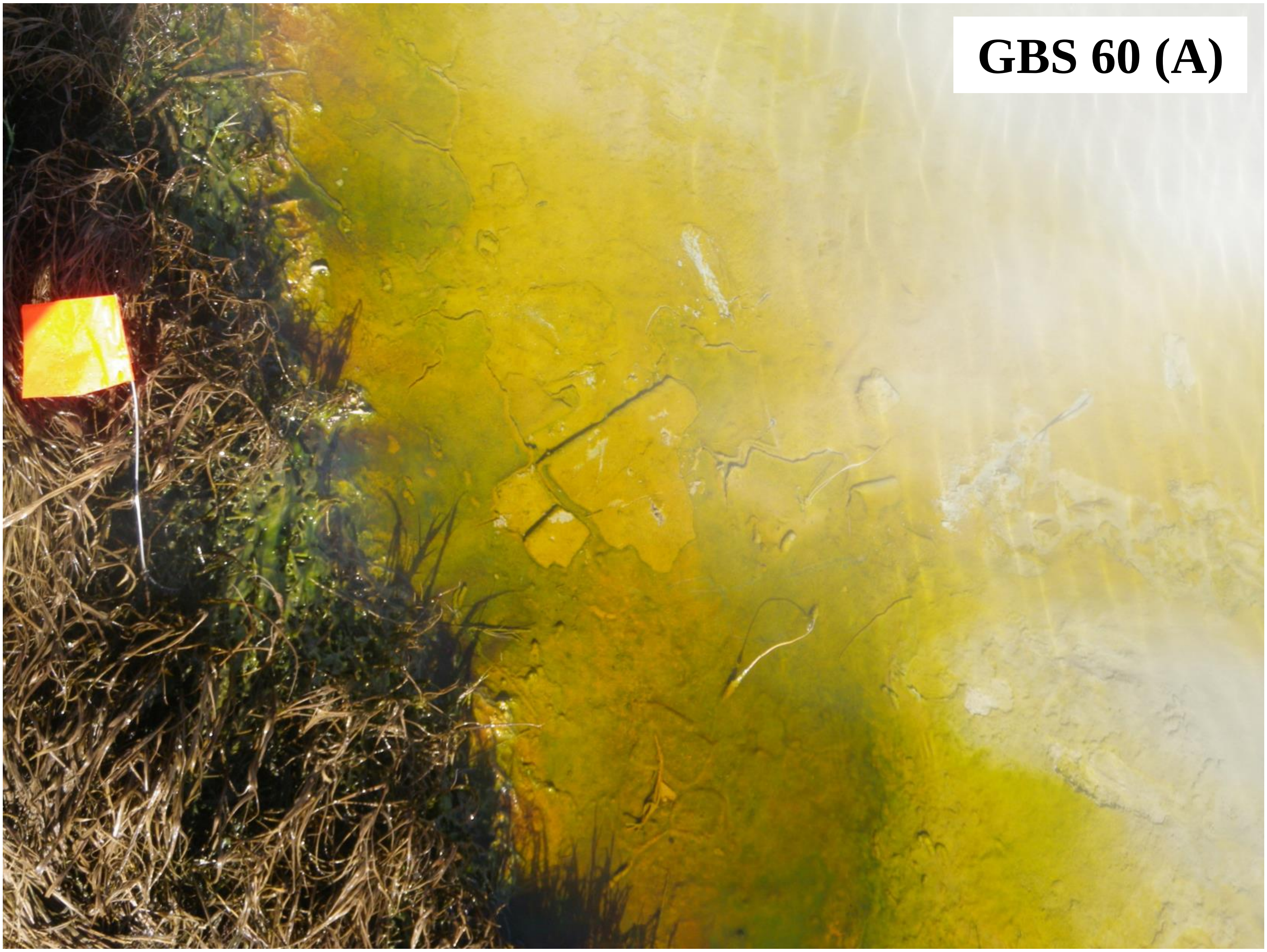

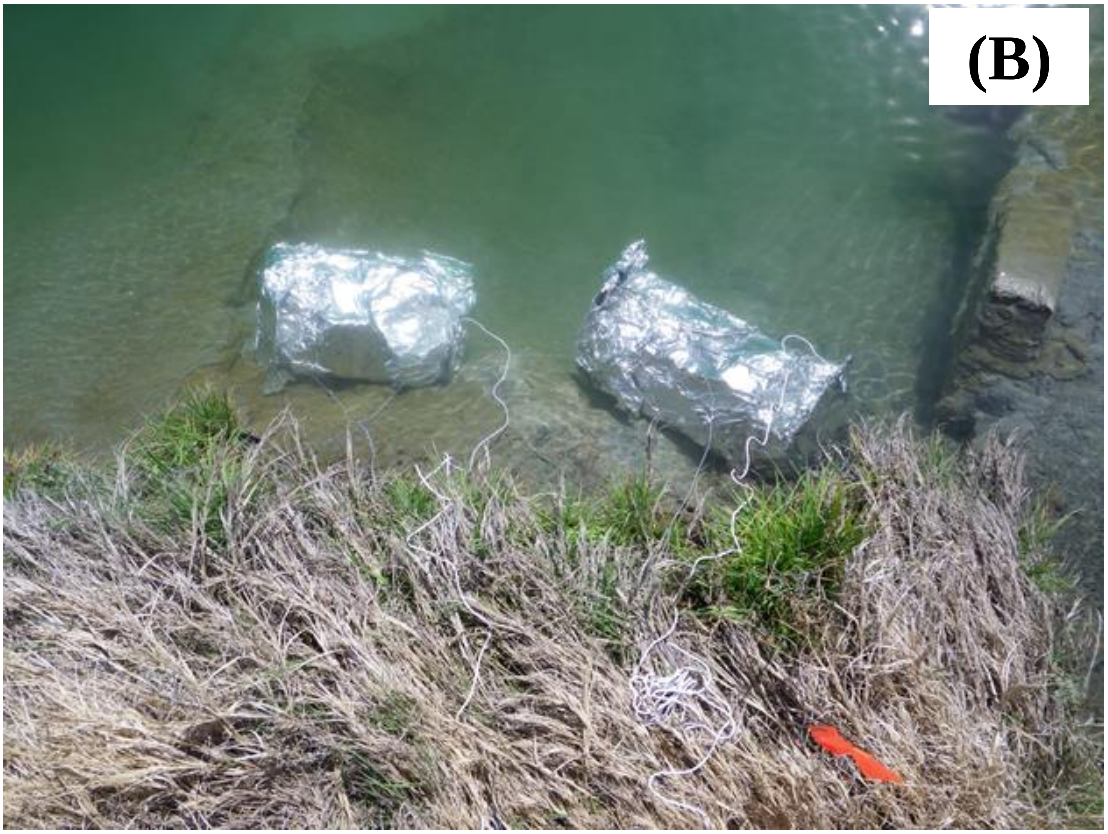

GBS 60 sampling site (A) and incubation site (B). Pigmented microbial mats can be seen covering a fine tan sediment (A; marker flag for scale). Incubations took place in a deeper section of an adjacent spring in order to fully submerge the samples. Temperature loggers can be seen coming from aluminum foil wrapped wire cages housing the incubation bottles (B).

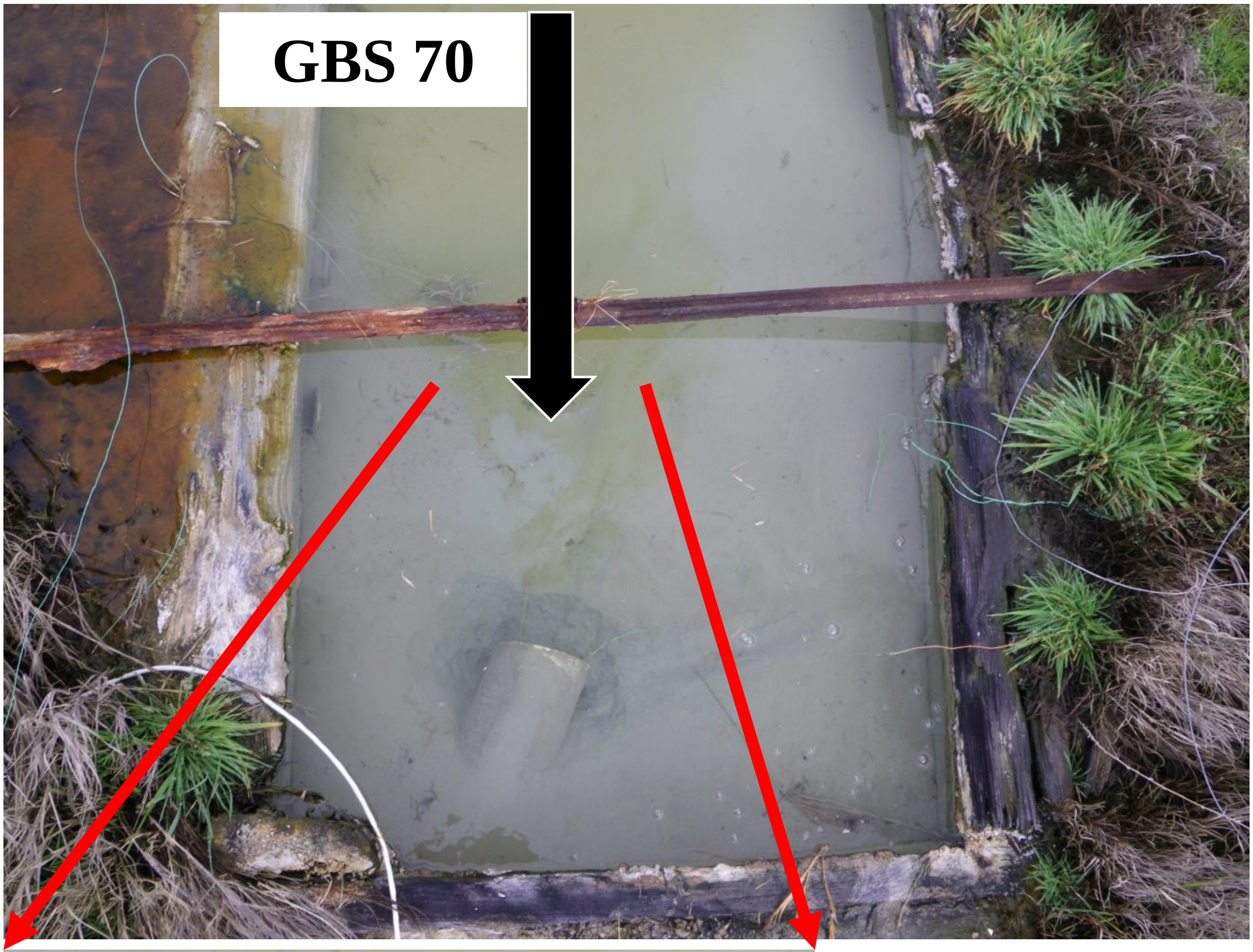

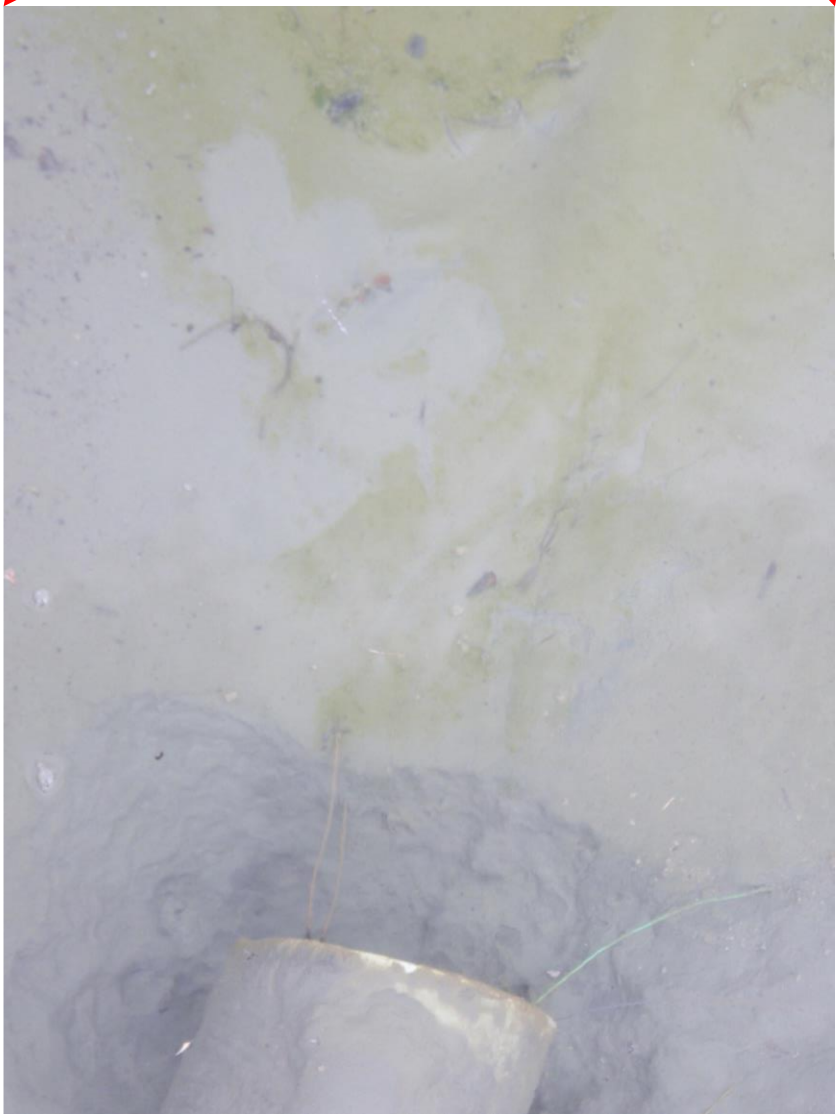

GBS 70 sampling site with close up of sediment. Sediment was very homogenous, light tan/white in color with some faint green, very fluffy, and flock-like. Some of the flow from GBS is directed through the pipe at the bottom center of each picture. There was a noticeable flow through the sample site.

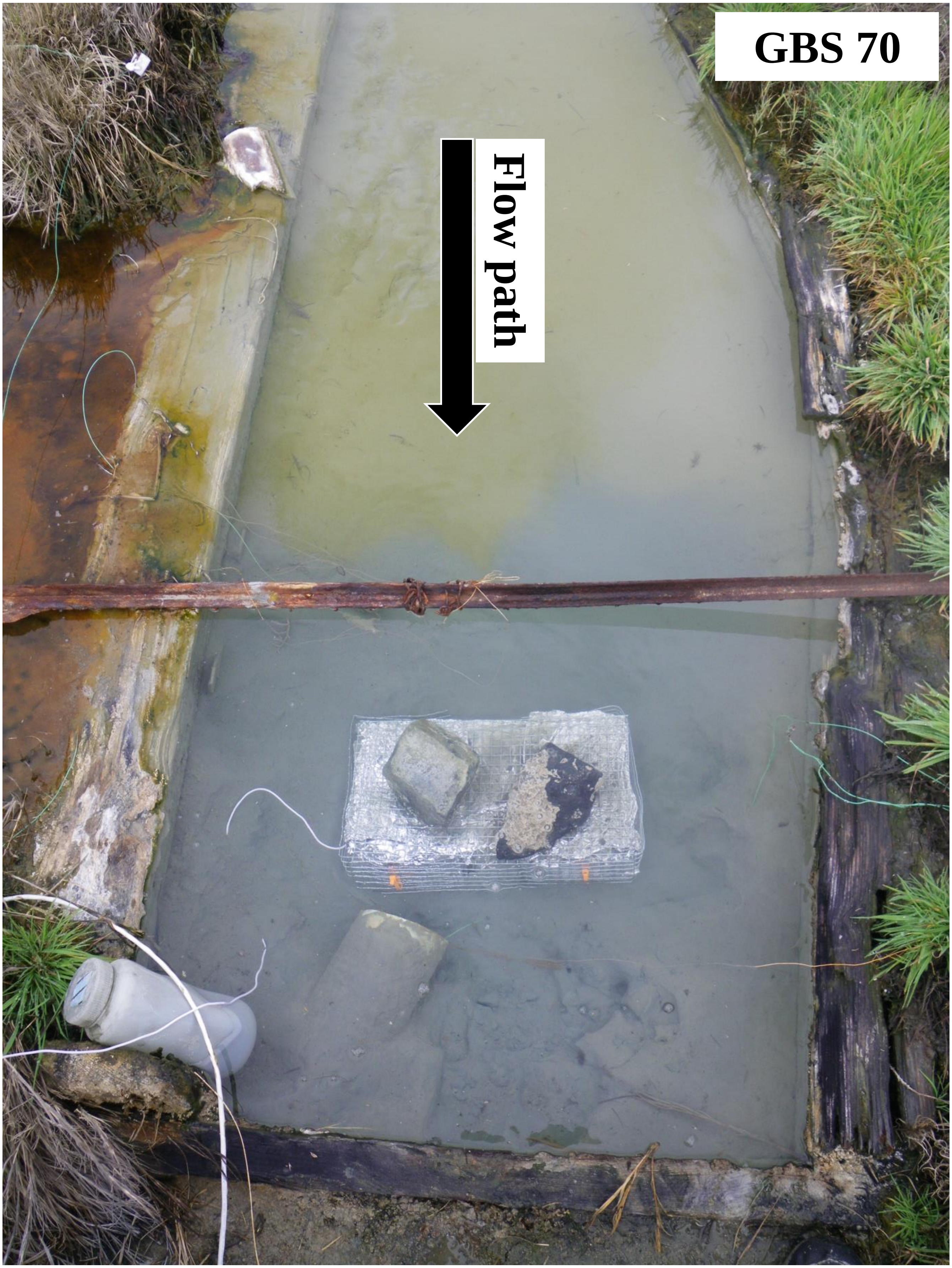

Flow path and incubation cage for GBS 70.

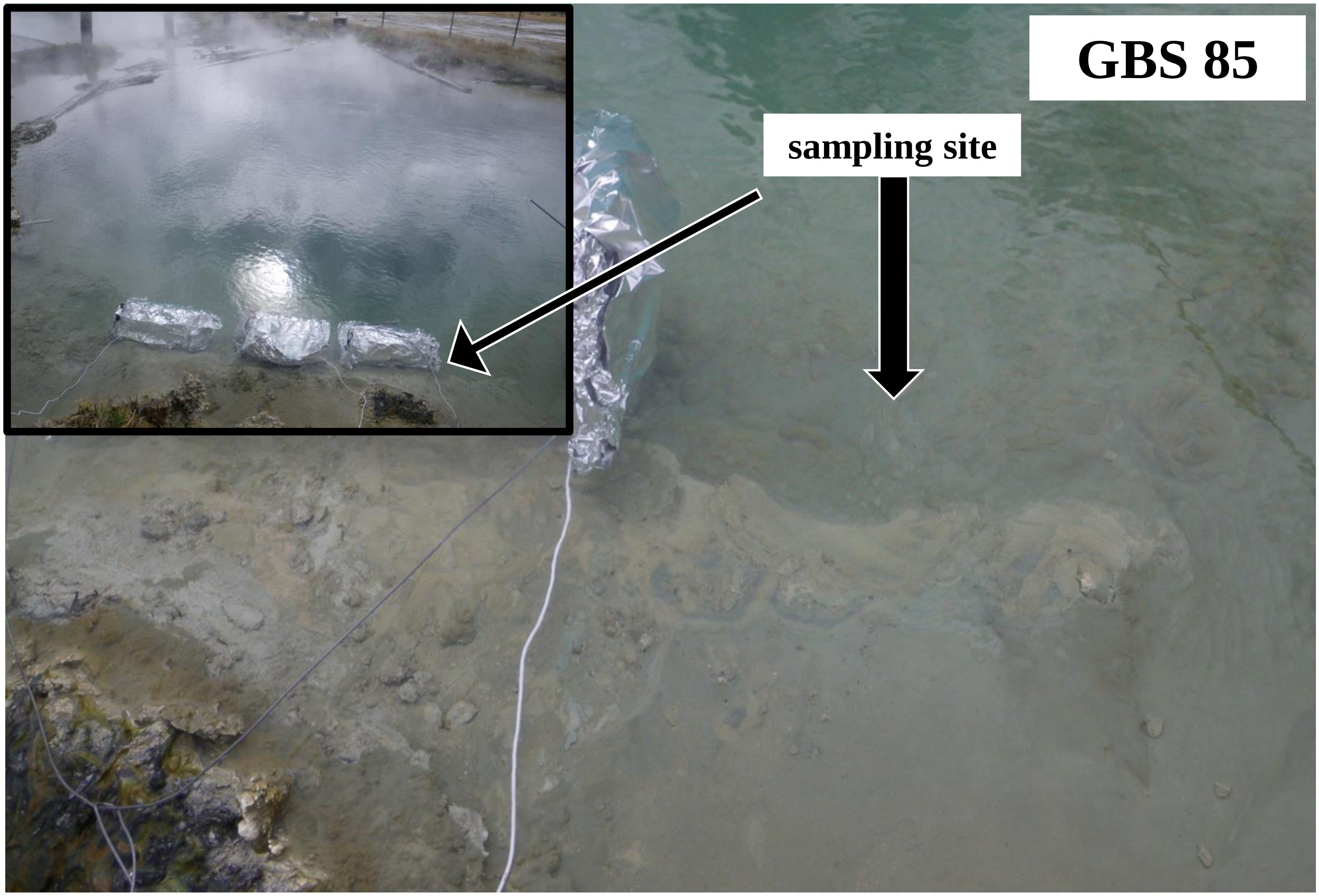

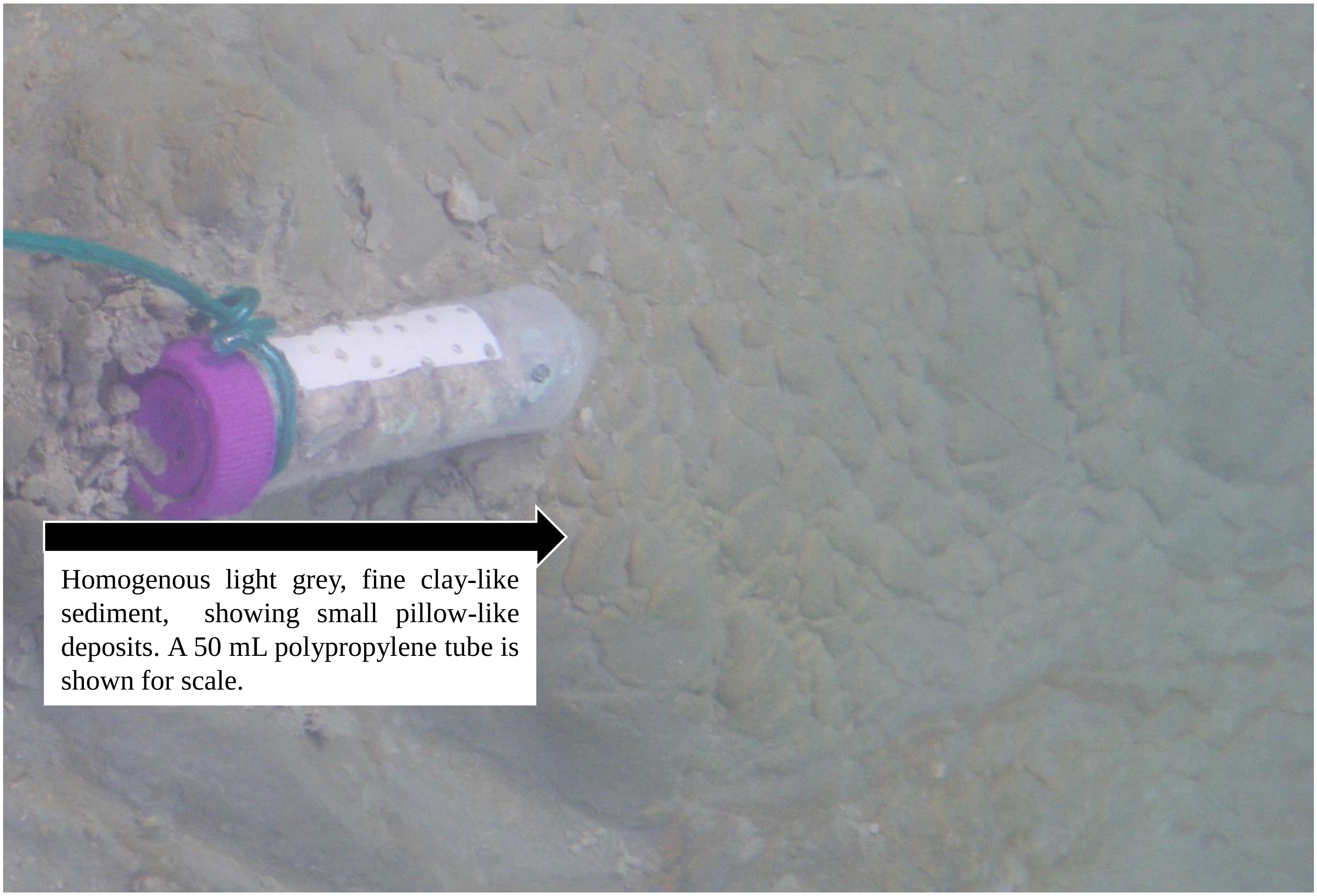

Homogenous light grey, fine clay-like sediment, showing small pillow-like deposits. A 50 mL polypropylene tube is shown for scale.

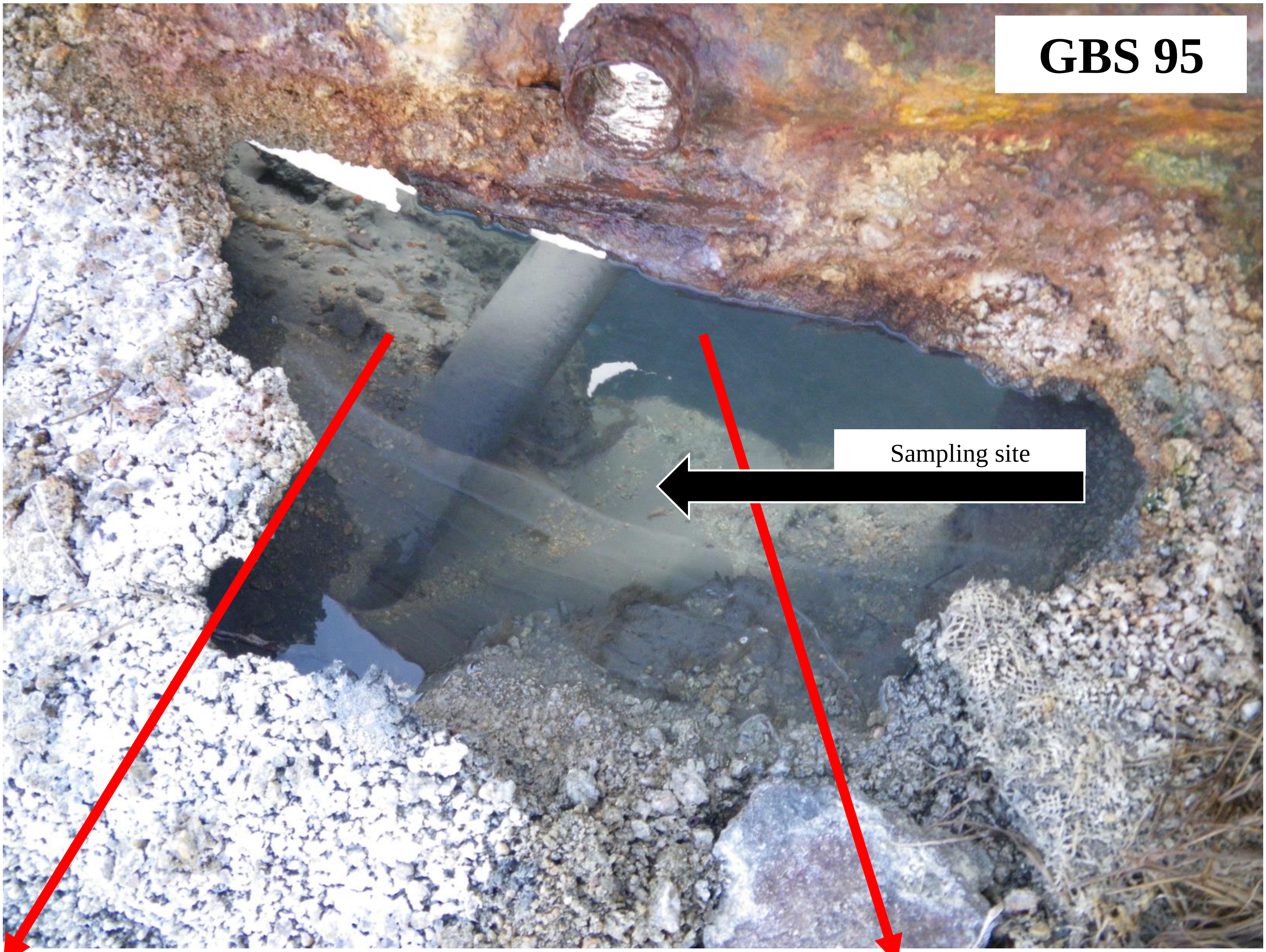

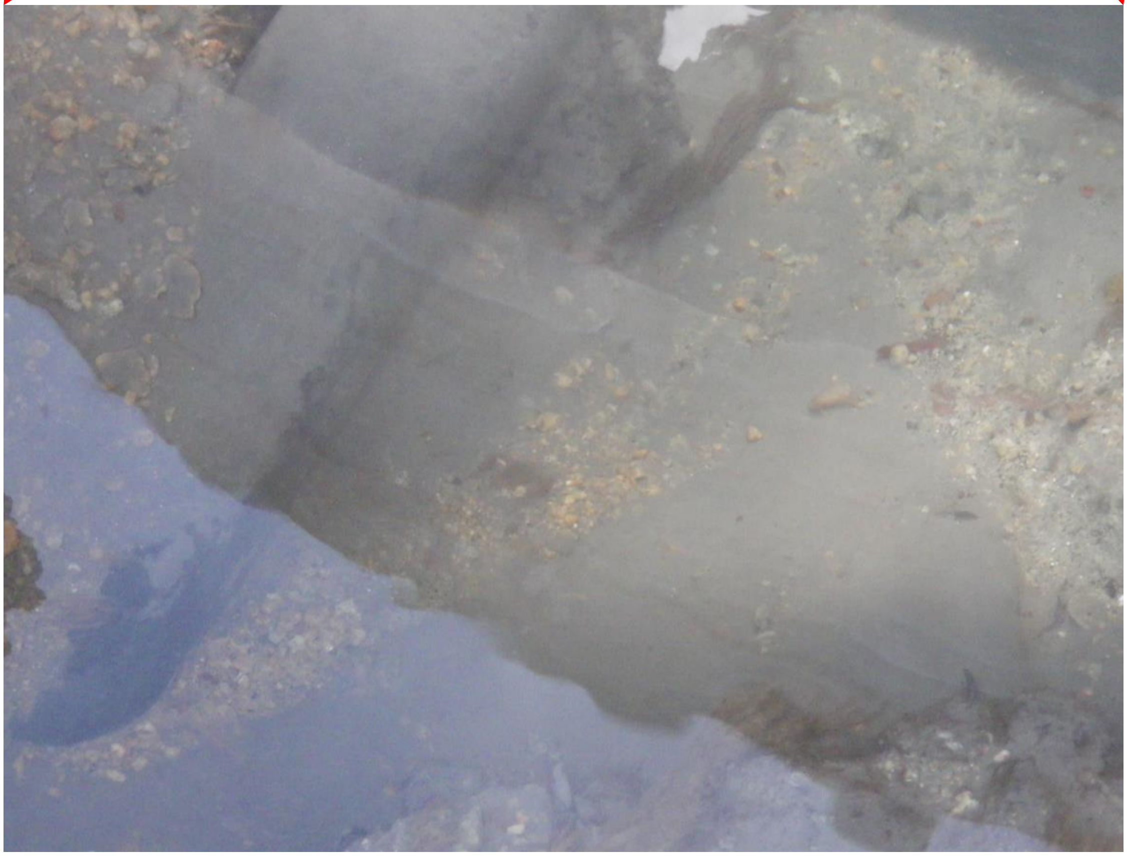

GBS 95 (aka GBS 19) is separate from Great Boiling Spring and was surrounded by a large metal culvert. A PVC pipe is shown which allows partial drainage. Sediment was dark black/grey with small rocks and dead root-like material. The main pool of GBS 95 was boiling vigorously during sampling and sample incubation. Samples were fully submerged and incubated on the inside of the culvert.

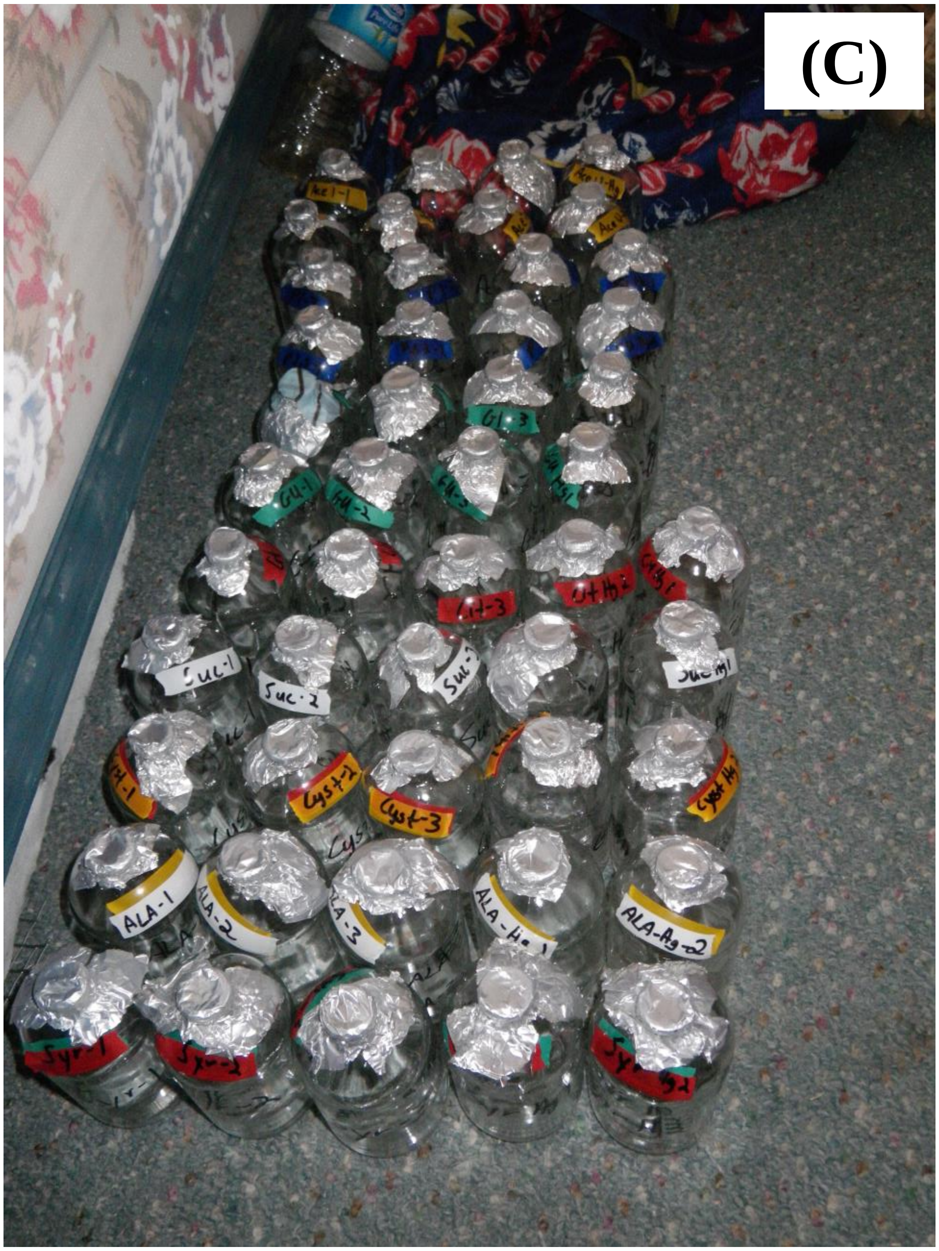

An example of incubation bottles prior to use (C).

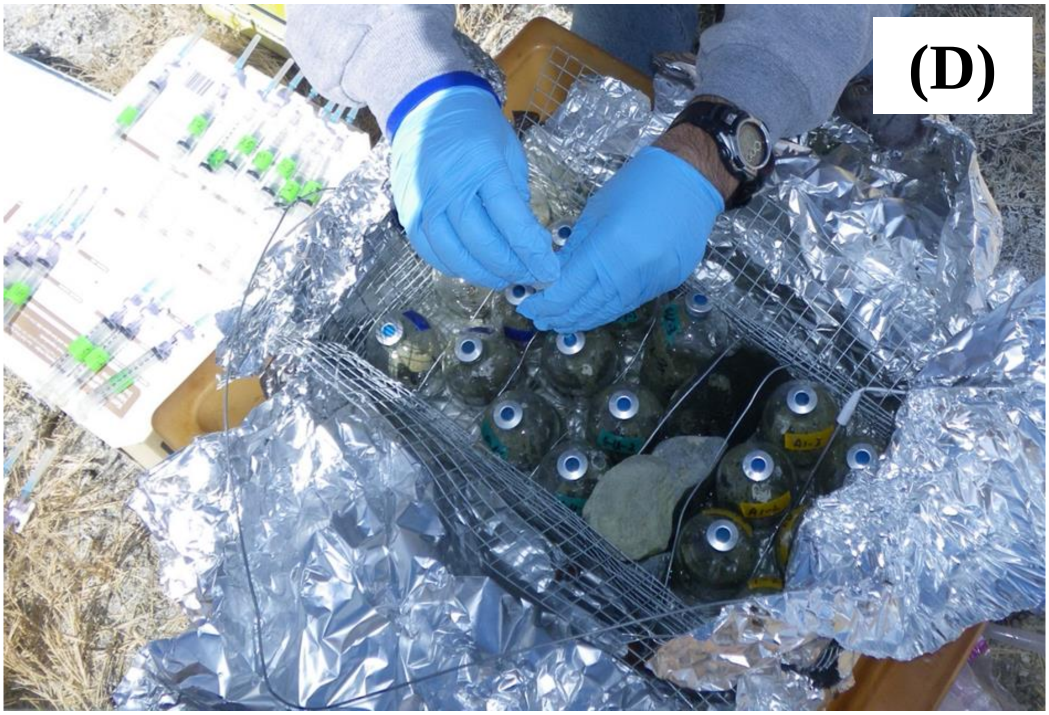

An example of headspace sampling and the design of the wire cages. Rocks were used to weigh cages down and keep them fully submerged (D).
