# Supplementary material for: Position-Specific Metabolic Probing and Metagenomics of Microbial Communities Reveal Conserved Central Carbon Metabolic Network Activities at High Temperatures
Source: Front Microbiol. 2019 Jul 5;10:1427. doi: 10.3389/fmicb.2019.01427 (PMC6624737; doi:10.3389/fmicb.2019.01427)
Supplement: Supplementary file 15 [file Data_Sheet_2.PDF]

# Supplementary Figure S2: Venn diagrams and populated KEGG maps

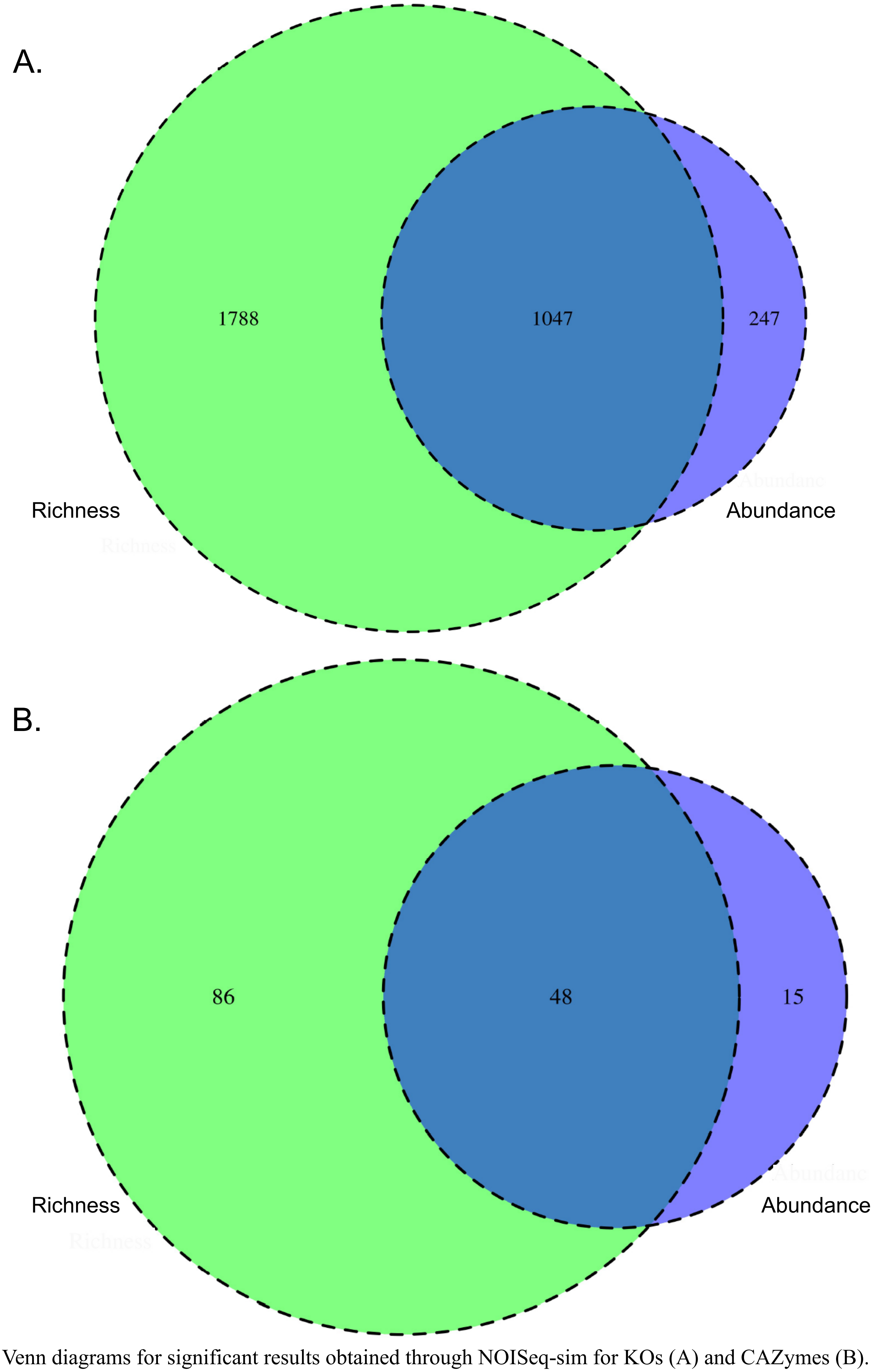


Venn diagrams for significant results obtained through NOISeq-sim for KOs (A) and CAZymes (B). See table S10 for complete lists.

# KO abundance- and richness-populated KEGG maps.

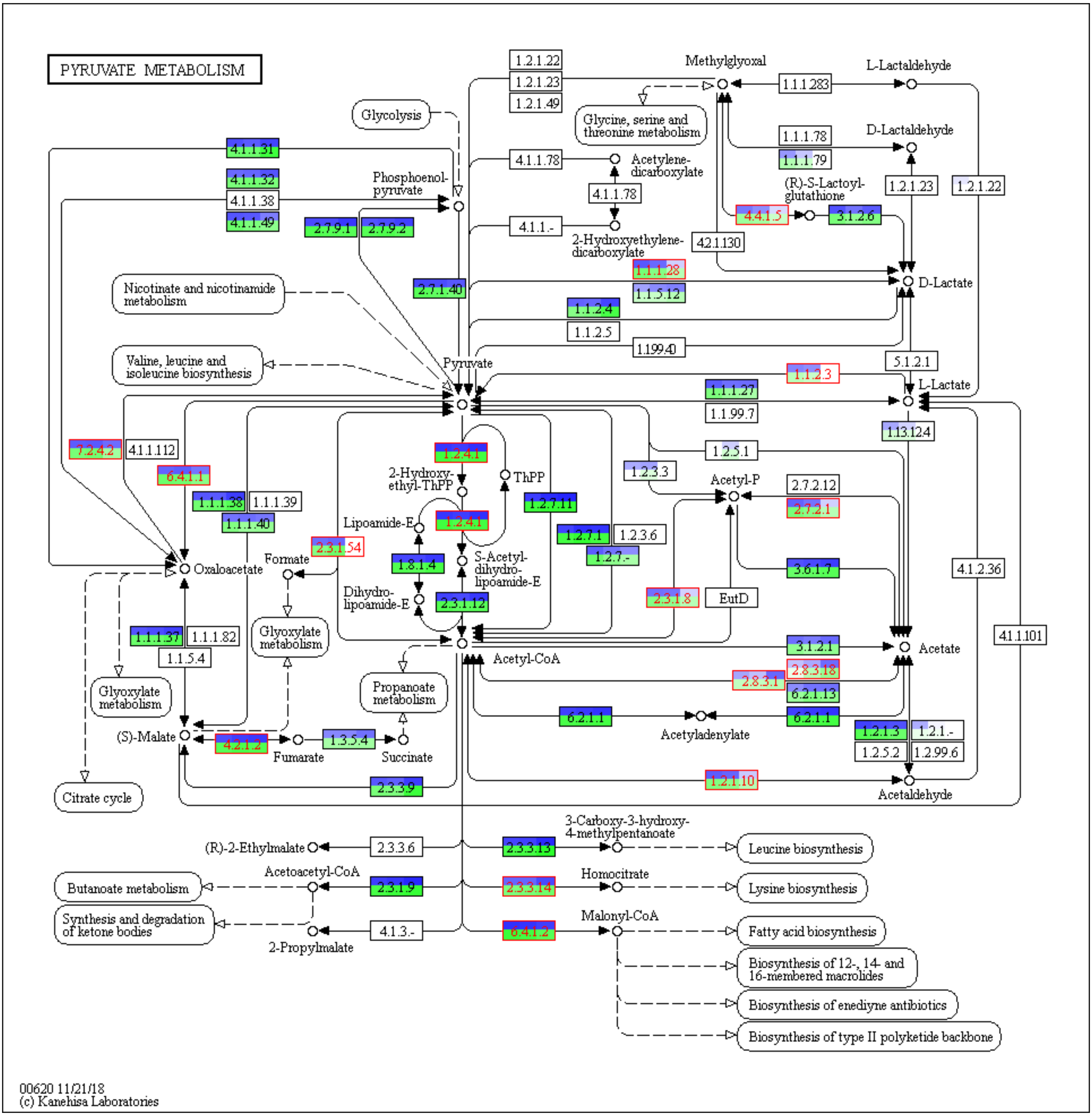


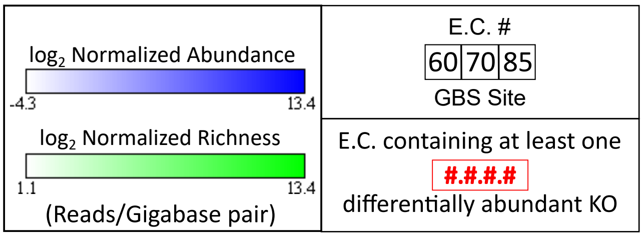

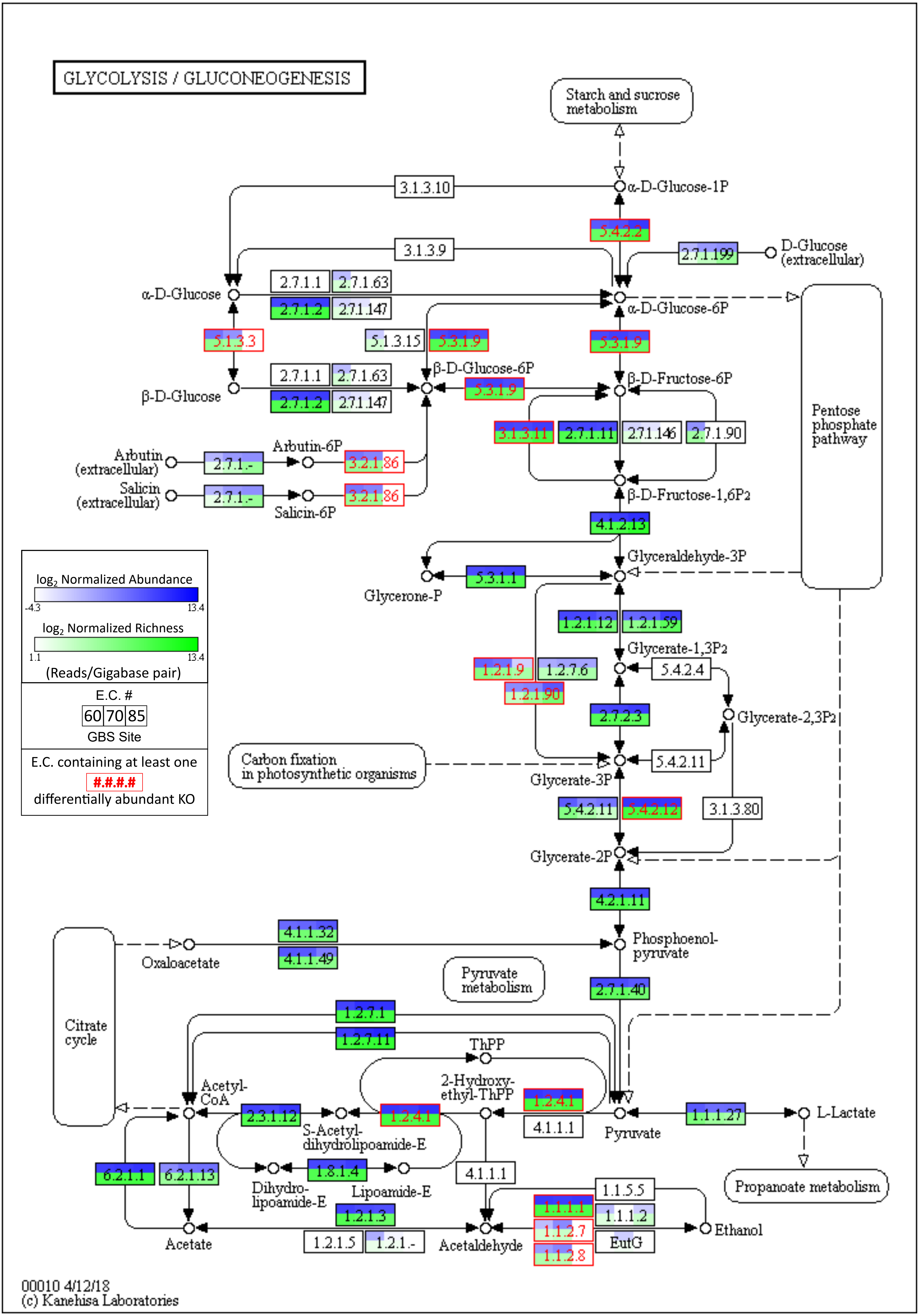
GLYCOLYSIS / GLUCONEOGENESIS
Starch and sucrose metabolism
3.1.3.10
α-D-Glucose-1P
5.4.2.2
3.1.3.9
2.7.1.199
D-Glucose (extracellular)
α-D-Glucose
2.7.1.1
2.7.1.63
2.7.1.2
2.7.1.147
α-D-Glucose-6P
5.1.3.3
5.1.3.15
5.3.1.9
β-D-Glucose-6P
β-D-Fructose-6P
β-D-Glucose
Pentose phosphate pathway
3.1.3.11
2.7.1.11
2.7.1.146
2.7.1.90
Arbutin (extracellular)
2.7.1.-
Arbutin-6P
3.2.1.86
Salicin (extracellular)
Salicin-6P
β-D-Fructose-1,6P2
4.1.2.13
Glyceraldehyde-3P
5.3.1.1
Glycerone-P
log2 Normalized Abundance
-4.3
13.4
log2 Normalized Richness
1.1
13.4
(Reads/Gigabase pair)
E.C. #
60
70
85
GBS Site
E.C. containing at least one
#.#.#.#
differentially abundant KO
1.2.1.12
1.2.1.59
Glycerate-1,3P2
1.2.1.9
1.2.7.6
1.2.1.90
5.4.2.4
2.7.2.3
Glycerate-2,3P2
Carbon fixation in photosynthetic organisms
5.4.2.11
Glycerate-3P
5.4.2.11
5.4.2.12
3.1.3.80
Glycerate-2P
4.2.1.11
Citrate cycle
Oxaloacetate
4.1.1.32
4.1.1.49
Phosphoenol-pyruvate
Pyruvate metabolism
2.7.1.40
1.2.7.1
1.2.7.11
ThPP
2-Hydroxy-ethyl-ThPP
Acetyl-CoA
2.3.1.12
S-Acetyl-dihydrolipoamide-E
1.2.4.1
1.2.4.1
4.1.1.1
Pyruvate
1.1.1.27
L-Lactate
6.2.1.1
6.2.1.13
Dihydro-lipoamide-E
1.8.1.4
Lipoamide-E
4.1.1.1
1.1.5.5
Propanoate metabolism
1.2.1.3
1.1.1.1
1.1.1.2
1.1.2.7
Ethanol
Acetate
1.2.1.5
1.2.1.-
Acetaldehyde
EutG
1.1.2.8
00010 4/12/18
(c) Kanehisa Laboratories

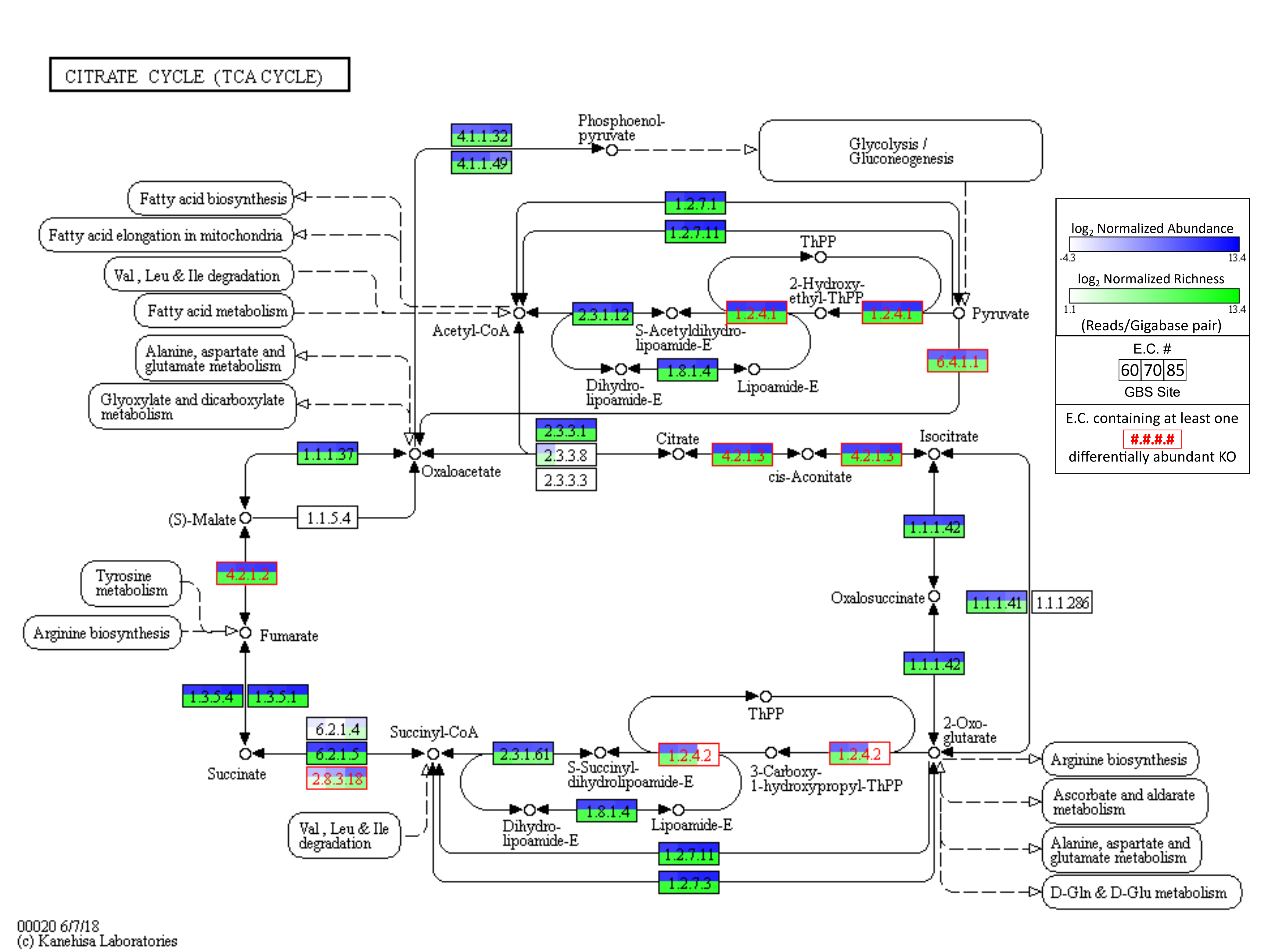
CITRATE CYCLE (TCA CYCLE)
Phosphoenol-pyruvate
Glycolysis / Gluconeogenesis
4.1.1.32
4.1.1.49
Fatty acid biosynthesis
Fatty acid elongation in mitochondria
Val, Leu & Ile degradation
Fatty acid metabolism
Alanine, aspartate and glutamate metabolism
Glyoxylate and dicarboxylate metabolism
1.2.7.1
1.2.7.11
ThPP
2-Hydroxy-ethyl-ThPP
Acetyl-CoA
2.3.1.12
S-Acetyldihydro-lipoamide-E
1.2.4.1
1.2.4.1
Pyruvate
6.4.1.1
1.8.1.4
Dihydro-lipoamide-E
Lipoamide-E
2.3.3.1
2.3.3.8
2.3.3.3
Citrate
4.2.1.3
4.2.1.3
cis-Aconitate
Isocitrate
1.1.1.37
Oxaloacetate
(S)-Malate
1.1.5.4
4.2.1.2
Tyrosine metabolism
Arginine biosynthesis
Fumarate
1.1.1.42
Oxalosuccinate
1.1.1.41
1.1.1.286
1.1.1.42
1.3.5.4
1.3.5.1
6.2.1.4
6.2.1.5
2.8.3.18
Succinate
Succinyl-CoA
Val, Leu & Ile degradation
2.3.1.61
S-Succinyl-dihydrolipoamide-E
1.2.4.2
1.2.4.2
ThPP
3-Carboxy-1-hydroxypropyl-ThPP
2-Oxo-glutarate
1.8.1.4
Dihydro-lipoamide-E
Lipoamide-E
1.2.7.11
1.2.7.3
Arginine biosynthesis
Ascorbate and aldarate metabolism
Alanine, aspartate and glutamate metabolism
D-Gln & D-Glu metabolism
log2 Normalized Abundance
-4.3
13.4
log2 Normalized Richness
1.1
13.4
(Reads/Gigabase pair)
E.C. #
60
70
85
GBS Site
E.C. containing at least one
#.#.#.#
differentially abundant KO
00020 6/7/18
(c) Kanehisa Laboratories

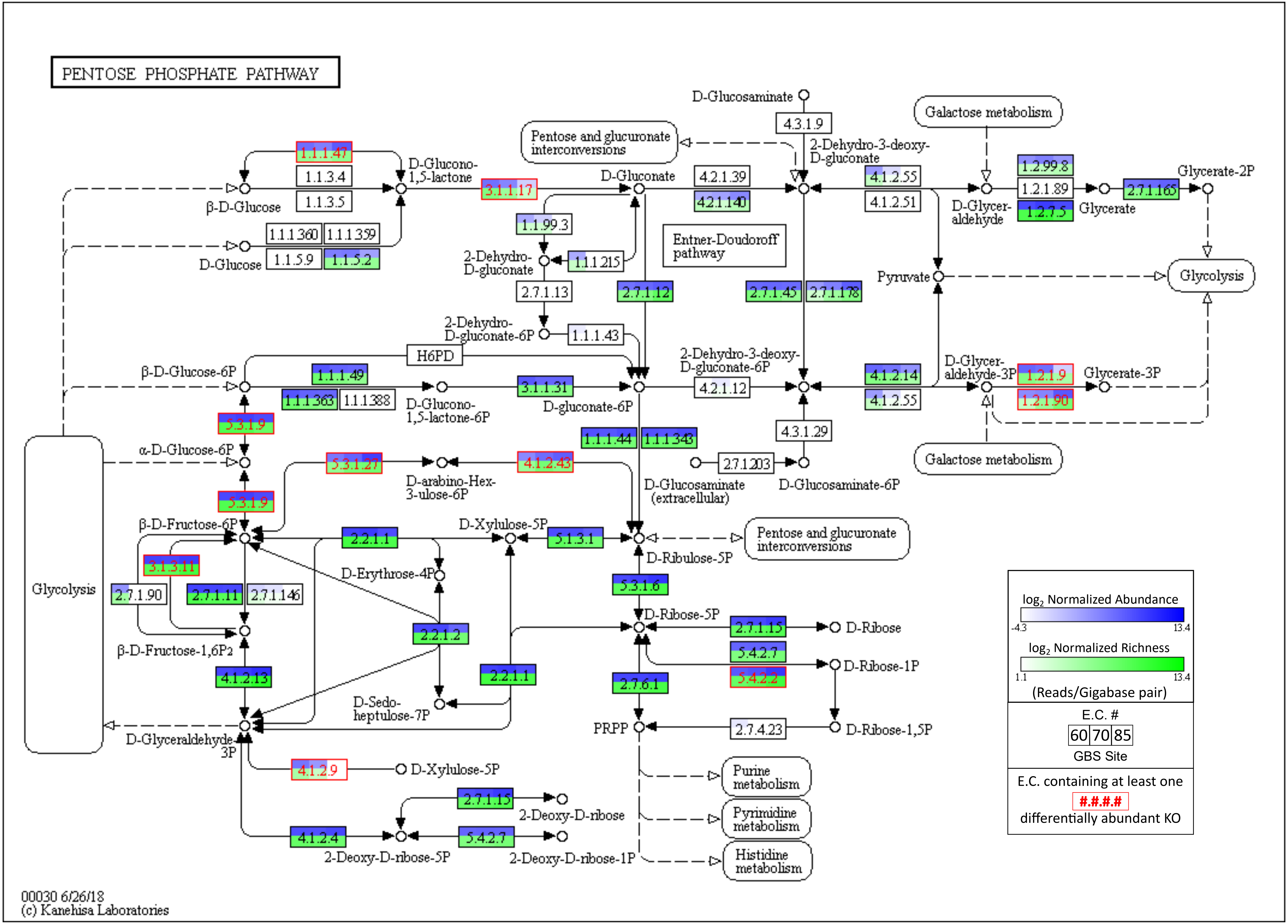
PENTOSE PHOSPHATE PATHWAY
D-Glucosaminate
4.3.1.9
Galactose metabolism
Pentose and glucuronate interconversions
2-Dehydro-3-deoxy-D-gluconate
1.1.1.47
1.1.3.4
1.1.3.5
D-Glucono-1,5-lactone
3.1.1.17
D-Gluconate
4.2.1.39
4.2.1.140
4.1.2.55
4.1.2.51
1.2.99.8
1.2.1.89
1.2.7.5
2.7.1.165
Glycerate-2P
Glycerate
D-Glycer-aldehyde
β-D-Glucose
1.1.1.360
1.1.1.359
1.1.5.9
1.1.5.2
D-Glucose
1.1.99.3
2-Dehydro-D-gluconate
1.1.1.215
Entner-Doudoroff pathway
Pyruvate
Glycolysis
2.7.1.13
2.7.1.12
2.7.1.45
2.7.1.178
2-Dehydro-D-gluconate-6P
1.1.1.43
H6PD
β-D-Glucose-6P
1.1.1.49
1.1.1.363
1.1.1.388
D-Glucono-1,5-lactone-6P
3.1.1.31
D-gluconate-6P
2-Dehydro-3-deoxy-D-gluconate-6P
4.2.1.12
4.1.2.14
4.1.2.55
D-Glycer-aldehyde-3P
1.2.1.9
1.2.1.90
Glycerate-3P
5.3.1.9
α-D-Glucose-6P
5.3.1.27
D-arabino-Hex-3-ulose-6P
4.1.2.43
1.1.1.44
1.1.1.343
4.3.1.29
2.7.1.203
D-Glucosaminate (extracellular)
D-Glucosaminate-6P
Galactose metabolism
Glycolysis
5.3.1.9
β-D-Fructose-6P
2.2.1.1
D-Xylulose-5P
5.1.3.1
D-Ribulose-5P
Pentose and glucuronate interconversions
3.1.3.11
2.7.1.90
2.7.1.11
2.7.1.146
D-Erythrose-4P
5.3.1.6
D-Ribose-5P
2.7.1.15
D-Ribose
5.4.2.7
5.4.2.2
D-Ribose-1P
β-D-Fructose-1,6P2
2.2.1.2
4.1.2.13
2.2.1.1
2.7.6.1
D-Sedo-heptulose-7P
PRPP
2.7.4.23
D-Ribose-1,5P
D-Glyceraldehyde-3P
4.1.2.9
D-Xylulose-5P
Purine metabolism
2.7.1.15
2-Deoxy-D-ribose
Pyrimidine metabolism
4.1.2.4
5.4.2.7
2-Deoxy-D-ribose-5P
2-Deoxy-D-ribose-1P
Histidine metabolism
log2 Normalized Abundance
-4.3
13.4
log2 Normalized Richness
1.1
13.4
(Reads/Gigabase pair)
E.C. #
60
70
85
GBS Site
E.C. containing at least one
#.#.#.#
differentially abundant KO
00030 6/26/18
(c) Kanehisa Laboratories

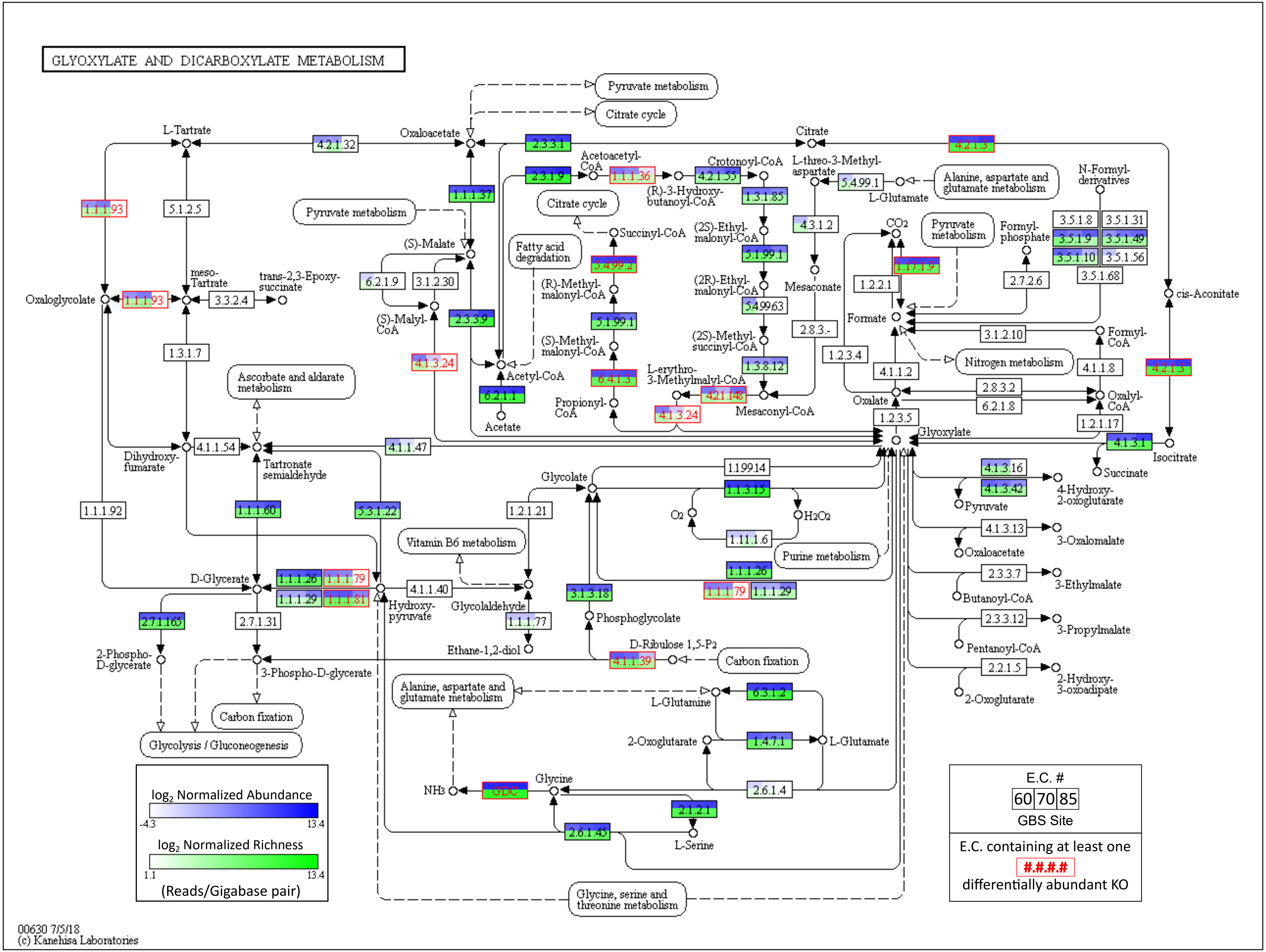

GLYOXYLATE AND DICARBOXYLATE METABOLISM
Pyruvate metabolism
Citrate cycle
L-Tartrate
4.2.1.32
Oxaloacetate
2.3.3.1
Citrate
4.2.1.3
1.1.1.93
5.1.2.5
Acetoacetyl-CoA
2.3.1.9
1.1.1.36
Crotonoyl-CoA
4.2.1.55
L-threo-3-Methyl-aspartate
5.4.99.1
L-Glutamate
Alanine, aspartate and glutamate metabolism
N-Formyl-derivatives
1.1.1.37
(R)-3-Hydroxy-butanoyl-CoA
1.3.1.85
Citrate cycle
(S)-Malate
Fatty acid degradation
Succinyl-CoA
(2S)-Ethyl-malonyl-CoA
4.3.1.2
CO2
Formyl-phosphate
3.5.1.8
3.5.1.31
3.5.1.9
3.5.1.49
3.5.1.10
3.5.1.56
meso-Tartrate
trans-2,3-Epoxy-succinate
6.2.1.9
3.1.2.30
5.4.99.2
5.1.99.1
1.17.1.9
(R)-Methyl-malonyl-CoA
(2R)-Ethyl-malonyl-CoA
Mesaconate
1.2.2.1
2.7.2.6
3.5.1.68
cis-Aconitate
Oxaloglycolate
3.3.2.4
(S)-Malyl-CoA
2.3.3.9
5.4.99.63
Formate
(S)-Methyl-malonyl-CoA
(2S)-Methyl-succinyl-CoA
2.8.3.-
3.1.2.10
Formyl-CoA
1.3.1.7
4.1.3.24
1.3.8.12
1.2.3.4
4.1.1.2
Nitrogen metabolism
4.1.1.8
Ascorbate and aldarate metabolism
Acetyl-CoA
6.4.1.3
L-erythro-3-Methylmalyl-CoA
4.2.1.148
2.8.3.2
Oxalyl-CoA
6.2.1.1
Propionyl-CoA
Mesaconyl-CoA
Oxalate
6.2.1.8
Acetate
1.2.3.5
1.2.1.17
Glyoxylate
4.1.3.1
Isocitrate
Dihydroxy-fumarate
4.1.1.54
4.1.1.47
Tartronate semialdehyde
Succinate
4.1.3.16
1.1.99.14
Glycolate
1.1.3.15
4-Hydroxy-2-oxoglutarate
4.1.3.42
Pyruvate
1.1.1.92
1.1.1.60
5.3.1.22
1.2.1.21
O2
H2O2
4.1.3.13
3-Oxalomalate
Vitamin B6 metabolism
1.11.1.6
Oxaloacetate
Purine metabolism
D-Glycerate
1.1.1.26
1.1.1.79
4.1.1.40
2.3.3.7
3-Ethylmalate
Butanoyl-CoA
1.1.1.29
1.1.1.81
Hydroxy-pyruvate
Glycolaldehyde
3.1.3.18
2.7.1.165
2.7.1.31
1.1.1.77
Phosphoglycolate
2.3.3.12
3-Propylmalate
Pentanoyl-CoA
2-Phospho-D-glycerate
Ethane-1,2-diol
D-Ribulose 1,5-P2
4.1.1.39
Carbon fixation
3-Phospho-D-glycerate
2.2.1.5
2-Hydroxy-3-oxoadipate
2-Oxoglutarate
Alanine, aspartate and glutamate metabolism
L-Glutamine
6.3.1.2
Carbon fixation
2-Oxoglutarate
1.4.7.1
L-Glutamate
Glycolysis / Gluconeogenesis
log2 Normalized Abundance
-4.3
13.4
log2 Normalized Richness
1.1
13.4
(Reads/Gigabase pair)
NH3
GDC
Glycine
2.6.1.4
2.1.2.1
2.6.1.45
L-Serine
E.C. #
60
70
85
GBS Site
E.C. containing at least one
#.#.#.#
differentially abundant KO
Glycine, serine and threonine metabolism
00630 7/5/18
(c) Kanehisa Laboratories

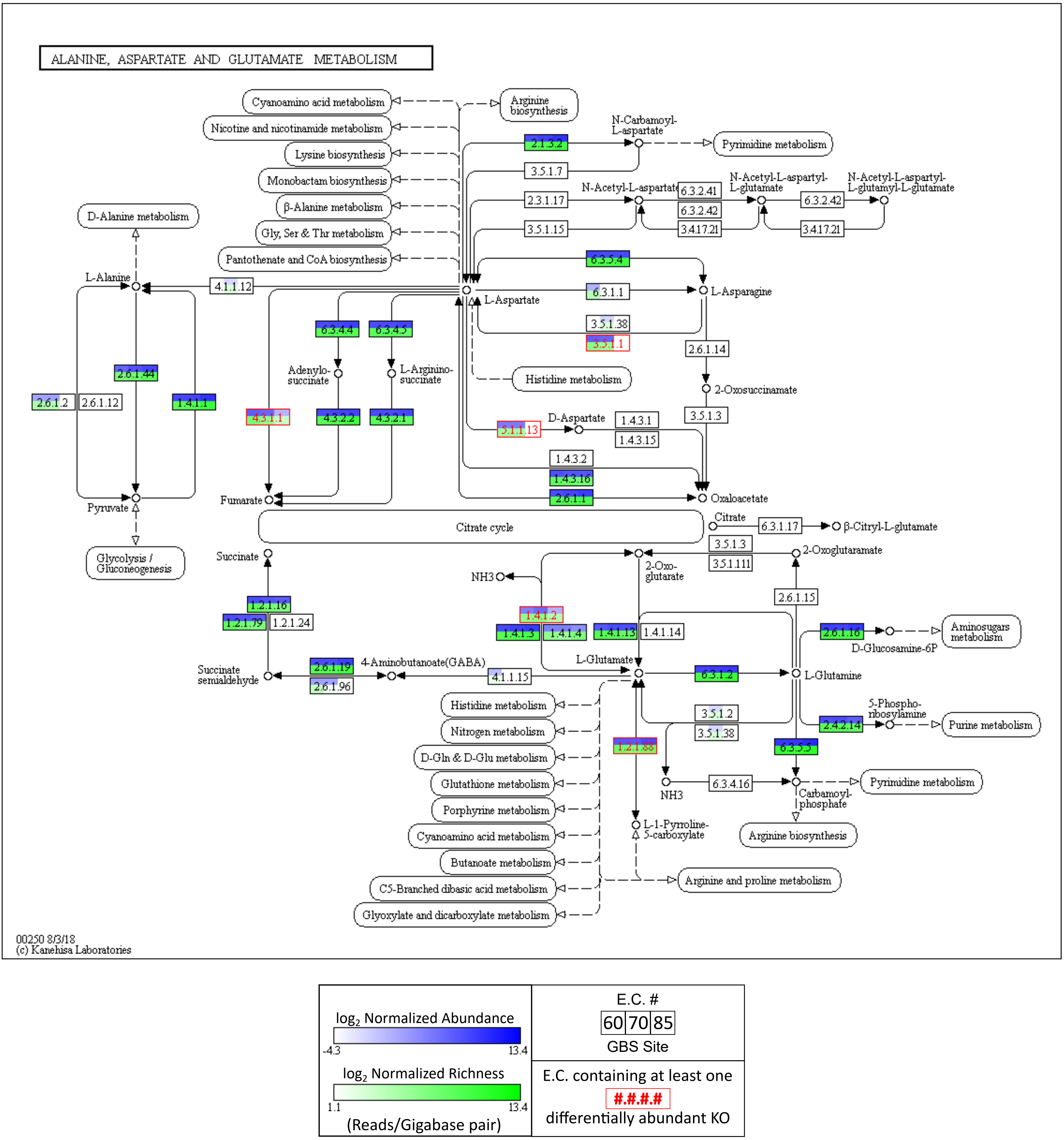
ALANINE, ASPARTATE AND GLUTAMATE METABOLISM
Cyanoamino acid metabolism
Nicotine and nicotinamide metabolism
Lysine biosynthesis
Monobactam biosynthesis
β-Alanine metabolism
Gly, Ser & Thr metabolism
Pantothenate and CoA biosynthesis
Arginine biosynthesis
N-Carbamoyl-L-aspartate
Pyrimidine metabolism
2.1.3.2
3.5.1.7
2.3.1.17
3.5.1.15
N-Acetyl-L-aspartate
6.3.2.41
6.3.2.42
3.4.17.21
N-Acetyl-L-aspartyl-L-glutamate
N-Acetyl-L-aspartyl-L-glutamyl-L-glutamate
D-Alanine metabolism
L-Alanine
4.1.1.12
6.3.5.4
L-Aspartate
6.3.1.1
L-Asparagine
3.5.1.38
3.5.1.1
2.6.1.14
6.3.4.4
6.3.4.5
Adenylo-succinate
L-Arginino-succinate
Histidine metabolism
2-Oxosuccinamate
2.6.1.44
2.6.1.2
2.6.1.12
1.4.1.1
4.3.1.1
4.3.2.2
4.3.2.1
D-Aspartate
5.1.1.13
1.4.3.1
1.4.3.15
3.5.1.3
1.4.3.2
1.4.3.16
2.6.1.1
Pyruvate
Fumarate
Oxaloacetate
Glycolysis / Gluconeogenesis
Citrate cycle
Citrate
6.3.1.17
β-Citryl-L-glutamate
3.5.1.3
3.5.1.111
2-Oxoglutaramate
Succinate
NH3
2-Oxo-glutarate
2.6.1.15
1.4.1.2
1.2.1.16
1.2.1.79
1.2.1.24
1.4.1.3
1.4.1.4
1.4.1.13
1.4.1.14
2.6.1.16
D-Glucosamine-6P
Aminosugars metabolism
Succinate semialdehyde
2.6.1.19
2.6.1.96
4-Aminobutanoate(GABA)
4.1.1.15
L-Glutamate
6.3.1.2
L-Glutamine
5-Phospho-ribosylamine
2.4.2.14
Purine metabolism
Histidine metabolism
Nitrogen metabolism
D-Gln & D-Glu metabolism
Glutathione metabolism
Porphyrine metabolism
Cyanoamino acid metabolism
Butanoate metabolism
C5-Branched dibasic acid metabolism
Glyoxylate and dicarboxylate metabolism
3.5.1.2
3.5.1.38
1.2.1.88
6.3.5.5
NH3
6.3.4.16
Carbamoyl-phosphate
Pyrimidine metabolism
L-1-Pyrroline-5-carboxylate
Arginine biosynthesis
Arginine and proline metabolism
00250 8/3/18
(c) Kanehisa Laboratories
log2 Normalized Abundance
-4.3
13.4
log2 Normalized Richness
1.1
13.4
(Reads/Gigabase pair)
E.C. #
60 70 85
GBS Site
E.C. containing at least one
#.#.#.#
differentially abundant KO

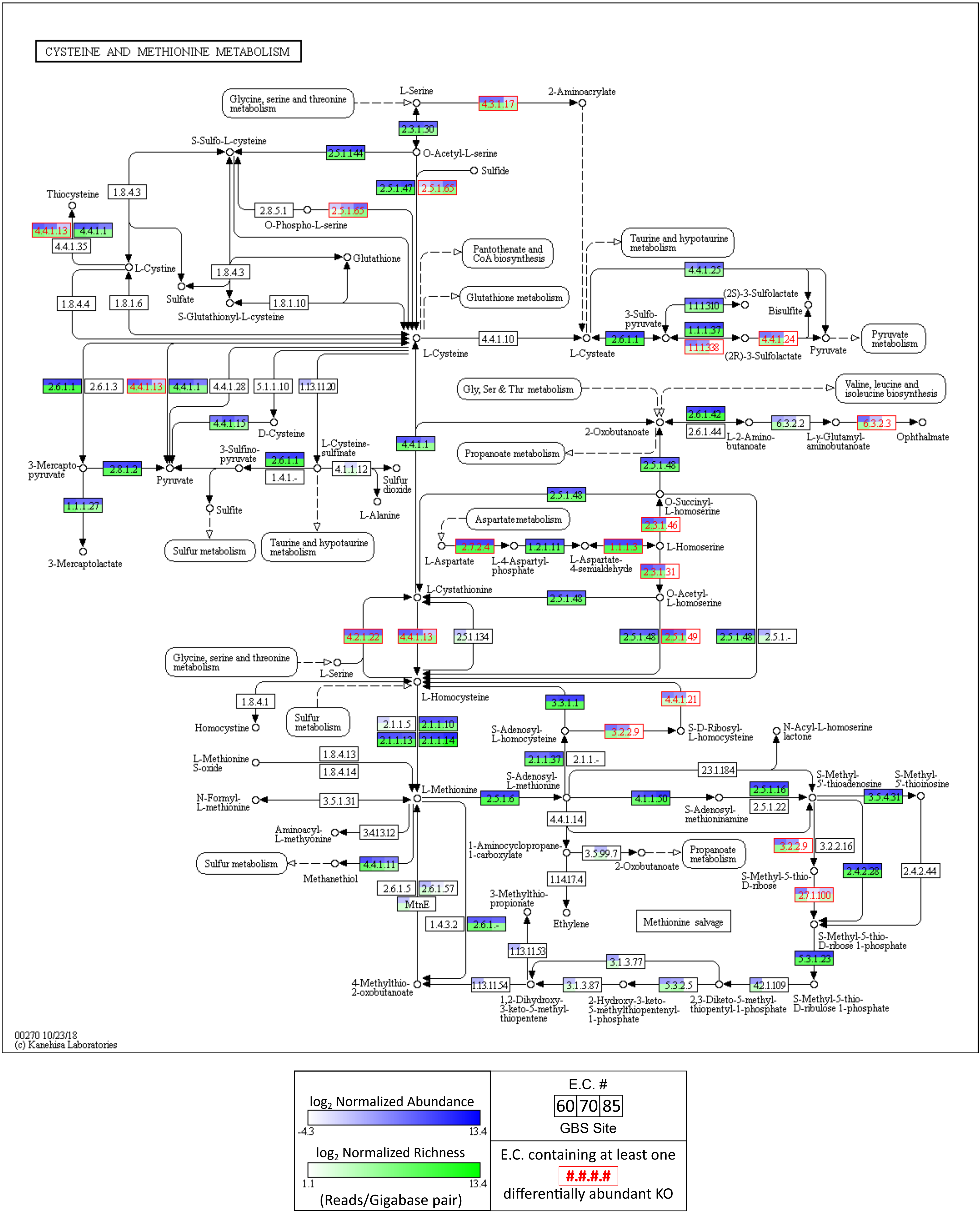

CYSTEINE AND METHIONINE METABOLISM
Glycine, serine and threonine metabolism
L-Serine
4.3.1.17
2-Aminoacrylate
2.3.1.30
S-Sulfo-L-cysteine
2.5.1.144
O-Acetyl-L-serine
Sulfide
2.5.1.47
2.5.1.65
Thiocysteine
1.8.4.3
2.8.5.1
O-Phospho-L-serine
4.4.1.13
4.4.1.1
4.4.1.35
L-Cystine
1.8.4.3
Glutathione
Pantothenate and CoA biosynthesis
Taurine and hypotaurine metabolism
4.4.1.25
Sulfate
S-Glutathionyl-L-cysteine
1.8.4.4
1.8.1.6
1.8.1.10
Glutathione metabolism
(2S)-3-Sulfolactate
1.1.1.310
Bisulfite
3-Sulfo-pyruvate
1.1.1.37
L-Cysteine
4.4.1.10
L-Cysteate
2.6.1.1
1.1.1.338
4.4.1.24
(2R)-3-Sulfolactate
Pyruvate
Pyruvate metabolism
2.6.1.1
2.6.1.3
4.4.1.13
4.4.1.1
4.4.1.28
5.1.1.10
1.13.11.20
Gly, Ser & Thr metabolism
Valine, leucine and isoleucine biosynthesis
2.6.1.42
2.6.1.44
6.3.2.2
6.3.2.3
4.4.1.15
D-Cysteine
2-Oxobutanoate
L-2-Amino-butanoate
L-γ-Glutamyl-aminobutanoate
Ophthalmate
4.4.1.1
L-Cysteine-sulfinate
Propanoate metabolism
3-Sulfino-pyruvate
3-Mercapto-pyruvate
2.8.1.2
Pyruvate
2.6.1.1
4.1.1.12
1.4.1.-
Sulfur dioxide
2.5.1.48
L-Alanine
O-Succinyl-L-homoserine
Sulfite
1.1.1.27
Aspartate metabolism
2.3.1.46
Sulfur metabolism
Taurine and hypotaurine metabolism
2.7.2.4
1.2.1.11
1.1.1.3
L-Homoserine
3-Mercaptolactate
L-Aspartate
L-4-Aspartyl-phosphate
L-Aspartate-4-semialdehyde
2.3.1.31
L-Cystathionine
2.5.1.48
O-Acetyl-L-homoserine
4.2.1.22
4.4.1.13
2.5.1.134
2.5.1.48
2.5.1.49
2.5.1.48
2.5.1.-
Glycine, serine and threonine metabolism
L-Serine
L-Homocysteine
1.8.4.1
Sulfur metabolism
3.3.1.1
4.4.1.21
Homocystine
2.1.1.5
2.1.1.10
2.1.1.13
2.1.1.14
S-Adenosyl-L-homocysteine
3.2.2.9
S-D-Ribosyl-L-homocysteine
N-Acyl-L-homoserine lactone
L-Methionine S-oxide
1.8.4.13
1.8.4.14
2.1.1.37
2.1.1.-
2.3.1.184
S-Adenosyl-L-methionine
S-Methyl-5'-thioadenosine
S-Methyl-5'-thioinosine
N-Formyl-L-methionine
3.5.1.31
L-Methionine
2.5.1.6
4.1.1.50
2.5.1.16
3.5.4.31
S-Adenosyl-methioninamine
2.5.1.22
4.4.1.14
Aminoacyl-L-methionine
3.4.13.12
1-Aminocyclopropane-1-carboxylate
3.5.99.7
2-Oxobutanoate
Propanoate metabolism
3.2.2.9
3.2.2.16
Sulfur metabolism
4.4.1.11
Methanethiol
1.14.17.4
S-Methyl-5-thio-D-ribose
2.4.2.28
2.4.2.44
2.6.1.5
2.6.1.57
MtnE
3-Methylthio-propionate
Ethylene
Methionine salvage
2.7.1.100
1.4.3.2
2.6.1.-
S-Methyl-5-thio-D-ribose 1-phosphate
1.13.11.53
3.1.3.77
5.3.1.23
4-Methylthio-2-oxobutanoate
1.13.11.54
3.1.3.87
5.3.2.5
4.2.1.109
1,2-Dihydroxy-3-keto-5-methyl-thiopentene
2-Hydroxy-3-keto-5-methylthiopentenyl-1-phosphate
2,3-Diketo-5-methyl-thiopentyl-1-phosphate
S-Methyl-5-thio-D-ribulose 1-phosphate
00270 10/23/18
(c) Kanehisa Laboratories
log2 Normalized Abundance
-4.3
13.4
log2 Normalized Richness
1.1
13.4
(Reads/Gigabase pair)
E.C. #
60 70 85
GBS Site
E.C. containing at least one
#.#.#.#
differentially abundant KO

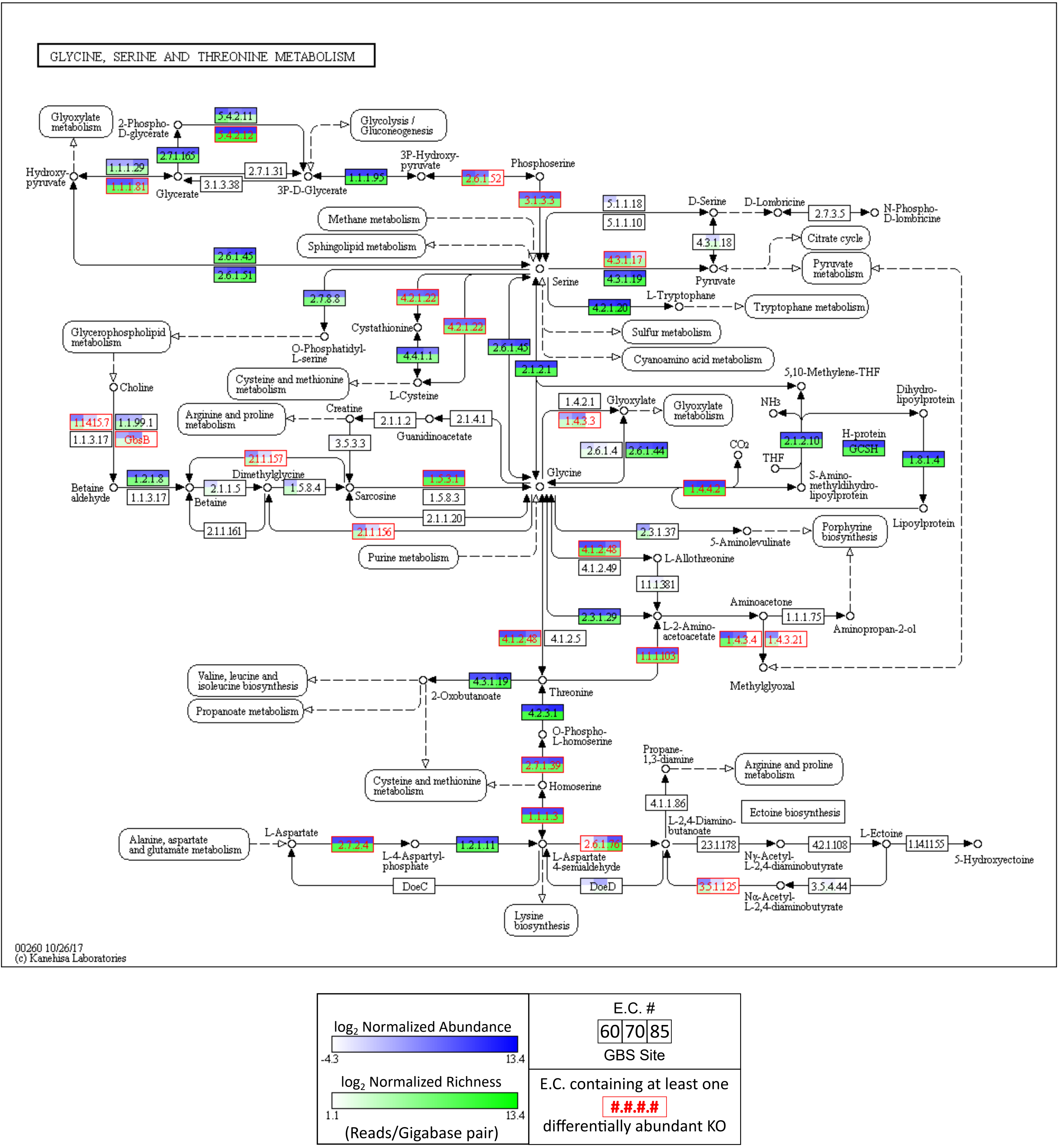
GLYCINE, SERINE AND THREONINE METABOLISM
Glyoxylate metabolism
2-Phospho-D-glycerate
5.4.2.11
5.4.2.12
Glycolysis / Gluconeogenesis
2.7.1.165
3P-Hydroxy-pyruvate
Hydroxy-pyruvate
1.1.1.29
1.1.1.81
2.7.1.31
3.1.3.38
Glycerate
3P-D-Glycerate
1.1.1.95
2.6.1.52
Phosphoserine
3.1.3.3
5.1.1.18
5.1.1.10
D-Serine
D-Lombricine
2.7.3.5
N-Phospho-D-lombricine
Methane metabolism
4.3.1.18
Citrate cycle
Sphingolipid metabolism
2.6.1.45
2.6.1.51
4.3.1.17
4.3.1.19
Serine
Pyruvate
Pyruvate metabolism
2.7.8.8
4.2.1.22
4.2.1.20
L-Tryptophane
Tryptophane metabolism
Glycerophospholipid metabolism
Cystathionine
4.2.1.22
O-Phosphatidyl-L-serine
4.4.1.1
2.6.1.45
Sulfur metabolism
2.1.2.1
Cyanoamino acid metabolism
Choline
Cysteine and methionine metabolism
L-Cysteine
5,10-Methylene-THF
Dihydro-lipoylprotein
1.1.415.7
1.1.99.1
1.1.3.17
GbsB
Arginine and proline metabolism
Creatine
2.1.1.2
2.1.4.1
Guanidinoacetate
1.4.2.1
1.4.3.3
Glyoxylate
Glyoxylate metabolism
NH3
3.5.3.3
2.6.1.4
2.6.1.44
2.1.2.10
H-protein
GCSH
CO2
THF
1.8.1.4
2.1.1.157
Dimethylglycine
1.2.1.8
Betaine aldehyde
1.1.3.17
2.1.1.5
Betaine
1.5.8.4
Sarcosine
1.5.3.1
1.5.8.3
Glycine
1.4.4.2
S-Amino-methyldihydro-lipoylprotein
2.1.1.20
Lipoylprotein
2.1.1.161
2.1.1.156
2.3.1.37
5-Aminolevulinate
Porphyrine biosynthesis
4.1.2.48
4.1.2.49
L-Allothreonine
Purine metabolism
1.1.1.381
Aminoacetone
2.3.1.29
1.1.1.75
Aminopropan-2-ol
L-2-Amino-acetoacetate
4.1.2.48
4.1.2.5
1.4.3.4
1.4.3.21
1.1.1.103
Methylglyoxal
Valine, leucine and isoleucine biosynthesis
4.3.1.19
2-Oxobutanoate
Threonine
Propanoate metabolism
4.2.3.1
O-Phospho-L-homoserine
Propane-1,3-diamine
Arginine and proline metabolism
2.7.1.39
Cysteine and methionine metabolism
Homoserine
4.1.1.86
L-2,4-Diamino-butanoate
Ectoine biosynthesis
1.1.1.3
Alanine, aspartate and glutamate metabolism
L-Aspartate
2.7.2.4
L-4-Aspartyl-phosphate
1.2.1.11
2.6.1.76
L-Aspartate 4-semialdehyde
2.3.1.178
Nγ-Acetyl-L-2,4-diaminobutyrate
4.2.1.108
L-Ectoine
1.14.11.55
5-Hydroxyectoine
DoeC
DoeD
3.5.1.125
Nα-Acetyl-L-2,4-diaminobutyrate
3.5.4.44
Lysine biosynthesis
00260 10/26/17
(c) Kanehisa Laboratories
log2 Normalized Abundance
-4.3
13.4
log2 Normalized Richness
1.1
13.4
(Reads/Gigabase pair)
E.C. #
60 70 85
GBS Site
E.C. containing at least one
#.#.#.#
differentially abundant KO

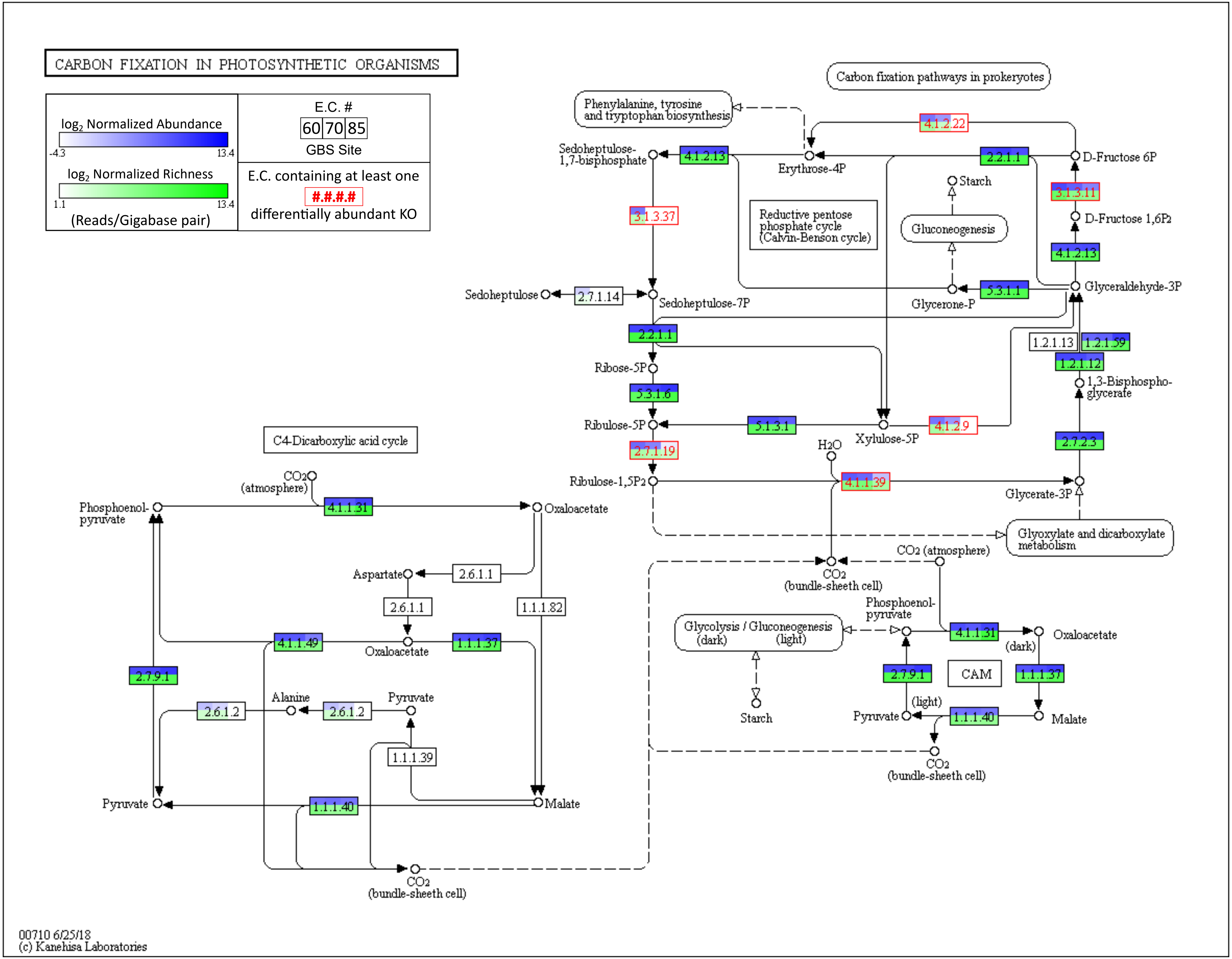
CARBON FIXATION IN PHOTOSYNTHETIC ORGANISMS
log2 Normalized Abundance
-4.3
13.4
log2 Normalized Richness
1.1
13.4
(Reads/Gigabase pair)
E.C. #
60 70 85
GBS Site
E.C. containing at least one
#.#.#.#
differentially abundant KO
Carbon fixation pathways in prokeryotes
Phenylalanine, tyrosine and tryptophan biosynthesis
4.1.2.22
Sedoheptulose-1,7-bisphosphate
4.1.2.13
Erythrose-4P
2.2.1.1
D-Fructose 6P
Starch
3.1.3.11
3.1.3.37
Reductive pentose phosphate cycle (Calvin-Benson cycle)
Gluconeogenesis
D-Fructose 1,6P2
4.1.2.13
Sedoheptulose
2.7.1.14
Sedoheptulose-7P
Glycerone-P
5.3.1.1
Glyceraldehyde-3P
2.2.1.1
1.2.1.13
1.2.1.59
1.2.1.12
Ribose-5P
1,3-Bisphospho-glycerate
5.3.1.6
Ribulose-5P
5.1.3.1
Xylulose-5P
4.1.2.9
2.7.2.3
2.7.1.19
H2O
C4-Dicarboxylic acid cycle
Ribulose-1,5P2
4.1.1.39
Glycerate-3P
CO2 (atmosphere)
Phosphoenol-pyruvate
4.1.1.31
Oxaloacetate
Glyoxylate and dicarboxylate metabolism
CO2 (atmosphere)
Aspartate
2.6.1.1
CO2 (bundle-sheath cell)
2.6.1.1
1.1.1.82
Phosphoenol-pyruvate
Glycolysis / Gluconeogenesis (dark) (light)
4.1.1.31
Oxaloacetate
4.1.1.49
Oxaloacetate
1.1.1.37
(dark)
2.7.9.1
2.7.9.1
CAM
1.1.1.37
Alanine
Pyruvate
(light)
2.6.1.2
2.6.1.2
Starch
Pyruvate
1.1.1.40
Malate
1.1.1.39
CO2 (bundle-sheath cell)
Pyruvate
1.1.1.40
Malate
CO2 (bundle-sheath cell)
00710 6/25/18
(c) Kanehisa Laboratories

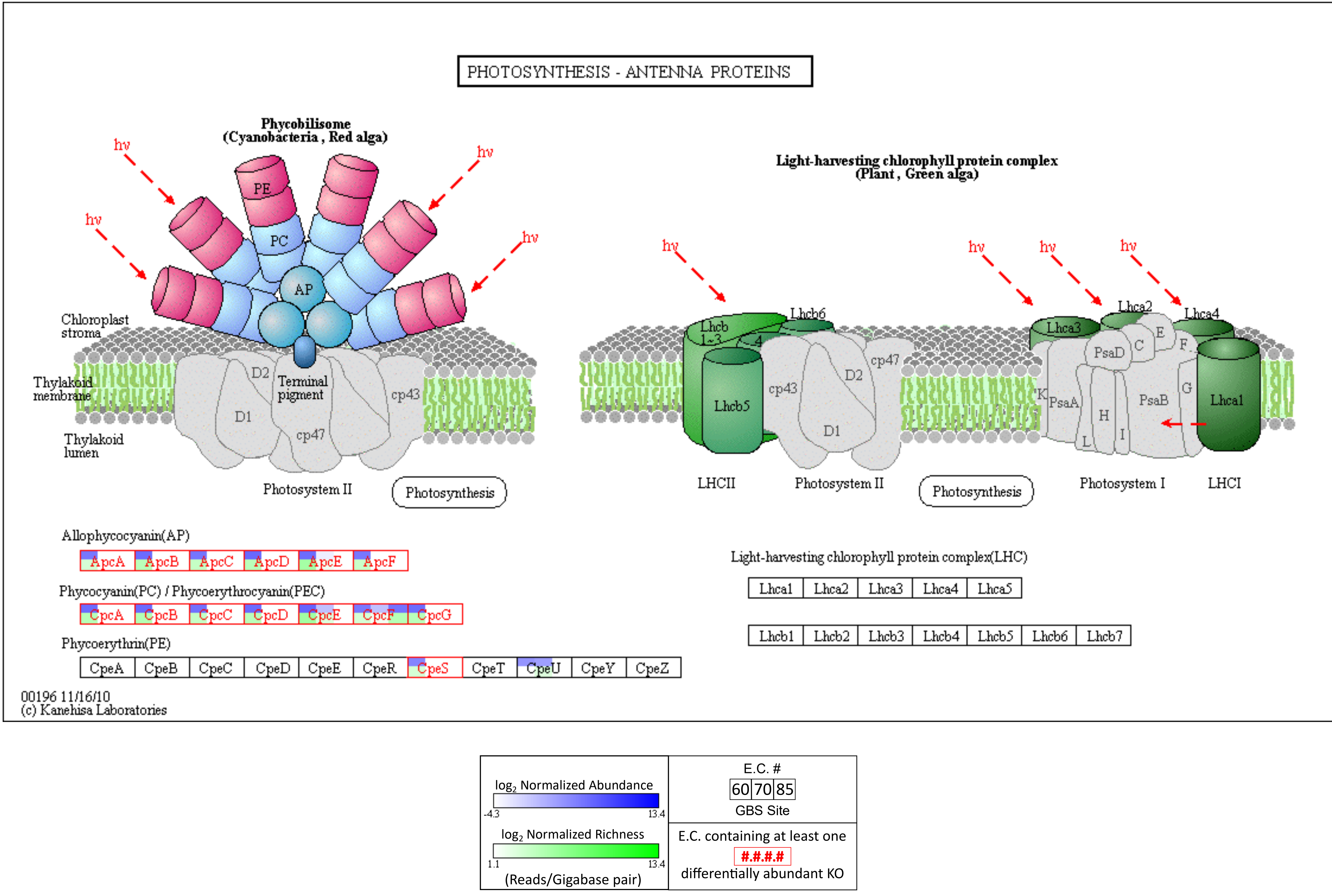
PHOTOSYNTHESIS - ANTENNA PROTEINS
Phycobilisome (Cyanobacteria , Red alga)
hv
PE
PC
AP
Chloroplast stroma
Thylakoid membrane
Thylakoid lumen
D2
D1
Terminal pigment
cp47
cp43
Photosystem II
Photosynthesis
Light-harvesting chlorophyll protein complex (Plant , Green alga)
Lhcb 1~3
4
Lhcb5
Lhcb6
Lhca3
Lhca2
Lhca4
Lhca1
PsaD
PsaA
PsaB
C
E
F
G
H
I
L
K
LHCII
Photosystem I
LHCI
Allophycocyanin(AP)
ApcA ApcB ApcC ApcD ApcE ApcF
Phycocyanin(PC) / Phycoerythrocyanin(PEC)
CpcA CpcB CpcC CpcD CpcE CpcF CpcG
Phycoerythrin(PE)
CpeA CpeB CpeC CpeD CpeE CpeR CpeS CpeT CpeU CpeY CpeZ
Light-harvesting chlorophyll protein complex(LHC)
Lhca1 Lhca2 Lhca3 Lhca4 Lhca5
Lhcb1 Lhcb2 Lhcb3 Lhcb4 Lhcb5 Lhcb6 Lhcb7
00196 11/16/10
(c) Kanehisa Laboratories
log2 Normalized Abundance
-4.3
13.4
log2 Normalized Richness
1.1
13.4
(Reads/Gigabase pair)
E.C. #
60 70 85
GBS Site
E.C. containing at least one
#.#.#.#
differentially abundant KO

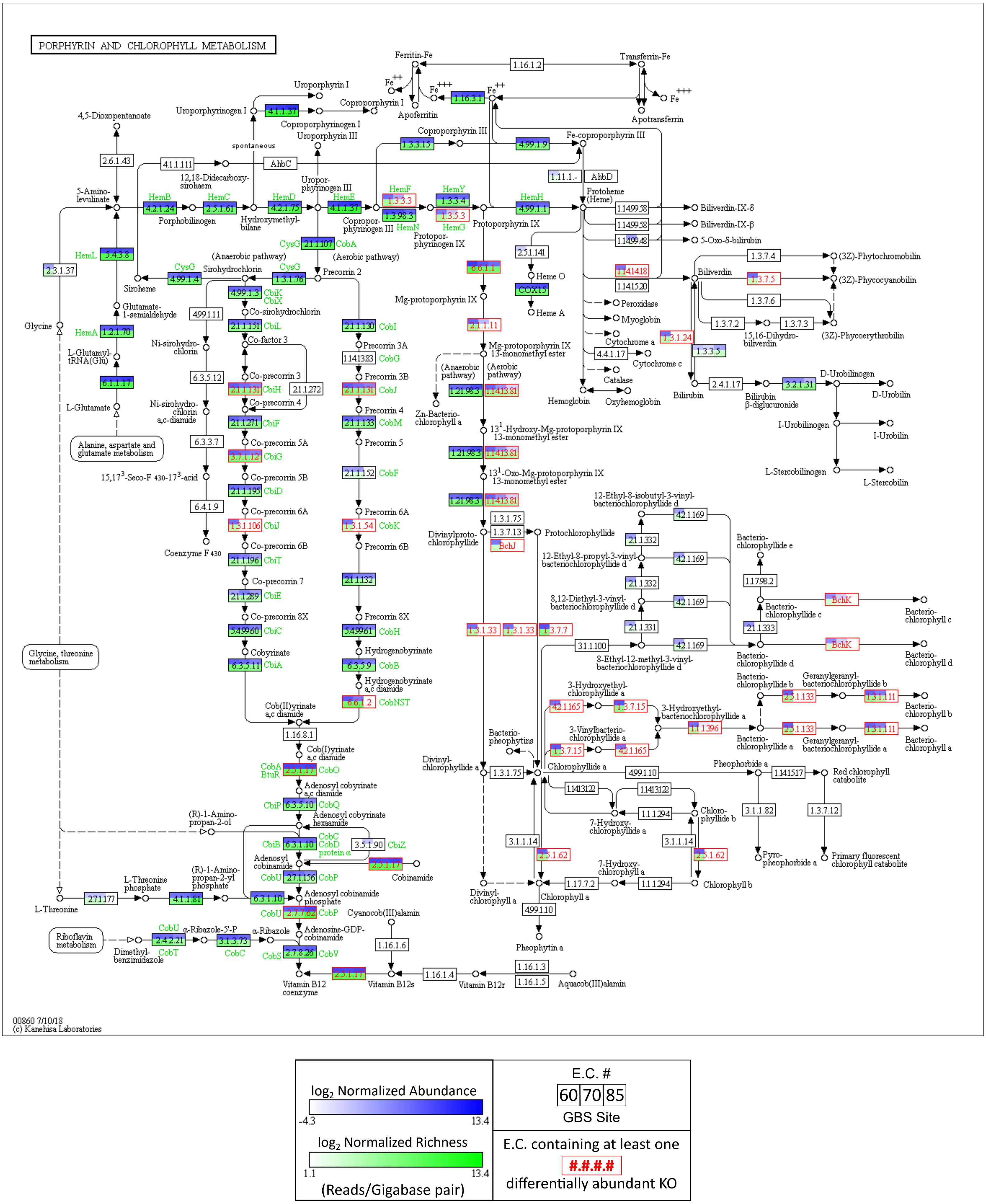

PORPHYRIN AND CHLOROPHYLL METABOLISM
Ferritin-Fe
Transferrin-Fe
Uroporphyrin I
Uroporphyrinogen I
Coproporphyrin I
Coproporphyrinogen I
Uroporphyrin III
Coproporphyrin III
Fe-coproporphyrin III
Apoferritin
Apotransferrin
4,5-Dioxopentanoate
12,18-Didecarboxy-siroheam
5-Amino-levulinate
Porphobilinogen
Hydroxymethyl-bilane
Uropor-phyrinogen III
Copropor-phyrinogen III
Protopor-phyrinogen IX
Protoporphyrin IX
Protoheme (Heme)
Biliverdin-IX-δ
Biliverdin-IX-β
5-Oxo-δ-bilirubin
Siroheme
Sirohydrochlorin
Precorrin 2
Glutamate-1-semialdehyde
L-Glutamyl-tRNA(Glu)
L-Glutamate
Glycine
Alanine, aspartate and glutamate metabolism
Glycine, threonine metabolism
Riboflavin metabolism
Mg-protoporphyrin IX
Mg-protoporphyrin IX 13-monomethyl ester
Heme O
Heme A
Biliverdin
Bilirubin
(3Z)-Phytochromobilin
(3Z)-Phycocyanobilin
(3Z)-Phycoerythrobilin
15,16-Dihydro-biliverdin
Peroxidase
Myoglobin
Cytochrome a
Cytochrome c
Catalase
Hemoglobin
Oxyhemoglobin
D-Urobilinogen
D-Urobilin
Bilirubin β-diglucuronide
I-Urobilinogen
I-Urobilin
L-Stercobilinogen
L-Stercobilin
Divinylproto-chlorophyllide
Protochlorophyllide
Chlorophyllide a
Chlorophyll a
Chlorophyll b
Pheophorbide a
Red chlorophyll catabolite
Pyro-pheophorbide a
Primary fluorescent chlorophyll catabolite
Pheophytin a
Bacterio-chlorophyll a
Bacterio-chlorophyll b
Bacterio-chlorophyll c
Bacterio-chlorophyll d
Vitamin B12 coenzyme
Vitamin B12s
Vitamin B12r
Aquacob(III)alamin
Cyanocob(III)alamin
L-Threonine
00860 7/10/18
(c) Kanehisa Laboratories
log2 Normalized Abundance
-4.3
13.4
log2 Normalized Richness
1.1
13.4
(Reads/Gigabase pair)
E.C. #
60 70 85
GBS Site
E.C. containing at least one
#.#.#.#
differentially abundant KO

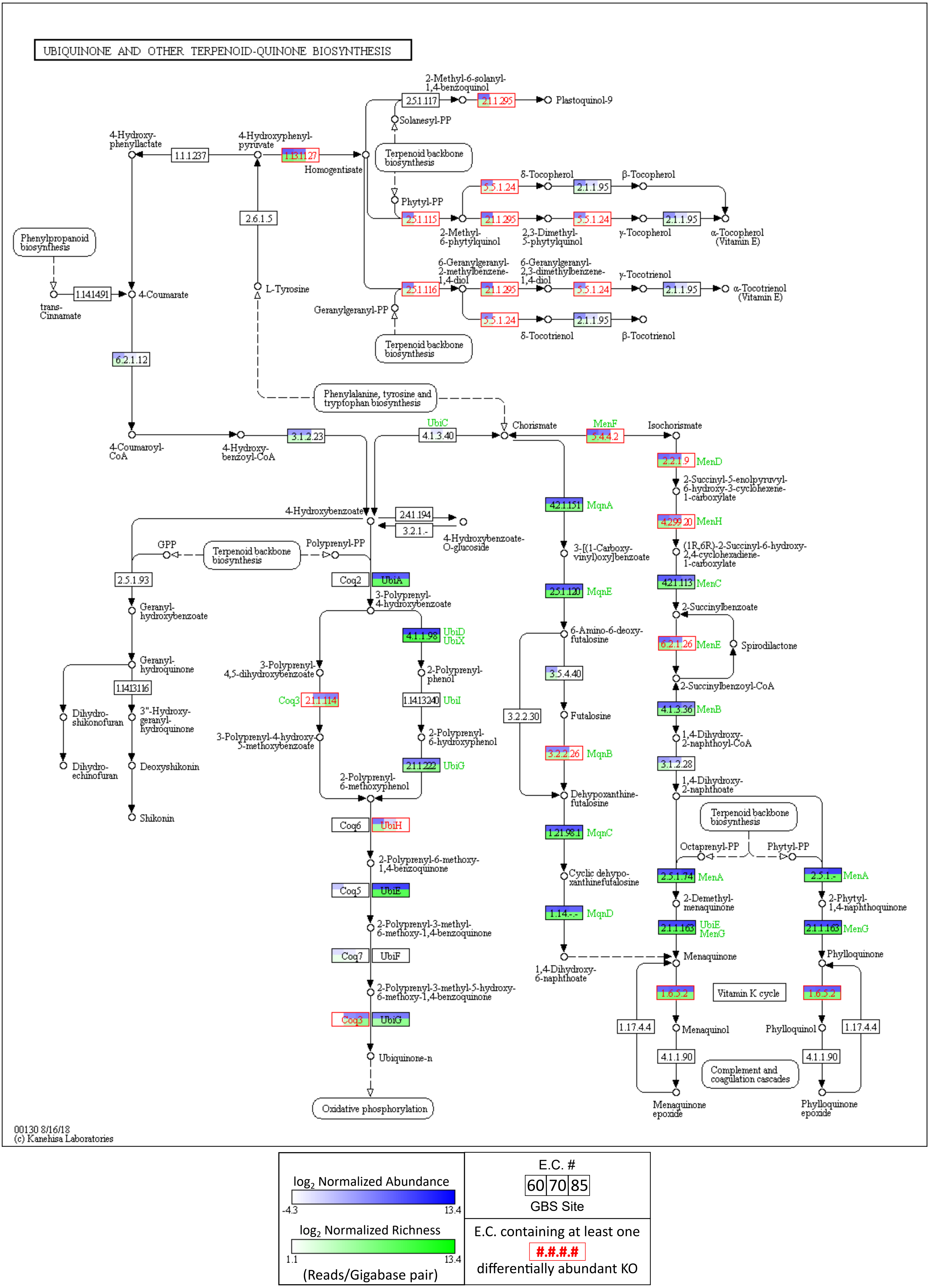
UBIQUINONE AND OTHER TERPENOID-QUINONE BIOSYNTHESIS
2-Methyl-6-solanyl-1,4-benzoquinol
2.5.1.117
2.1.1.295
Plastoquinol-9
Solanesyl-PP
4-Hydroxy-phenyllactate
1.1.1.237
4-Hydroxyphenyl-pyruvate
1.13.11.27
Homogentisate
Terpenoid backbone biosynthesis
δ-Tocopherol
β-Tocopherol
5.5.1.24
2.1.1.95
Phytyl-PP
2.5.1.115
2-Methyl-6-phytylquinol
2,3-Dimethyl-5-phytylquinol
γ-Tocopherol
α-Tocopherol (Vitamin E)
2.6.1.5
Phenylpropanoid biosynthesis
6-Geranylgeranyl-2-methylbenzene-1,4-diol
6-Geranylgeranyl-2,3-dimethylbenzene-1,4-diol
γ-Tocotrienol
2.5.1.116
α-Tocotrienol (Vitamin E)
trans-Cinnamate
1.14.14.91
4-Coumarate
L-Tyrosine
Geranylgeranyl-PP
δ-Tocotrienol
β-Tocotrienol
6.2.1.12
Phenylalanine, tyrosine and tryptophan biosynthesis
UbiC
4.1.3.40
Chorismate
MenF
5.4.4.2
Isochorismate
4-Coumaroyl-CoA
4-Hydroxy-benzoyl-CoA
3.1.2.23
2.2.1.9
MenD
2-Succinyl-5-enolpyruvyl-6-hydroxy-3-cyclohexene-1-carboxylate
4.2.1.151
MqnA
4-Hydroxybenzoate
2.4.1.194
3.2.1.-
4-Hydroxybenzoate-O-glucoside
4.2.99.20
MenH
3-[(1-Carboxy-vinyl)oxy]benzoate
(1R,6R)-2-Succinyl-6-hydroxy-2,4-cyclohexadiene-1-carboxylate
GPP
Polyprenyl-PP
2.5.1.93
Coq2
UbiA
2.5.1.120
MqnE
4.2.1.113
MenC
Geranyl-hydroxybenzoate
3-Polyprenyl-4-hydroxybenzoate
2-Succinylbenzoate
6-Amino-6-deoxy-futalosine
4.1.1.98
UbiD
UbiX
6.2.1.26
MenE
Spirodilactone
Geranyl-hydroquinone
3-Polyprenyl-4,5-dihydroxybenzoate
2-Polyprenyl-phenol
3.5.4.40
2-Succinylbenzoyl-CoA
1.14.13.116
Coq3
2.1.1.114
1.14.13.240
UbiI
3.2.2.30
Futalosine
4.1.3.36
MenB
Dihydro-shikonofuran
3''-Hydroxy-geranyl-hydroquinone
3-Polyprenyl-4-hydroxy-5-methoxybenzoate
2-Polyprenyl-6-hydroxyphenol
1,4-Dihydroxy-2-naphthoyl-CoA
3.2.2.26
MqnB
3.1.2.28
Dihydro-echinofuran
Deoxyshikonin
2.1.1.222
UbiG
2-Polyprenyl-6-methoxyphenol
1,4-Dihydroxy-2-naphthoate
Dehypoxanthine-futalosine
Shikonin
Coq6
UbiH
1.21.98.1
MqnC
2-Polyprenyl-6-methoxy-1,4-benzoquinone
Octaprenyl-PP
Phytyl-PP
Cyclic dehypo-xanthinefutalosine
Coq5
UbiE
2.5.1.74
MenA
2.5.1.-
2-Demethyl-menaquinone
2-Phytyl-1,4-naphthoquinone
2-Polyprenyl-3-methyl-6-methoxy-1,4-benzoquinone
1.14.-.-
MqnD
2.1.1.163
UbiE
MenG
Coq7
UbiF
Menaquinone
Phylloquinone
2-Polyprenyl-3-methyl-5-hydroxy-6-methoxy-1,4-benzoquinone
1,4-Dihydroxy-6-naphthoate
1.6.5.2
Vitamin K cycle
UbiG
1.17.4.4
Menaquinol
Phylloquinol
Ubiquinone-n
4.1.1.90
Complement and coagulation cascades
Oxidative phosphorylation
Menaquinone epoxide
Phylloquinone epoxide
00130 8/16/18
(c) Kanehisa Laboratories
log2 Normalized Abundance
-4.3
13.4
log2 Normalized Richness
1.1
13.4
(Reads/Gigabase pair)
E.C. #
60
70
85
GBS Site
E.C. containing at least one
#.#.#.#
differentially abundant KO

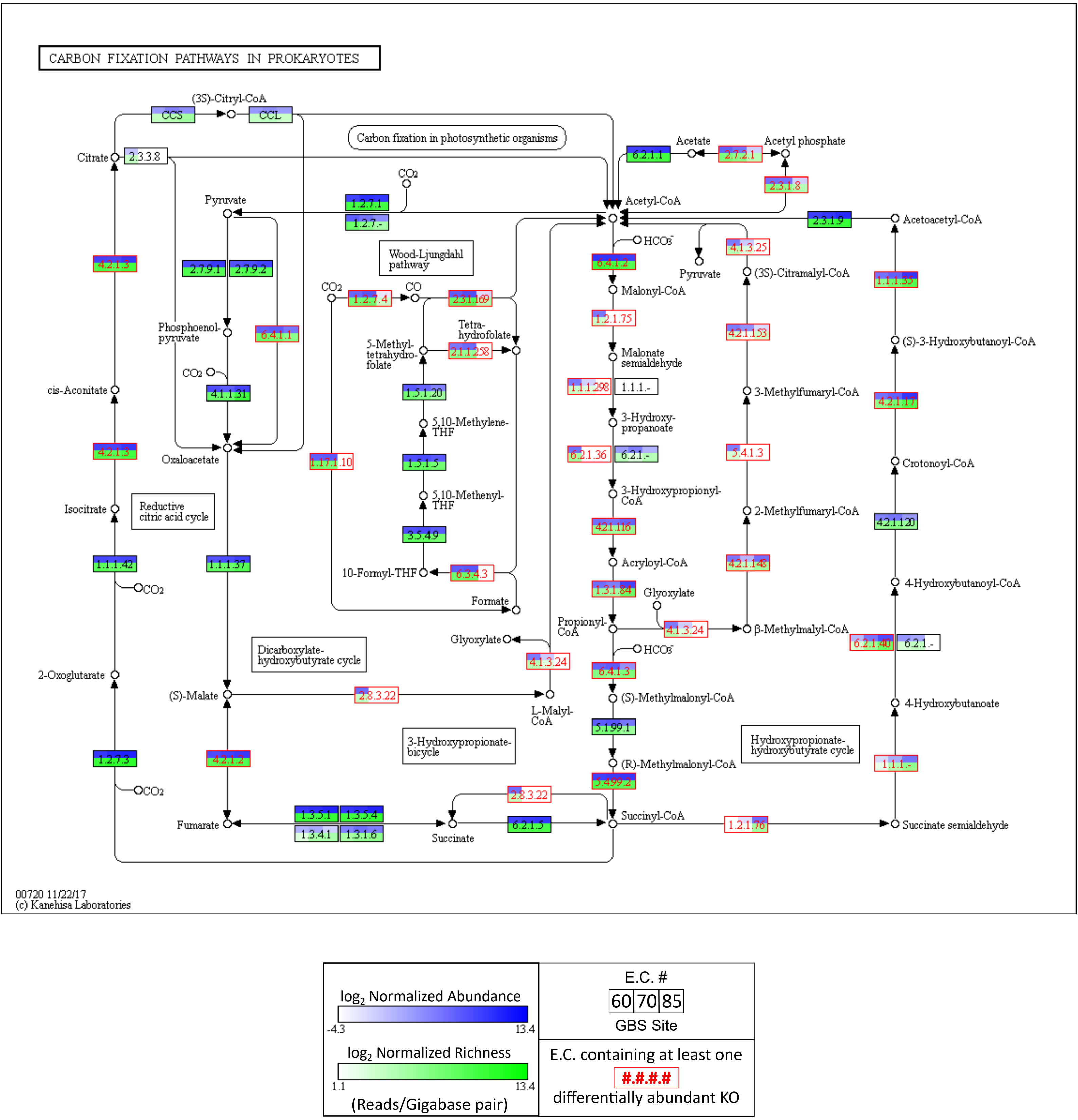
CARBON FIXATION PATHWAYS IN PROKARYOTES
(3S)-Citryl-CoA
CCS
CCL
Citrate
2.3.3.8
Carbon fixation in photosynthetic organisms
Acetate
Acetyl phosphate
6.2.1.1
2.7.2.1
2.3.1.8
CO2
Pyruvate
1.2.7.1
1.2.7.-
Acetyl-CoA
2.3.1.9
Acetoacetyl-CoA
HCO3-
6.4.1.2
Pyruvate
4.1.3.25
(3S)-Citramalyl-CoA
1.1.1.35
Wood-Ljungdahl pathway
4.2.1.3
2.7.9.1
2.7.9.2
Phosphoenol-pyruvate
6.4.1.1
CO2
1.2.7.4
CO
2.3.1.169
Tetra-hydrofolate
5-Methyl-tetrahydro-folate
2.1.1.258
Malonyl-CoA
1.2.1.75
Malonate semialdehyde
4.2.1.153
(S)-3-Hydroxybutanoyl-CoA
CO2
4.1.1.31
cis-Aconitate
1.5.1.20
1.1.1.298
1.1.1.-
3-Methylfumaryl-CoA
4.2.1.17
5,10-Methylene-THF
3-Hydroxy-propanoate
Crotonoyl-CoA
4.2.1.3
Oxaloacetate
1.17.1.10
1.5.1.5
6.2.1.36
6.2.1.-
5.4.1.3
Isocitrate
Reductive citric acid cycle
5,10-Methenyl-THF
3-Hydroxypropionyl-CoA
2-Methylfumaryl-CoA
4.2.1.120
3.5.4.9
4.2.1.116
1.1.1.42
1.1.1.37
10-Formyl-THF
6.3.4.3
Acryloyl-CoA
4.2.1.148
CO2
4-Hydroxybutanoyl-CoA
1.3.1.84
Glyoxylate
Formate
Propionyl-CoA
4.1.3.24
β-Methylmalyl-CoA
Glyoxylate
6.2.1.40
6.2.1.-
Dicarboxylate-hydroxybutyrate cycle
4.1.3.24
HCO3-
6.4.1.3
2-Oxoglutarate
(S)-Malate
2.8.3.22
L-Malyl-CoA
(S)-Methylmalonyl-CoA
4-Hydroxybutanoate
3-Hydroxypropionate-bicycle
5.1.99.1
Hydroxypropionate-hydroxybutyrate cycle
1.2.7.3
4.2.1.2
(R)-Methylmalonyl-CoA
1.1.1.-
CO2
5.4.99.2
2.8.3.22
1.3.5.1
1.3.5.4
1.3.4.1
1.3.1.6
Fumarate
6.2.1.5
Succinyl-CoA
1.2.1.76
Succinate semialdehyde
Succinate
00720 11/22/17
(c) Kanehisa Laboratories
log2 Normalized Abundance
-4.3
13.4
log2 Normalized Richness
1.1
13.4
(Reads/Gigabase pair)
E.C. #
60
70
85
GBS Site
E.C. containing at least one
#.#.#.#
differentially abundant KO

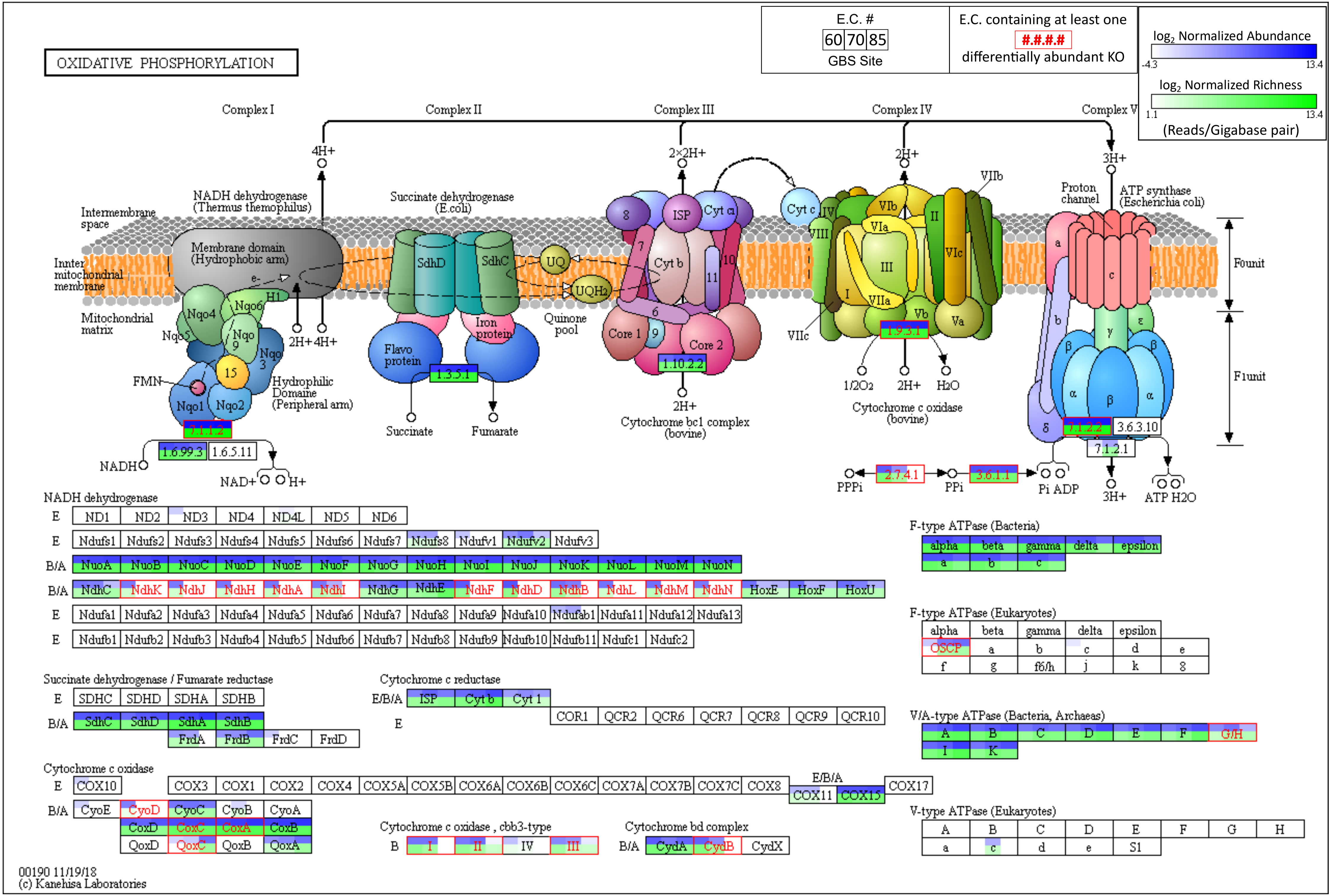
OXIDATIVE PHOSPHORYLATION
E.C. #
60 70 85
GBS Site
E.C. containing at least one
#.#.#.#
differentially abundant KO
log2 Normalized Abundance
-4.3
13.4
log2 Normalized Richness
1.1
13.4
(Reads/Gigabase pair)
Complex I
Complex II
Complex III
Complex IV
Complex V
4H+
2×2H+
2H+
3H+
NADH dehydrogenase (Thermus themophilus)
Succinate dehydrogenase (E.coli)
Intermembrane space
Innter mitochondrial membrane
Mitochondrial matrix
Membrane domain (Hydrophobic arm)
Hydrophilic Domaine (Peripheral arm)
FMN
7.1.1.2
1.6.99.3
1.6.5.11
NADH
NAD+
H+
2H+ 4H+
Flavo protein
Iron protein
SdhD
SdhC
UQ
UQH2
Quinone pool
1.3.5.1
Succinate
Fumarate
Core 1
Core 2
Cyt b
ISP
Cyt c1
Cyt c
1.10.2.2
2H+
Cytochrome bc1 complex (bovine)
1.9.3.1
1/2O2
2H+
H2O
Cytochrome c oxidase (bovine)
Proton channel
ATP synthase (Escherichia coli)
Fo unit
F1 unit
7.1.2.2
3.6.3.10
7.1.2.1
2.7.4.1
3.6.1.1
PPPi
PPi
Pi ADP
3H+
ATP H2O
NADH dehydrogenase
E ND1 ND2 ND3 ND4 ND4L ND5 ND6
E Ndufs1 Ndufs2 Ndufs3 Ndufs4 Ndufs5 Ndufs6 Ndufs7 Ndufs8 Ndufv1 Ndufv2 Ndufv3
B/A NuoA NuoB NuoC NuoD NuoE NuoF NuoG NuoH NuoI NuoJ NuoK NuoL NuoM NuoN
B/A NdhC NdhK NdhJ NdhH NdhA NdhI NdhG NdhE NdhF NdhD NdhB NdhL NdhM NdhN HoxE HoxF HoxU
E Ndufa1 Ndufa2 Ndufa3 Ndufa4 Ndufa5 Ndufa6 Ndufa7 Ndufa8 Ndufa9 Ndufa10 Ndufab1 Ndufa11 Ndufa12 Ndufa13
E Ndufb1 Ndufb2 Ndufb3 Ndufb4 Ndufb5 Ndufb6 Ndufb7 Ndufb8 Ndufb9 Ndufb10 Ndufb11 Ndufc1 Ndufc2
Succinate dehydrogenase / Fumarate reductase
E SDHC SDHD SDHA SDHB
B/A SdhC SdhD SdhA SdhB
FrdA FrdB FrdC FrdD
Cytochrome c reductase
E/B/A ISP Cyt b Cyt 1
E COR1 QCR2 QCR6 QCR7 QCR8 QCR9 QCR10
Cytochrome c oxidase
E COX10 COX3 COX1 COX2 COX4 COX5A COX5B COX6A COX6B COX6C COX7A COX7B COX7C COX8
E/B/A COX11 COX15 COX17
B/A CyoE CyoD CyoC CyoB CyoA
CoxD CoxC CoxA CoxB
QoxD QoxC QoxB QoxA
Cytochrome c oxidase , cbb3-type
B I II IV III
Cytochrome bd complex
B/A CydA CydB CydX
F-type ATPase (Bacteria)
alpha beta gamma delta epsilon
a b c
F-type ATPase (Eukaryotes)
alpha beta gamma delta epsilon
OSCP a b c d e
f g f6/h j k 8
V/A-type ATPase (Bacteria, Archaeas)
A B C D E F G/H
I K
V-type ATPase (Eukaryotes)
A B C D E F G H
a c d e S1
00190 11/19/18
(c) Kanehisa Laboratories

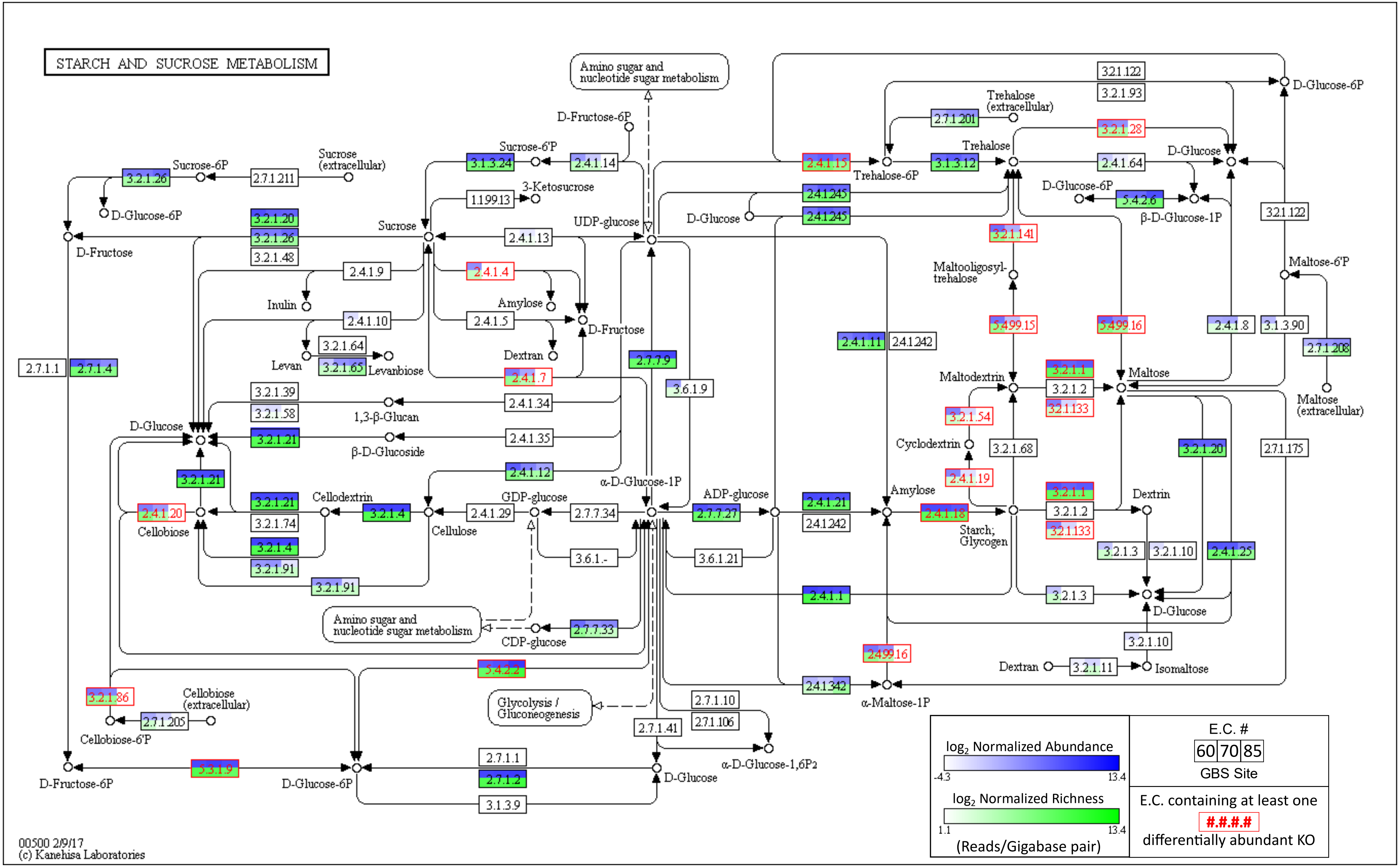
STARCH AND SUCROSE METABOLISM
Amino sugar and nucleotide sugar metabolism
D-Fructose-6P
Sucrose-6'P
Sucrose-6P
Sucrose (extracellular)
D-Glucose-6P
D-Fructose
Sucrose
3-Ketosucrose
UDP-glucose
Inulin
Levan
Levanbiose
Amylose
D-Fructose
Dextran
1,3-β-Glucan
β-D-Glucoside
D-Glucose
Cellodextrin
Cellobiose
Cellulose
GDP-glucose
α-D-Glucose-1P
ADP-glucose
CDP-glucose
Glycolysis / Gluconeogenesis
Cellobiose (extracellular)
Cellobiose-6'P
D-Fructose-6P
D-Glucose-6P
D-Glucose
α-D-Glucose-1,6P2
Trehalose (extracellular)
Trehalose
Trehalose-6P
D-Glucose-6P
β-D-Glucose-1P
Maltose-6'P
Maltooligosyl-trehalose
Maltodextrin
Maltose
Maltose (extracellular)
Cyclodextrin
Amylose
Starch; Glycogen
Dextrin
D-Glucose
Dextran
Isomaltose
α-Maltose-1P
2.7.1.1
2.7.1.4
3.2.1.26
2.7.1.211
3.2.1.20
3.2.1.26
3.2.1.48
3.1.3.24
2.4.1.14
1.1.99.13
2.4.1.13
2.4.1.9
2.4.1.4
2.4.1.10
2.4.1.5
3.2.1.64
3.2.1.65
2.4.1.7
2.7.7.9
3.6.1.9
3.2.1.39
3.2.1.58
2.4.1.34
2.4.1.35
3.2.1.21
3.2.1.21
2.4.1.12
3.2.1.21
3.2.1.74
3.2.1.4
3.2.1.91
3.2.1.4
2.4.1.20
3.2.1.91
2.4.1.29
2.7.7.34
3.6.1.-
2.7.7.27
3.6.1.21
2.7.7.33
3.4.2.2
3.2.1.86
2.7.1.205
5.3.1.9
2.7.1.1
2.7.1.2
3.1.3.9
2.7.1.41
2.7.1.10
2.7.1.106
2.4.1.15
3.1.3.12
2.7.1.201
3.2.1.122
3.2.1.93
3.2.1.28
2.4.1.64
2.4.1.245
2.4.1.245
5.4.2.6
3.2.1.122
3.2.1.141
5.4.99.15
5.4.99.16
2.4.1.8
3.1.3.90
2.7.1.208
2.4.1.11
2.4.1.242
3.2.1.1
3.2.1.2
3.2.1.133
3.2.1.54
3.2.1.68
3.2.1.20
2.7.1.175
2.4.1.19
2.4.1.21
2.4.1.242
2.4.1.18
3.2.1.1
3.2.1.2
3.2.1.133
3.2.1.3
3.2.1.10
2.4.1.25
2.4.1.1
3.2.1.3
3.2.1.10
2.4.99.16
3.2.1.11
2.4.1.342
log2 Normalized Abundance
-4.3
13.4
log2 Normalized Richness
1.1
13.4
(Reads/Gigabase pair)
E.C. #
60 70 85
GBS Site
E.C. containing at least one
#.#.#.#
differentially abundant KO
00500 2/9/17
(c) Kanehisa Laboratories

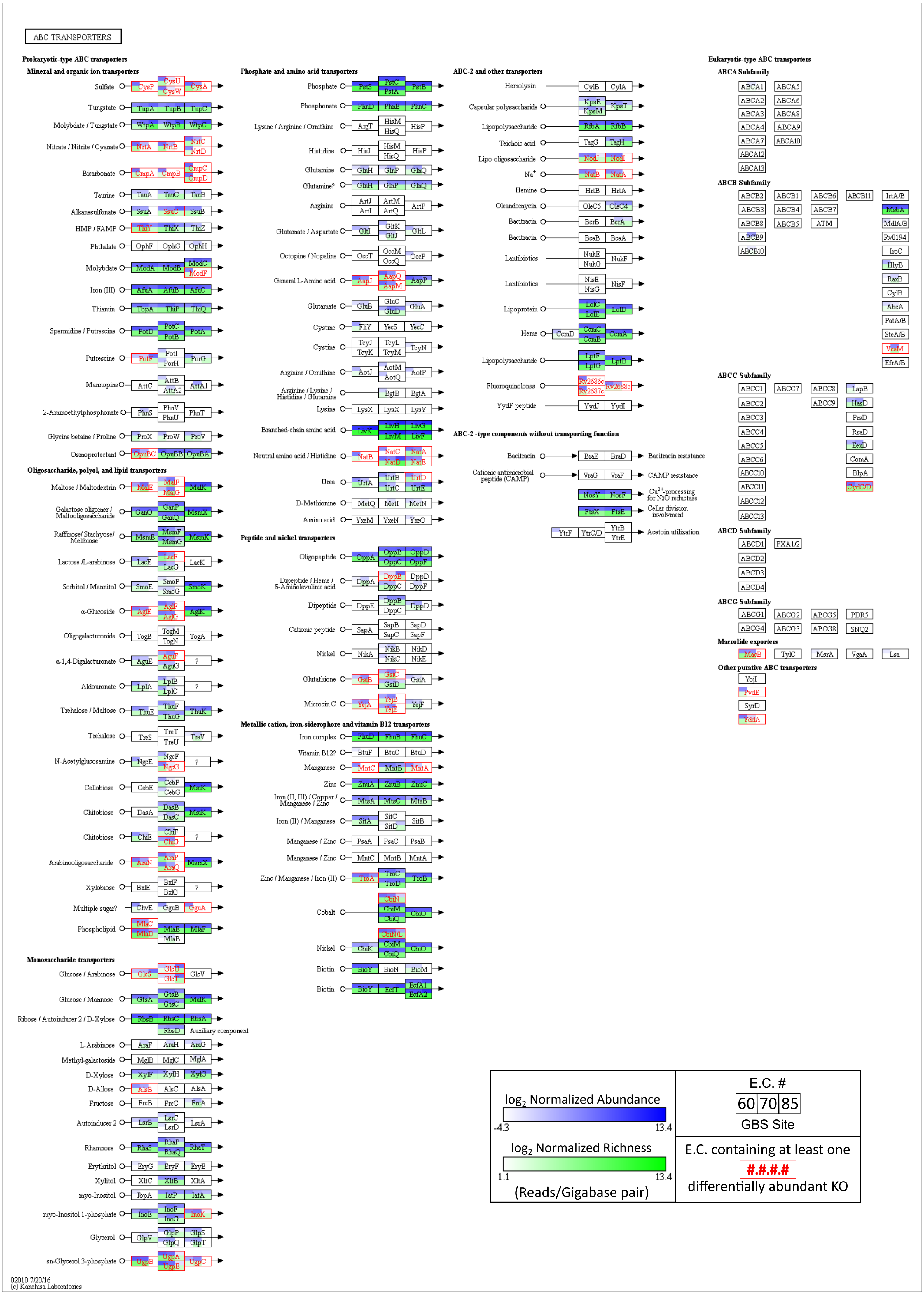

ABC TRANSPORTERS
Prokaryotic-type ABC transporters
Mineral and organic ion transporters
Sulfate CysP CysU CysW CysA
Tungstate TupA TupB TupC
Molybdate / Tungstate WtpA WtpB WtpC
Nitrate / Nitrite / Cyanate NrtA NrtB NrtC NrtD
Bicarbonate CmpA CmpB CmpC CmpD
Taurine TauA TauC TauB
Alkanesulfonate SsuA SsuC SsuB
HMP / FAMP ThiY ThiX ThiZ
Phthalate OphF OphG OphH
Molybdate ModA ModB ModC ModF
Iron (III) AfuA AfuB AfuC
Thiamin TbpA ThiP ThiQ
Spermidine / Putrescine PotD PotC PotB PotA
Putrescine PotF PotI PotH PotG
Mannopine AttC AttB AttA2 AttA1
2-Aminoethylphosphonate PhnS PhnV PhnU PhnT
Glycine betaine / Proline ProX ProW ProV
Osmoprotectant OpuBC OpuBB OpuBA
Oligosaccharide, polyol, and lipid transporters
Maltose / Maltodextrin MalE MalF MalG MalK
Galactose oligomer / Maltooligosaccharide GanO GanP GanQ MsmX
Raffinose/ Stachyose/ Melibiose MsmE MsmF MsmG MsmK
Lactose /L-arabinose LacE LacF LacG LacK
Sorbitol / Mannitol SmoE SmoF SmoG SmoK
α-Glucoside AglE AglF AglG AglK
Oligogalacturonide TogB TogM TogN TogA
α-1,4-Digalacturonate AguE AguF AguG ?
Aldouronate LplA LplB LplC ?
Trehalose / Maltose ThuE ThuF ThuG ThuK
Trehalose TreS TreT TreU TreV
N-Acetylglucosamine NgcE NgcF NgcG ?
Cellobiose CebE CebF CebG MsiK
Chitobiose DasA DasB DasC MsiK
Chitobiose ChiE ChiF ChiG ?
Arabinooligosaccharide AraN AraP AraQ MsmX
Xylobiose BxlE BxlF BxlG ?
Multiple sugar? ChvE GguB GguA
Phospholipid MlaC MlaD MlaE MlaB MlaF
Monosaccharide transporters
Glucose / Arabinose GlcS GlcU GlcT GlcV
Glucose / Mannose GtsA GtsB GtsC MalK
Ribose / Autoinducer 2 / D-Xylose RbsB RbsC RbsA RbsD Auxiliary component
L-Arabinose AraF AraH AraG
Methyl-galactoside MglB MglC MglA
D-Xylose XylF XylH XylG
D-Allose AlsB AlsC AlsA
Fructose FrcB FrcC FrcA
Autoinducer 2 LsrB LsrC LsrD LsrA
Rhamnose RhaS RhaP RhaQ RhaT
Erythritol EryG EryF EryE
Xylitol XltC XltB XltA
myo-Inositol IbpA IatP IatA
myo-Inositol 1-phosphate InoE InoF InoG InoK
Glycerol GlpV GlpP GlpQ GlpS GlpT
sn-Glycerol 3-phosphate UgpB UgpA UgpE UgpC
Phosphate and amino acid transporters
Phosphate PstS PstC PstA PstB
Phosphonate PhnD PhnE PhnC
Lysine / Arginine / Ornithine ArgT HisM HisQ HisP
Histidine HisJ HisM HisQ HisP
Glutamine GlnH GlnP GlnQ
Glutamine? GlnH GlnP GlnQ
Arginine ArtJ ArtI ArtM ArtQ ArtP
Glutamate / Aspartate GltI GltK GltJ GltL
Octopine / Nopaline OccT OccM OccQ OccP
General L-Amino acid AapJ AapQ AapM AapP
Glutamate GluB GluC GluD GluA
Cystine FliY YecS YecC
Cystine TcyJ TcyK TcyL TcyM TcyN
Arginine / Ornithine AotJ AotM AotQ AotP
Arginine / Lysine / Histidine / Glutamine BgtB BgtA
Lysine LysX LysX LysY
Branched-chain amino acid LivK LivH LivM LivG LivF
Neutral amino acid / Histidine NatB NatC NatD NatA NatE
Urea UrtA UrtB UrtC UrtD UrtE
D-Methionine MetQ MetI MetN
Amino acid YxeM YxeN YxeO
Peptide and nickel transporters
Oligopeptide OppA OppB OppC OppD OppF
Dipeptide / Heme / δ-Aminolevulinic acid DppA DppB DppC DppD DppF
Dipeptide DppE DppB DppC DppD
Cationic peptide SapA SapB SapC SapD SapF
Nickel NikA NikB NikC NikD NikE
Glutathione GsiB GsiC GsiD GsiA
Microcin C YejA YejB YejE YejF
Metallic cation, iron-siderophore and vitamin B12 transporters
Iron complex FhuD FhuB FhuC
Vitamin B12? BtuF BtuC BtuD
Manganese MntC MntB MntA
Zinc ZnuA ZnuB ZnuC
Iron (II, III) / Copper / Manganese / Zinc MtsA MtsC MtsB
Iron (II) / Manganese SitA SitC SitD SitB
Manganese / Zinc PsaA PsaC PsaB
Manganese / Zinc MntC MntB MntA
Zinc / Manganese / Iron (II) TroA TroC TroD TroB
Cobalt CbiN CbiM CbiQ CbiO
Nickel CbiK CbiN/L CbiM CbiQ CbiO
Biotin BioY BioN BioM
Biotin BioY EcfT EcfA1 EcfA2
ABC-2 and other transporters
Hemolysin CylB CylA
Capsular polysaccharide KpsE KpsM KpsT
Lipopolysaccharide RfbA RfbB
Teichoic acid TagG TagH
Lipo-oligosaccharide NodJ NodI
Na+ NatB NatA
Hemine HrtB HrtA
Oleandomycin OleC5 OleC4
Bacitracin BcrB BcrA
Bacitracin BceB BceA
Lantibiotics NukE NukG NukF
Lantibiotics NisE NisG NisF
Lipoprotein LolC LolE LolD
Heme CcmD CcmC CcmB CcmA
Lipopolysaccharide LptF LptG LptB
Fluoroquinolones Rv2686c Rv2687c Rv2688c
YydF peptide YydJ YydI
ABC-2 -type components without transporting function
Bacitracin BraE BraD Bacitracin resistance
Cationic antimicrobial peptide (CAMP) VraG VraF CAMP resistance
NosY NosF Cu2+-processing for N2O reductase
FtsX FtsE Cellar division involvment
YtrF YtrC/D YtrB YtrE Acetoin utilization
Eukaryotic-type ABC transporters
ABCA Subfamily
ABCA1 ABCA5
ABCA2 ABCA6
ABCA3 ABCA8
ABCA4 ABCA9
ABCA7 ABCA10
ABCA12
ABCA13
ABCB Subfamily
ABCB2 ABCB1 ABCB6 ABCB11 IrtA/B
ABCB3 ABCB4 ABCB7 MsbA
ABCB8 ABCB5 ATM MdlA/B
ABCB9 Rv0194
ABCB10 IroC
HlyB
RaxB
CylB
AbcA
PatA/B
SteA/B
VcaM
EfrA/B
ABCC Subfamily
ABCC1 ABCC7 ABCC8 LapB
ABCC2 ABCC9 HasD
ABCC3 PrsD
ABCC4 RsaD
ABCC5 EexD
ABCC6 ComA
ABCC10 BlpA
ABCC11 CydC/D
ABCC12
ABCC13
ABCD Subfamily
ABCD1 PXA1/2
ABCD2
ABCD3
ABCD4
ABCG Subfamily
ABCG1 ABCG2 ABCG5 PDR5
ABCG4 ABCG3 ABCG8 SNQ2
Macrolide exporters
MacB TylC MsrA VgaA Lsa
Other putative ABC transporters
YojI
FvdE
SyrD
YddA
02010 7/20/16
(c) Kanehisa Laboratories
log2 Normalized Abundance
-4.3 13.4
log2 Normalized Richness
1.1 13.4
(Reads/Gigabase pair)
E.C. #
60 70 85
GBS Site
E.C. containing at least one
#.#.#.#
differentially abundant KO

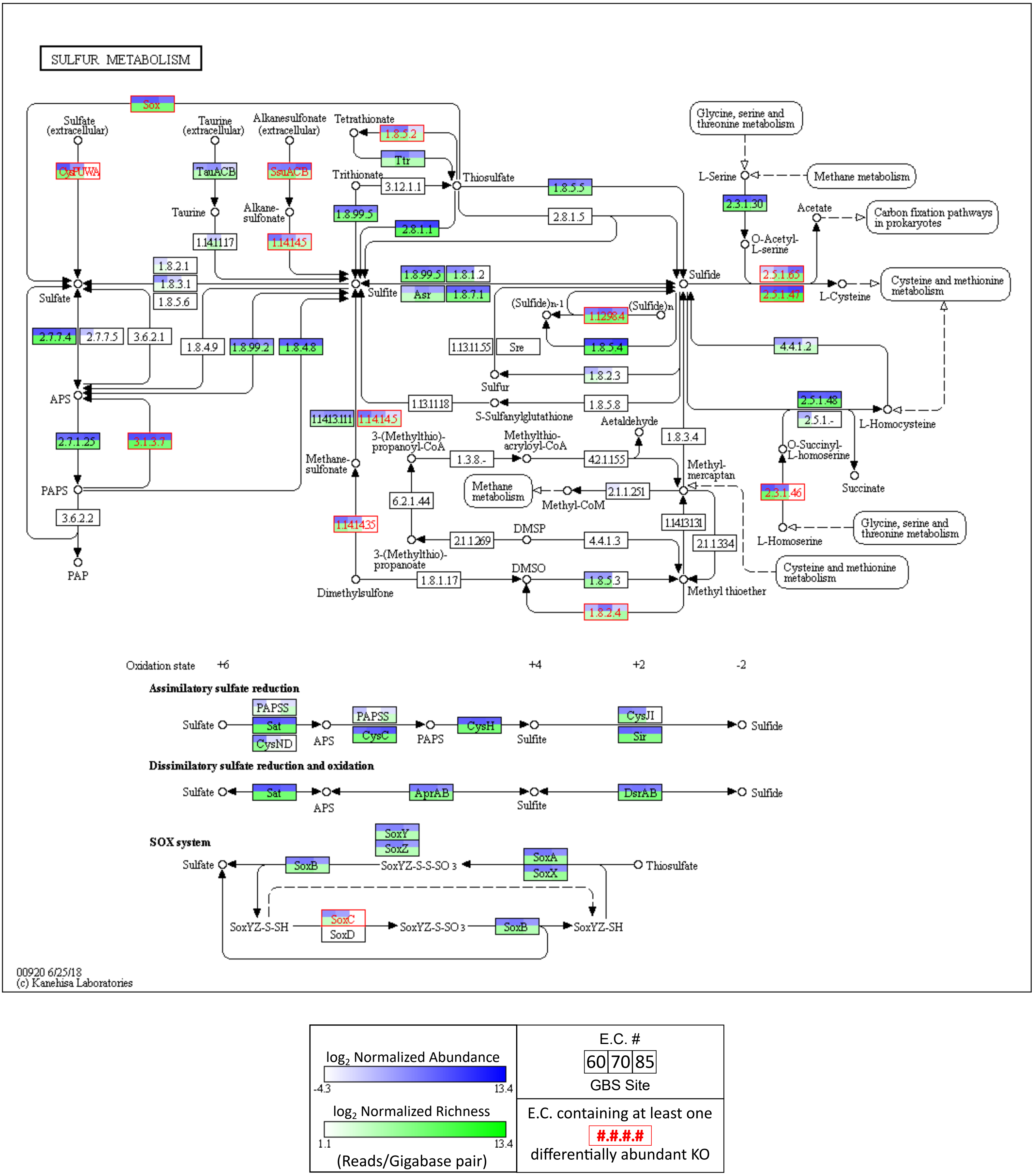
SULFUR METABOLISM
Sox
Sulfate (extracellular)
Taurine (extracellular)
Alkanesulfonate (extracellular)
Tetrathionate
CysPUWA
TauACB
SsuACB
1.8.5.2
Ttr
Trithionate
3.12.1.1
Thiosulfate
1.8.5.5
2.8.1.5
Taurine
Alkane-sulfonate
1.8.99.5
2.8.1.1
1.14.11.17
1.14.14.5
1.8.2.1
1.8.3.1
1.8.5.6
Sulfate
Sulfite
Sulfide
1.8.99.5
1.8.1.2
Asr
1.8.7.1
2.7.7.4
2.7.7.5
3.6.2.1
1.8.4.9
1.8.99.2
1.8.4.8
APS
2.7.1.25
3.1.3.7
PAPS
3.6.2.2
PAP
1.13.11.55
Sre
(Sulfide)n-1
(Sulfide)n
1.12.98.4
1.8.5.4
1.8.2.3
Sulfur
1.13.11.18
1.8.5.8
S-Sulfanylglutathione
1.14.13.111
1.14.14.5
Methane-sulfonate
3-(Methylthio)-propanoyl-CoA
1.3.8.-
Methylthio-acryloyl-CoA
4.2.1.155
Acetaldehyde
1.8.3.4
Methyl-mercaptan
Methane metabolism
Methyl-CoM
2.1.1.251
6.2.1.44
1.14.13.131
1.14.14.35
DMSP
2.1.1.269
4.4.1.3
2.1.1.334
3-(Methylthio)-propanoate
DMSO
Dimethylsulfone
1.8.1.17
1.8.5.3
Methyl thioether
1.8.2.4
Glycine, serine and threonine metabolism
L-Serine
Methane metabolism
2.3.1.30
Acetate
Carbon fixation pathways in prokaryotes
O-Acetyl-L-serine
2.5.1.65
2.5.1.47
L-Cysteine
Cysteine and methionine metabolism
4.4.1.2
2.5.1.48
2.5.1.-
L-Homocysteine
O-Succinyl-L-homoserine
2.3.1.46
Succinate
L-Homoserine
Glycine, serine and threonine metabolism
Cysteine and methionine metabolism
Oxidation state +6 +4 +2 -2
Assimilatory sulfate reduction
Sulfate
PAPSS
Sat
CysND
APS
PAPSS
CysC
PAPS
CysH
Sulfite
CysJI
Sir
Sulfide
Dissimilatory sulfate reduction and oxidation
Sulfate
Sat
APS
AprAB
Sulfite
DsrAB
Sulfide
SOX system
Sulfate
SoxB
SoxY
SoxZ
SoxYZ-S-S-SO3
SoxA
SoxX
Thiosulfate
SoxYZ-S-SH
SoxC
SoxD
SoxYZ-S-SO3
SoxB
SoxYZ-SH
00920 6/25/18
(c) Kanehisa Laboratories
log2 Normalized Abundance
-4.3
13.4
log2 Normalized Richness
1.1
13.4
(Reads/Gigabase pair)
E.C. #
60 70 85
GBS Site
E.C. containing at least one
#.#.#.#
differentially abundant KO

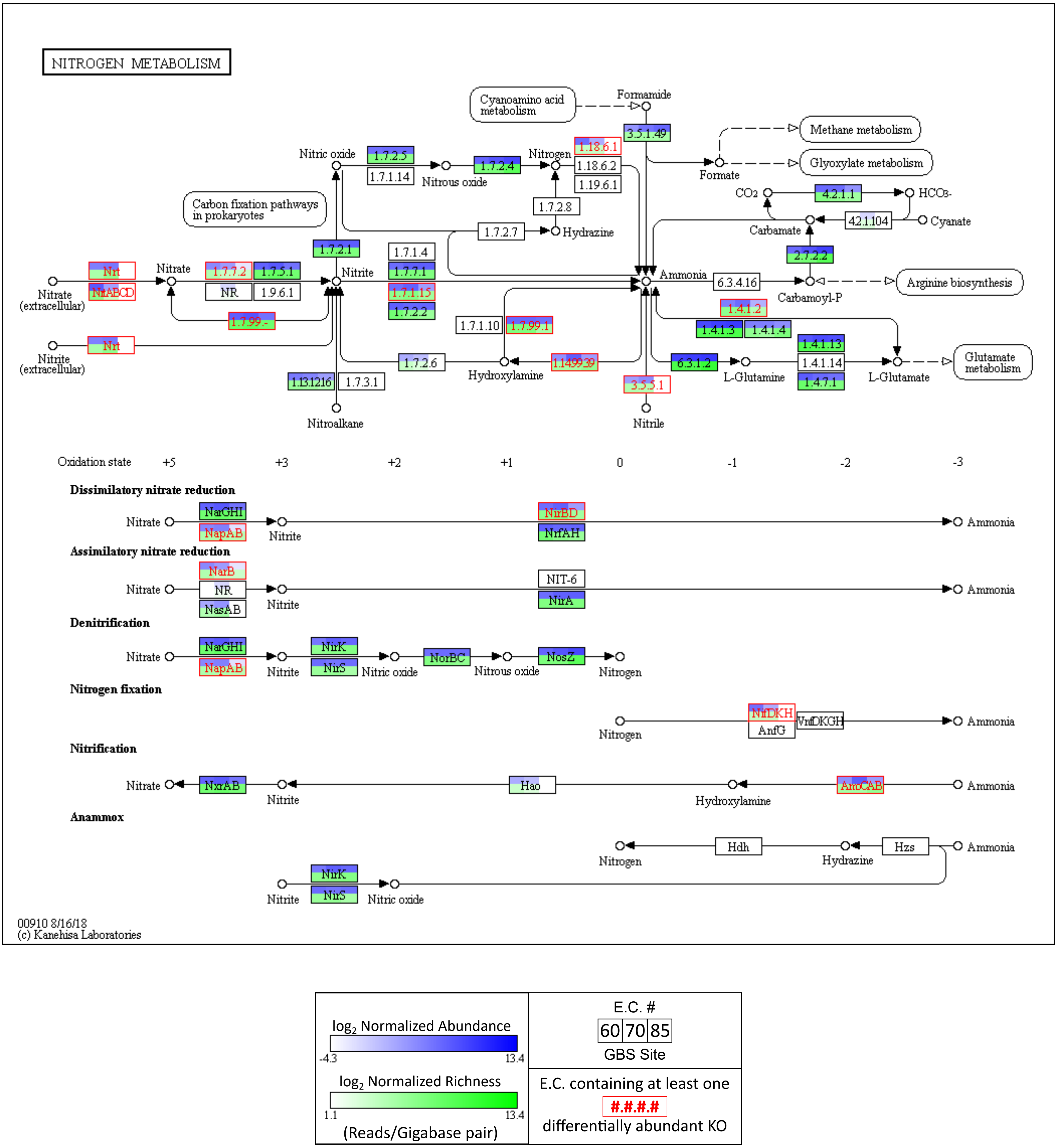
NITROGEN METABOLISM
Cyanoamino acid metabolism
Formamide
3.5.1.49
Methane metabolism
Glyoxylate metabolism
Formate
Nitric oxide
1.7.2.5
1.7.1.14
Nitrous oxide
1.7.2.4
Nitrogen
1.18.6.1
1.18.6.2
1.19.6.1
1.7.2.8
Carbon fixation pathways in prokaryotes
1.7.2.7
Hydrazine
CO2
4.2.1.1
HCO3-
Carbamate
4.2.1.104
Cyanate
2.7.2.2
1.7.2.1
1.7.1.4
1.7.7.1
1.7.1.15
1.7.2.2
Nitrate (extracellular)
Nrt
NrtABCD
Nitrate
1.7.7.2
1.7.5.1
NR
1.9.6.1
Nitrite
Ammonia
6.3.4.16
Carbamoyl-P
Arginine biosynthesis
1.7.99.-
1.7.1.10
1.7.99.1
1.4.1.2
1.4.1.3
1.4.1.4
1.4.1.13
1.4.1.14
1.4.7.1
Nitrite (extracellular)
1.7.2.6
Hydroxylamine
1.14.99.39
6.3.1.2
L-Glutamine
L-Glutamate
Glutamate metabolism
1.13.12.16
1.7.3.1
3.5.5.1
Nitroalkane
Nitrile
Oxidation state +5 +3 +2 +1 0 -1 -2 -3
Dissimilatory nitrate reduction
Nitrate NarGHI NapAB Nitrite NirBD NrfAH Ammonia
Assimilatory nitrate reduction
Nitrate NarB NR NasAB Nitrite NIT-6 NirA Ammonia
Denitrification
Nitrate NarGHI NapAB Nitrite NirK NirS Nitric oxide NorBC Nitrous oxide NosZ Nitrogen
Nitrogen fixation
Nitrogen NifDKH AnfG VnfDKGH Ammonia
Nitrification
Nitrate NxrAB Nitrite Hao Hydroxylamine AmoCAB Ammonia
Anammox
Nitrogen Hdh Hydrazine Hzs Ammonia
Nitrite NirK NirS Nitric oxide
00910 8/16/18
(c) Kanehisa Laboratories
log2 Normalized Abundance
-4.3
13.4
log2 Normalized Richness
1.1
13.4
(Reads/Gigabase pair)
E.C. #
60 70 85
GBS Site
E.C. containing at least one
#.#.#.#
differentially abundant KO

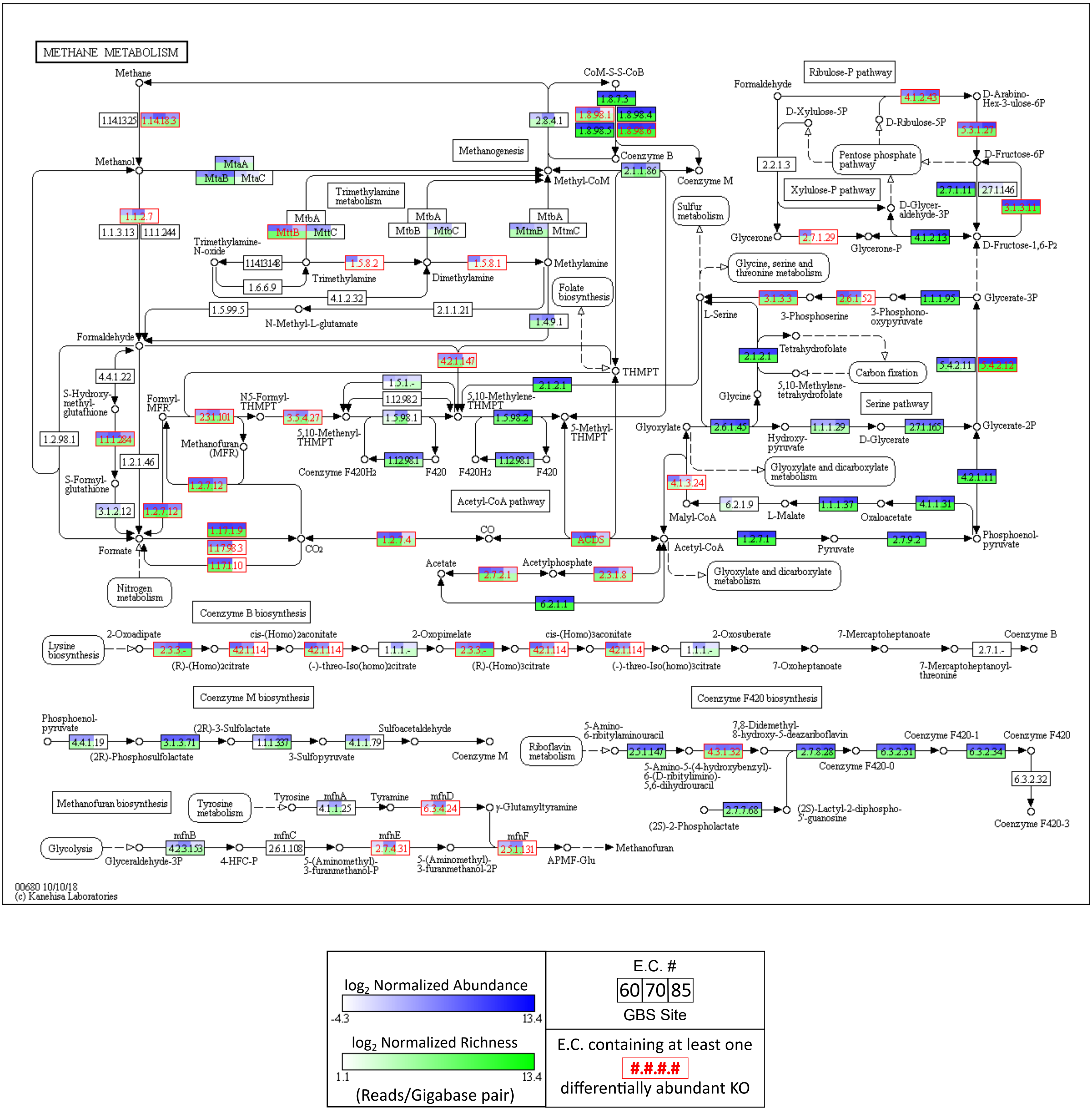
METHANE METABOLISM
Methane
CoM-S-S-CoB
Ribulose-P pathway
Formaldehyde
D-Arabino-Hex-3-ulose-6P
D-Xylulose-5P
D-Ribulose-5P
1.14.13.25
1.14.18.3
2.8.4.1
1.8.7.3
1.8.98.1
1.8.98.4
1.8.98.5
1.8.98.6
4.1.2.43
5.3.1.27
Methanogenesis
Coenzyme B
D-Fructose-6P
Methanol
MtaA
MtaB
MtaC
2.1.1.86
Coenzyme M
2.2.1.3
Pentose phosphate pathway
Trimethylamine metabolism
Methyl-CoM
Xylulose-P pathway
2.7.1.11
2.7.1.146
Sulfur metabolism
D-Glyceraldehyde-3P
4.1.2.13
1.1.2.7
MttB
MttC
MtbA
MtbB
MtbC
MtmB
MtmC
1.1.3.13
1.1.2.44
Trimethylamine-N-oxide
1.14.13.148
1.5.8.2
1.5.8.1
Methylamine
Glycerone
2.7.1.29
Glycerone-P
D-Fructose-1,6-P2
Trimethylamine
Dimethylamine
Glycine, serine and threonine metabolism
1.6.6.9
4.1.2.32
Folate biosynthesis
3.1.3.3
2.6.1.52
1.1.1.95
Glycerate-3P
1.5.99.5
2.1.1.21
L-Serine
3-Phosphoserine
3-Phospho-oxypyruvate
N-Methyl-L-glutamate
1.4.9.1
Formaldehyde
Tetrahydrofolate
4.2.1.147
2.1.2.1
THMPT
5.4.2.11
5.4.2.12
4.4.1.22
1.5.1.-
5,10-Methylene-tetrahydrofolate
Carbon fixation
S-Hydroxy-methyl-glutathione
Formyl-MFR
N5-Formyl-THMPT
1.12.98.2
5,10-Methylene-THMPT
2.1.2.1
Glycine
Serine pathway
2.3.1.101
3.5.4.27
1.5.98.1
1.5.98.2
5-Methyl-THMPT
Glyoxylate
2.6.1.45
1.1.1.29
2.7.1.165
Glycerate-2P
1.2.98.1
1.1.1.284
Methanofuran (MFR)
5,10-Methenyl-THMPT
1.12.98.1
Hydroxy-pyruvate
D-Glycerate
Coenzyme F420H2
F420
F420H2
Glyoxylate and dicarboxylate metabolism
1.2.1.46
4.1.3.24
4.2.1.11
S-Formyl-glutathione
1.2.7.12
Acetyl-CoA pathway
6.2.1.9
1.1.1.37
4.1.1.31
3.1.2.12
Malyl-CoA
L-Malate
Oxaloacetate
1.17.1.9
CO
Phosphoenol-pyruvate
1.17.98.3
1.2.7.4
ACDS
1.2.7.1
2.7.9.2
Formate
CO2
Acetyl-CoA
Pyruvate
1.17.1.10
Acetate
Acetylphosphate
2.7.2.1
2.3.1.8
Glyoxylate and dicarboxylate metabolism
Nitrogen metabolism
6.2.1.1
Coenzyme B biosynthesis
Lysine biosynthesis
2-Oxoadipate
cis-(Homo)2aconitate
2-Oxopimelate
cis-(Homo)3aconitate
2-Oxosuberate
7-Mercaptoheptanoate
Coenzyme B
2.3.3.-
4.2.1.114
1.1.1.-
2.7.1.-
(R)-(Homo)2citrate
(-)-threo-Iso(homo)2citrate
(R)-(Homo)3citrate
(-)-threo-Iso(homo)3citrate
7-Oxoheptanoate
7-Mercaptoheptanoyl-threonine
Coenzyme M biosynthesis
Coenzyme F420 biosynthesis
Phosphoenol-pyruvate
(2R)-3-Sulfolactate
Sulfoacetaldehyde
4.4.1.19
3.1.3.71
1.1.1.337
4.1.1.79
(2R)-Phosphosulfolactate
3-Sulfopyruvate
Coenzyme M
5-Amino-6-ribitylaminouracil
7,8-Didemethyl-8-hydroxy-5-deazariboflavin
Coenzyme F420-1
Coenzyme F420
Riboflavin metabolism
2.5.1.147
4.3.1.32
2.7.8.28
6.3.2.31
6.3.2.34
Coenzyme F420-0
5-Amino-5-(4-hydroxybenzyl)-6-(D-ribitylimino)-5,6-dihydrouracil
6.3.2.32
Methanofuran biosynthesis
Tyrosine metabolism
Tyrosine
mfnA
4.1.1.25
Tyramine
mfnD
6.3.4.24
γ-Glutamyltyramine
2.7.7.68
(2S)-Lactyl-2-diphospho-5'-guanosine
(2S)-2-Phospholactate
Coenzyme F420-3
Glycolysis
mfnB
4.2.3.153
mfnC
2.6.1.108
mfnE
2.7.4.31
mfnF
2.5.1.131
Methanofuran
Glyceraldehyde-3P
4-HFC-P
5-(Aminomethyl)-3-furanmethanol-P
5-(Aminomethyl)-3-furanmethanol-2P
APMF-Glu
00680 10/10/18
(c) Kanehisa Laboratories
log2 Normalized Abundance
-4.3
13.4
log2 Normalized Richness
1.1
13.4
(Reads/Gigabase pair)
E.C. #
60
70
85
GBS Site
E.C. containing at least one
#.#.#.#
differentially abundant KO
